# Biotransformation of Δ^1^-Progesterone Using Selected Entomopathogenic Filamentous Fungi and Prediction of Its Products’ Bioactivity

**DOI:** 10.3390/ijms25010508

**Published:** 2023-12-29

**Authors:** Anna Panek, Patrycja Wójcik, Alina Świzdor, Maciej Szaleniec, Tomasz Janeczko

**Affiliations:** 1 Department of Food Chemistry and Biocatalysis, Wrocław University of Environmental and Life Sciences, Norwida 25, 50-375 Wrocław, Poland; alina.swizdor@upwr.edu.pl; 2 Jerzy Haber Institute of Catalysis and Surface Chemistry, Polish Academy of Sciences, Niezapominajek 8, 30-239 Krakow, Poland; patrycwoj@gmail.com (P.W.); maciej.szaleniec@ikifp.edu.pl (M.S.)

**Keywords:** biotransformation, pregnanes, Δ^1^-steroids, entomopathogenic filamentous fungi, hydroxylation

## Abstract

This research aimed at obtaining new derivatives of pregn-1,4-diene-3,20-dione (Δ^1^-progesterone) (**2**) through microbiological transformation. For the role of catalysts, we used six strains of entomopathogenic filamentous fungi (*Beauveria bassiana* KCh J1.5, *Beauveria caledonica* KCh J3.3, *Isaria fumosorosea* KCh J2, *Isaria farinosa* KCh KW1.1, *Isaria tenuipes* MU35, and *Metarhizium robertsii* MU4). The substrate (**2**) was obtained by carrying out an enzymatic 1,2-dehydrogenation on an increased scale (3.5 g/L) using a recombinant cholest-4-en-3-one Δ^1^-dehydrogenase (AcmB) from *Sterolibacterium denitrificans*. All selected strains were characterized by the high biotransformation capacity for the used substrate. As a result of the biotransformation, six steroid derivatives were obtained: 11α-hydroxypregn-1,4-diene-3,20-dione (**3**), 6β,11α-dihydroxypregn-1,4-diene-3,20-dione (**4**), 6β-hydroxypregn-1,4-diene-3,11,20-trione (**5**), 6β,17α-dihydroxypregn-1,4-diene-3,20-dione (**6**), 6β,17β-dihydroxyandrost-1,4-diene-3-one (**7**), and 12β,17α-dihydroxypregn-1,4-diene-3,20-dione (**8**). The results show evident variability of the biotransformation process between strains of the tested biocatalysts from different species described as entomopathogenic filamentous fungi. The obtained products were tested in silico using cheminformatics tools for their pharmacokinetic and pharmacodynamic properties, proving their potentially high biological activities. This study showed that the obtained compounds may have applications as effective inhibitors of testosterone 17β-dehydrogenase. Most of the obtained products should, also with a high probability, find potential uses as androgen antagonists, a prostate as well as menopausal disorders treatment. They should also demonstrate immunosuppressive, erythropoiesis-stimulating, and anti-inflammatory properties.

## 1. Introduction

Progesterone is one of the key steroid hormones whose task is to regulate female reproductive functions, largely controlled in many organs such as the uterus, ovaries, mammary glands, and brain [1]. Progesterone is synthesized mainly in the ovaries, placenta, and adrenal glands [2]. Apart from securing the normal course of pregnancy, progesterone also controls most reproductive processes in women, including oocyte maturation, ovulation, menstruation, facilitating the implantation of the embryo in the uterus, uterine growth, the inhibition of myometrial contractions, the development of the mammary gland, the regulation of milk production, and sexual behavior [1,3].

Although the role of progesterone in humans is well known, the possible activity and implications of progesterone metabolites have yet to be determined. There is increasing evidence that many metabolites are not inactive but have important biological effects [4,5,6]. Progesterone is rapidly and extensively metabolized in the human body, mainly in the liver, but also in the brain, skin, and various other extrahepatic tissues [7,8]. Progesterone exhibits a remarkably short elimination half-life of approximately 5 min in the circulatory system. The metabolism of progesterone is intricate, giving rise to a potential formation of up to 35 distinct unconjugated metabolites upon oral ingestion [9]. The primary metabolic pathway of progesterone involves a reduction by 5α-reductase and 5β-reductase, leading to the formation of the dihydrogenated products, namely 5α-dihydroprogesterone and 5β-dihydroprogesterone, respectively [10,11,12].

In the human organism a relatively small amount of progesterone (**1**) is hydroxylated by 17α-hydroxylase (CYP17A1) and 21-hydroxylase (CYP21A2) to become 17α-hydroxyprogesterone and 21-hydroxyprogesterone, respectively [13,14]. Even smaller amounts of progesterone can also be hydroxylated by 11β-hydroxylase (CYP11B1), and to a lesser extent by aldosterone synthase (CYP11B2), into 11β-hydroxyprogesterone [15]. Moreover, progesterone can be hydroxylated in the human liver by other cytochrome P450 enzymes that are not specific to steroids [16]. 6β-Hydroxylation, which is catalyzed mainly by CYP3A4, is the main transformation and accounts for approximately 70% of the progesterone metabolism mediated by this enzyme [16]. Other pathways include 6α-, 16α-, and 16β-hydroxylation [13].

Over the course of more than 70 years of research, it has been observed that the biotransformation of progesterone by filamentous fungi can proceed in two main directions: hydroxylation or Baeyer–Villiger oxidation, and rarely does a combination of these processes occur [17,18,19]. Hydroxylation predominantly occurs at the 6β and/or 11α positions; however, fungal cultures have also demonstrated the capability for hydroxylation at the 14α, 7α, 7β, 11β, 17α, 9α, 8β, 16α, 15α, 15β, 21, and 12β positions [20,21,22]. The Baeyer–Villiger oxidation of progesterone is possible through the biotransformation by some *Aspergillus* or *Penicillium* species [23,24,25,26].

The introduction of a double bond between the C1 and C2 positions in the steroid structure has been found to enhance the metabolic stability against enzymes such as 5α-reductase and aromatase [27,28]. Notably, even in the early stages of research, it was observed that the medicinal properties of several Δ^1^-3-ketosteroids were more potent compared to their corresponding 1(2)-saturated analogues [29,30]. This also refers to currently used medications. For instance, the anti-inflammatory efficacy of prednisolone is four times greater than that of hydrocortisone, and prednisone is five times more potent than cortisone [31]. Δ^1^-3-Ketosteroids demonstrate diverse essential biological activities, contributing to their widespread use in medicine and establishing a significant presence in the pharmaceutical industry [28].

The primary objective of this study was to delineate the spectrum of hydroxylated derivatives arising from the bioconversion of pregn-1,4-diene-3,20-dione (Δ^1^-progesterone) (**2**). In addition, our aim was to investigate the potential biological activities associated with the selected hydroxy derivatives, utilizing state-of-the-art cheminformatic tools. As a result of the biotechnological functionalization, we obtained new Δ^1^-3-ketopregnanes with high potential biological activity. The substrate (**2**) was obtained by increasing the scale of the previously described method utilizing cholest-4-en-3-one Δ^1^-dehydrogenase (AcmB) from *Sterolibacterium denitrificans*, a key enzyme of the central degradation pathway of cholesterol [32]. Further, biocatalysts of hydroxylation were used including strains of entomopathogenic filamentous fungi. The *Isaria farinosa* KCh KW 1.1 strain possesses the ability to effectively dihydroxylate progesterone and its derivatives [17,33]. *Isaria fumosorosea* KCh J2 is known for its ability to carry out multienzymatic transformations of steroid compounds [34,35]. We also decided to use the *Beauveria bassiana* KCh J1.5 strain, which is a representative of the species of entomopathogenic filamentous fungi most often used for the biotransformation of steroid compounds [36,37,38,39] and the *Beauveria caledonica* KCh J3.3 strain belonging to the same genus [36]. Two strains of species with relatively little known catalytic abilities were also used as biocatalysts: *Metarhizium anisopliae*, capable of hydroxylating 13-ethyl-gon-4-ene-3,17-dione [40], and *Isaria tenuipes*, known as the zearalenone biocatalyst [41].

## 2. Results and Discussion

The underlying aim of this research was the verification of the ability of six strains of entomopathogenic filamentous fungi (belonging to six different species) to transform progesterone with an additional double bond located between the C-1 and C-2 carbon atoms and to obtain new derivatives of pregn-1,4-diene-3,20-dione (Δ^1^-progesterone) (**2**). The substrate (**2**) (Spectral data presented in Supplementary Materials Figures S3–S7) was obtained by carrying out an enzymatic 1,2-dehydrogenation on an increased scale (3.5 g/L) using a recombinant cholest-4-en-3-one Δ^1^-dehydrogenase (AcmB) from *Sterolibacterium denitrificans* [32] and phenazine methosulfate as an electron acceptor. The 1(2)-dehydrogenation proceeded efficiently under anaerobic conditions and a 100% conversion of **1** to **2** was obtained after 5.5 h of reaction. As biocatalysts for the subsequent transformations, the following strains of entomopathogenic filamentous fungi were used: *Beauveria bassiana* KCh J1.5, *Beauveria caledonica* KCh J3.3, *Isaria fumosorosea* KCh J2, *Isaria farinosa* KCh KW1.1, and *Isaria tenuipes* MU35, *Metarhizium robertsii* MU4 (formerly known as *Metarhizium anisopliae* [42]). Based on TLC and GC analyses, it was observed that the substrate was effectively transformed in the cultures of the tested strains (Table 1). The resulting products are more polar than the substrate (**2**), and the composition of the product mixture in the cultures of most of the tested biocatalysts varies during incubation.

**Figure 1:**
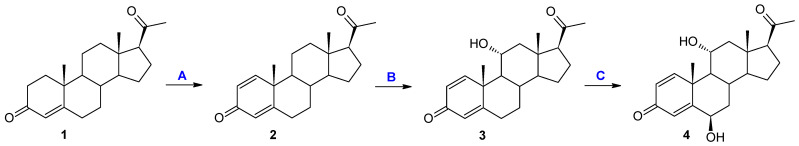
Transformation of progesterone: (A) enzyme; (B) and (C) in a culture of *Metarhizium robertsii* MU4 and *Beauveria caledonica* KCh J3.3.

In the culture of the *Metarhizium robertsii* MU4 strain, one main product was obtained accompanied by a compound formed after 7 days of the transformation process in a small amount (approximately 4%). Based on TLC and GC analyses, after 1 and 3 days of biotransformation, compound **3** was identified as the only product, with a complete reaction of the substrate (Figure 1). After the biotransformation on an increased scale, it was isolated with high efficiency—90%. After performing NMR analyses, the structure of this compound was determined to be 11α-hydroxypregn-1,4-diene-3,20-dione (**3**). In the ^1^H NMR spectrum performed for this compound, a characteristic signal was observed at 4.07 ppm. The HSQC correlation spectrum shows the coupling of this signal with the carbon signal located at 67.97 ppm. The positions of both signals indicate that the obtained compound is a hydroxylation product. Slight shifts of the remaining signals, both in the ^1^H NMR and ^13^C NMR spectra (Table 2), clearly indicate that one hydroxyl group has been introduced into the structure of the analyzed compound. Based on the analysis of the couplings of the signal at 4.07 ppm with the signals originating from the H-9, H-12α, and H-12β protons visible in the COSY spectrum and the couplings of this signal with the signals originating from the C-9, C-10, and C-12 carbons, the structure of the obtained compound can be unambiguously determined. The chemical shift and multiplicity of the signal originating from the proton H-11β unambiguously indicate the introduction of the hydroxyl group at the 11α position (Supplementary Materials: Figures S10–S14).

**Figure 2:**
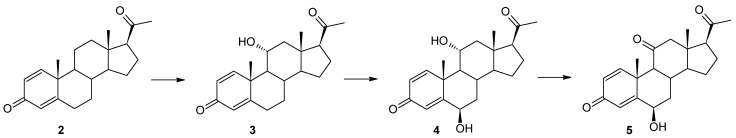
Cascade biotransformation of pregn-1,4-diene-3,20-dione (**2**) in *Beauveria bassiana* KCh J1.5 culture.

The 11α-hydroxylation is the most frequently reported transformation of progesterone and its derivatives during biotransformation in cultures of filamentous fungi [20,43,44,45,46,47,48,49,50]. However, in the vast majority of cases, apart from the 11α-hydroxy derivative, other products were also identified [20,45,46,47,48,49,50]. In the culture of the *M. robertsii* MU4 strain, after the first and third day of transformation, it is possible to obtain only one compound with very high efficiency. Due to the small amounts of formation of the second product, the structure of compound **4** was determined as a result of the preparative biotransformation in the culture of the *Beauveria bassiana* KCh J1.5 strain. In the culture of this strain, after one day of biotransformation (analogously to the culture of the *M. robertsii* MU4 strain), one main product—11α-hydroxypregn-1,4-diene-3,20-dione (**3**)—was observed. However, after just three days of the transformation process, a dynamic change in the composition of the products was observed. Three products (**3**, **4**, and **5**) were visible in the reaction mixture, and after the seventh day only compounds **4** and **5** were identified (Table 1 and Figure 2). Based on the course of the biotransformation in the culture of the *B. bassiana* KCh J1.5 strain over time, it can be assumed that the transformations of the tested compound by *M. robertsii* MU4 are of a cascade nature (Figure 1). These assumptions were confirmed by the analysis of the structure of the resulting products. The ^1^H NMR spectrum obtained for compound **4** shows signals indicating the preservation of the basic skeleton of pregn-1,4-diene-3,20-dione. A characteristic signal at 3.93 ppm is also visible (as for product **3**), which in the COSY spectrum couples with signals coming from the H-9, H-12α, and H-12β protons. The ^13^C NMR spectrum shows two signals in a position characteristic of signals coming from carbon atoms connected with the hydroxyl group: 66.37 ppm coming from carbon C-11 and the second in the position of 72.10 ppm. The latter signal couples in the HMBC spectrum with signals coming from the H-4 proton and, weakly, from the H-1 proton, and in the COSY spectrum with signals coming from H-7α and H-7β protons. Additionally, the characteristic multiplicity and shifts of the methyl groups at C-18, C-19, and C-21 clearly indicate that in the structure of the isolated compound of the hydroxyl group is also located in the 6β position. On this basis, compound **4** was characterized as 6β,11α-dihydroxypregn-1,4-diene-3,20-dione (**4**) (Supplementary Materials: Figures S17–S21). Based on the ^1^H NMR spectrum obtained for compound **5**, it was found that it possesses the structure of pregn-1,4-diene-3,20-dione and (like compound **4**) a hydroxyl group at carbon C-6. However, based on the ^13^C NMR spectrum (Table 2), it was determined that it also contains three carbonyl groups in its structure. A clear shift towards a lower field of the signal coming from the protons of the H-19 methyl group and the analysis of the biotransformation of compound **2** in the culture of the *B. bassiana* KCh J1.5 strain (compound **5** is formed from compound **4**) allow us to clearly determine the structure of this product to be 6β-hydroxypregn-1,4-diene-3,11,20-trione (**5**) (Supplementary Materials: Figures S24–S28). On this basis, the course of the transformations of the tested substrate in the culture of the *B. bassiana* KCh J1.5 strain can be determined as follows: the first stage is the hydroxylation leading to the 11α-hydroxyl derivative (**3**), which undergoes further hydroxylation leading to the 6β,11α-dihydroxy derivative, and the last step is the oxidation of the hydroxyl group at carbon C-11, leading to the formation of compound **5** (Figure 2). An analogous cascade course was previously described during the biotransformation of progesterone leading to 6β-hydroxy-11-oxoprogesterone in cultures of *Mucor* M881 strains [51] and *Isaria farinosa* KCh KW1.1 [17,33]. On the other hand, the ability to dihydroxylate progesterone leading to the 6β,11α-dihydroxy derivative was previously observed in cultures of the following strains: *Aspergillus nidulans* VKPM F-1069 [45], *A. niger* N402 [43], *A. ochraceus* [49], *Rhizomucor pusillus* and *Absidia griseolla* var. *igachii* [48], *Rhizomucor tauricus* IMI23312 [50], *Cephalosporium aphidicola* [46], and *Trichoderma koningii*, *T. hamatum*, *T. aureoviride*, and *T. pseudokoningii* [20].

A similar course was observed in the culture of the *Beauveria caledonica* KCh J3.3 strain, although, only in the culture of this strain, an unreacted substrate was observed after one day (almost 30% of the reaction mixture). However, after the third day of the transformation process, in addition to the hydroxylation products 11α and 6β,11α, other products were also observed. Unfortunately, their large number and individual percentages did not allow for their isolation and a determination of their structure. Additionally, a progressive decrease over time in the identified compounds can be observed, which may indicate degradation processes taking place in the cells of the *Beauveria caledonica* KCh J3.3 strain. An even greater number of products formed were observed during the incubation of pregn-1,4-diene-3,20-dione (Δ^1^-progesterone) (**2**) in the culture of the *Isaria tenuipes* MU35 strain. After just one day, the reaction of the substrate was observed to be complete and a multitude of products were observed both on TLC and GC. Due to the large number of compounds formed, attempts to isolate them were not pursued. Nevertheless, the obtained result indicates that the tested biocatalyst produces a multitude of enzymes capable of transforming steroid compounds and indicates the possibility of using this strain in the biocatalysis of various bioactive compounds; e.g., in our previous work, we described the effective glycosylation of quercetin by *Isaria tenuipes* MU35 [52]. It was also proven that a strain of this species is capable of an effective metabolism of zearalenone and, similar to the two representatives of this genus more popular in biocatalysis (*I. fumosorosea*, *I. farinosa*), multienzymatic transformations of the used substrate were observed [41]. Moreover, *Isaria tenuipes* are important entomopathogenic fungi used in health foods and traditional herbal medicines in East Asia, thus a source of valuable metabolites [53,54].

**Figure 3:**
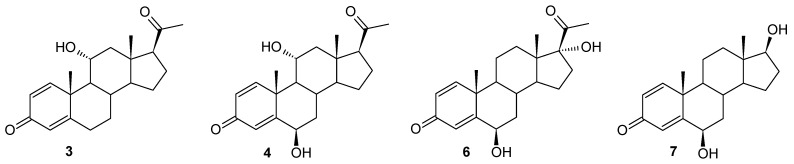
Products identified during the transformation of compound **2** in the culture of the *Isaria fumosorosea* strain KCh J2.

The *Isaria fumosorosea* KCh J2 strain has already been described in previous manuscripts as a biocatalyst capable of producing many effective enzymes involved in the transformation of the substrates used. It is known for its effective cascade reactions of flavonoid compounds (demethylation, hydroxylation, oxidation of hydroxyl groups, methylglycosidation) [55], steroid compounds—dehydroepiandrosterone (DHEA) (hydroxylation, Baeyer–Villiger oxidation) [34]—and estrones (hydroxylation, oxidation of hydroxyl groups, methylglycosidation, Baeyer–Villiger oxidation) [35]. However, the high activity of these many enzymes led to the formation of many products during the biotransformation of progesterone, which consequently prevented their separation [34]. During the transformation of pregn-1,4-diene-3,20-dione (**2**) in the culture of this strain, dynamic transformations of the resulting compounds were also observed, but in this case it was possible to determine the structure of most of the resulting metabolites. In the culture of the *I. fumosorosea* KCh J2 strain, a hydroxylation effect was observed, leading to 11α-hydroxypregn-1,4-diene-3,20-dione (**3**) and 6β,11α-dihydroxypregn-1,4-diene-3,20-dione (**4**)—a transformation pathway observed in the cultures of previously described strains of entomopathogenic fungi (*B. bassiana* KCh J1.5, *B. caledonica* KCh J3.3, and *M. robertsii* MU4). However, in parallel, the following were identified as the dominant products: 6β,17α-dihydroxypregn-1,4-diene-3,20-dione (**6**) and 6β,17β-dihydroxyandrost-1,4-diene-3-one (**7**) (Figure 3). It was observed in the ^1^H NMR spectrum that the skeleton of pregn-1,4-diene-3,20-dione in compound **6** was retained. The presence of a signal at 4.53 ppm indicates the location of a hydroxyl group present at the secondary carbon atom. The presence of this functionalization is confirmed by the coupling of this signal with the signal located at 74.00 ppm, visible in the HMQC spectrum. Additionally, this signal visible in the ^13^C NMR spectrum is coupled in the HMBC spectrum with the signal coming from the H-4 proton, which indicates that the C-6 carbon atom has been hydroxylated. The position and multiplicity of the signal visible in the ^1^H NMR spectrum and the position of the C-18 and C-19 methyl groups indicate that the hydroxyl group is located in the β position. Additionally, the shift of the C-21 methyl group towards a higher field indicates a structural modification of compound **6** within the D ring. The location of the signal coming from the C-17 carbon at 89.79 ppm (^13^C NMR spectrum) and the coupling of this signal with the signal coming from the C-18 methyl protons visible on the HMBC spectrum indicate that carbon atom C-17 has undergone hydroxylation as well. Compound **6** is therefore a dihydroxy derivative of the substrate used (Supplementary Materials: Figures S31–S35).

Analyzing the course of the biotransformation of pregn-1,4-diene-3,20-dione (**2**) in the culture of the *I. fumosorosea* KCh J2 strain, one can notice that the percentage of 6β,17α-dihydroxypregn-1,4-diene-3,20-dione (**6**) decreases during biotransformation, while the amount of compound **7** increases (exceeding the value of 50% after seven days of reaction). This indicates a high probability that compound **6** is an intermediate during the formation of compound **7** (Table 1). Based on the insight provided by the NMR spectra, it was determined that, compared to compound **6**, in the structure of compound **7**, the structural modification occurred only within the D ring. The absence of a signal in the ^1^H NMR and ^13^C NMR spectra coming from the C-21 methyl group and the presence of a triplet at 3.64 ppm (coming from proton H-17α visible on the ^1^H NMR), which couples with the signal coming from carbon C-17 in the HMQC spectrum, clearly indicate that the obtained product is 6β,17β-dihydroxyandrost-1,4-diene-3-one (**7**), i.e., 6β-hydroxy-Δ^1^-testosterone (Supplementary Materials: Figures S31–S35). Similar mechanisms are known in the available literature, involving subsequent oxidations leading to the degradation of the acyl substituent located at the C-17 carbon of progesterone. Over the past decades, the microbial production of 17α-hydroxyprogesterone (17-HP) using specific strains (e.g., *Curvularia lunata* ATCC 12017, *Cunninghamella blakesleeana* ATCC 8688a, and *Cladosporium* sp. F 5394) [56] has attracted increasing attention due to its environmental benefits, flexible production conditions and high catalytic efficiency [57]. During hydroxylation, the progesterone-17α-hydroxylase belonging to cytochrome P450 monooxygenases (CYP) converts progesterone to 17-HP [57,58]. In mammals, including humans, there is only one CYP17A (CYP17A1), which plays a key role in the oxidation of progesterone to androstenedione [59,60]. CYP17A1 catalyzes two different oxidase reactions of a mixed function: the first step is the hydroxylation reaction at the C17 position of progesterone to produce 17-HP, followed by the cleavage of the C17,20 bond to produce testosterone and androstenedione [60,61]. On the other hand, the 17α-hydroxylation reaction of progesterone and its derivatives is one of the most desirable transformations of steroid compounds leading to valuable products [57]. The 17α-hydroxyprogesterone caproate can be used to prevent premature birth, while 17-HP is used to treat amenorrhea, endometrial cancer, abnormal uterine bleeding, and premature birth [57,62,63].

**Figure 4:**
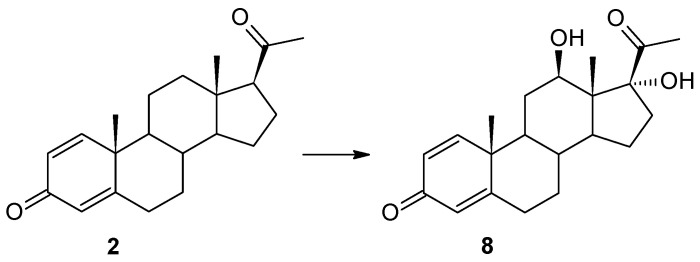
Biotransformation of pregn-1,4-diene-3,20-dione (**2**) in *Isaria farinosa* KCh KW 1.1 culture.

In our previous manuscripts, we described the ability of the *Isaria farinosa* KCh KW 1.1 strain to hydroxylate progesterone and its derivatives effectively, leading to 6β,11α-dihydroxy derivatives [17,33]. These metabolites were obtained during the transformation of progesterone, 11α-hydroxyprogesterone, and 16α,17α-epoxyprogesterone [17,33]. However, during the transformation of 17α-hydroxyprogesterone, we isolated three hydroxy derivatives: 6β,17α-dihydroxyprogesterone, 12β,17α-dihydroxyprogesta-1,4-diene-3-one, and 6β,12β,17α-trihydroxyprogesterone [17]. Surprisingly, as a result of using the progesterone with an additional double bond between the C1 and C-2 carbon atoms (Δ^1^-progesterone (**2**)) as a substrate, 12β,17α-dihydroxypregn-1,4-diene-3,20-dione (**8**) was obtained in the culture of this biocatalyst. In the ^1^H NMR spectrum, a characteristic position of the signal originating from the protons of the H-21 methyl group was observed: 2.26 ppm, indicating the hydroxylation of carbon C-17. The shift of the signal from this carbon towards the lower field to a value of 88.55 ppm confirms this assumption. The presence of a signal at 68.04 ppm in the ^13^C NMR spectrum, and the coupling of this signal (HMQC spectrum) with the signal visible at 3.86 ppm in the ^1^H NMR spectra, is evidence of the hydroxylation of the secondary carbon atom as well. The coupling visible in the HMBC spectrum of signals from both protons of the hydroxyl groups with the signal from carbon C-13 indicates that hydroxylation occurred at carbons C-12 and C-17. The shapes and positions of all the signals indicate that the isolated compound is 12β,17α-dihydroxypregn-1,4-diene-3,20-dione (**8**) (Supplementary Materials: Figures S40–S44). After a shorter biotransformation time, other steroid products were also observed in the culture of the *Isaria farinosa* KCh KW 1.1 strain. However, it was not possible to isolate them. Based on the structure of compound **8**, it can be assumed that two of them may be intermediate products leading to 12β,17α-dihydroxypregn-1,4-diene-3,20-dione (**8**). Their structure is shown in Figure 4. Based on the results obtained, both the *Isaria farinosa* KCh KW 1.1 and *Isaria fumosorosea* KCh J2 strains can be qualified for further research on the activity of monooxygenases, with a particular emphasis on CYP17A.

To evaluate the physicochemical properties, pharmacokinetics, and potential biological activity of Δ^1^-progesterone derivatives, cheminformatic tools such as SwissADME and passOnline were employed. Physicochemical descriptor calculations were conducted for both the substrate and the obtained products (**2**–**8**), as summarized in Table 3. Predictions encompassed their ADME parameters (absorption, distribution, metabolism, and excretion), pharmacokinetic properties, and suitability for medicinal chemistry. The analysis utilized the online tool SwissADME, accessible at http://www.swissadme.ch (accessed on 15 June 2023), developed and managed by the Molecular Modeling Group of the Swiss Institute of Bioinformatics (SIB) [64]. According to the results obtained from this tool, it was observed that all the products exhibited significantly lower lipophilicity and markedly higher water solubility compared to Δ^1^-progesterone (**2**) (Table 3). This shift is attributed to the introduction of polar -OH groups during the transformation.

According to the results we obtained from in silico studies, both substrates (controls)—progesterone (**1**) and Δ^1^-progesterone (**2**)—as well as the obtained products (**3**–**8**) can effectively penetrate the intestine/blood barrier in passive mode and should passively penetrate the blood/brain barrier [65]. Additionally, the model predicts that the tested compounds (**3**–**8**) may be efficiently removed from these compartments by the P-glycoprotein. We can observe that most of the obtained compounds are predicted to be removed by P-glycoprotein from the bloodstream and the central nervous system (Supplementary Materials: Figures S1, S8, S15, S22, S29, S36, S38 and S45). P-glycoprotein (P-gp), also known as multidrug resistance protein 1 (MDR1), is an efflux transporter that influences the absorption, distribution, and elimination of a variety of compounds [66]. The attachment of a hydroxy group impacts the stability and solubility of steroids and increases their hydrophilicity, thus affecting their bioavailability and bioactivity [67,68]. However, the dosage form of the steroid can also impact its bioavailability [69].

Naturally occurring sex steroids, such as estradiol, estrogen sulfates, and progesterone, or their derivatives pose challenges in determining bioavailability. These compounds demonstrate effective absorption but undergo rapid metabolism and elimination. Primarily used in hormone replacement therapy, they necessitate high doses—for instance, 2000 µg of estradiol or at least 625 µg of estrone sulfate, compared to 10 µg of the synthetic estrogen ethinylestradiol. Similarly, 200,000 µg of progesterone is required compared to 150–1000 µg of most synthetic gestogens. The need for such elevated doses serves as an indicative factor of the low bioavailability within this category of sex steroids [70]. In addition, the described products (**3**–**8**) should not be inhibitors of monooxygenases necessary for the proper functioning of the human body. Based on the predicted pharmacokinetic and pharmacodynamic data (Table 3), 11α-hydroxypregn-1,4-diene-3,20-dione (**3**), 6β,11α-dihydroxypregn-1,4-diene-3,20-dione (**4**), 6β-hydroxypregn-1,4-diene-3,11,20-trione (**5**), 6β,17α-dihydroxypregn-1,4-diene-3,20-dione (**6**), 6β,17β-dihydroxyandrost-1,4-diene-3-one (**7**), and 12β,17α-dihydroxypregn-1,4-diene-3,20-dione (**8**) can be considered as potential drugs [64].

Based on our in silico studies using the platform Way2Drug PASS Online (http://www.way2drug.com/PASSOnline/predict.php, accessed on 30 May 2023), information was obtained that the described substrates (**1**, **2**) and products (**3**–**8**) should be, with very high probability, the substrates for many cytochrome P-450 monooxygenases (Table 4), which should result in their rapid metabolism within the human body. Additionally, all described compounds should be, with high probability, the inducers of CYP3A monooxygenases. The CYP3A subfamily of cytochrome P450 (CYP) in humans predominantly consists of CYP3A4, 3A5, and 3A7 (Supplementary Materials: Figures S2, S9, S16, S23, S30, S37, S39 and S46). CYP3A forms play a crucial role in the metabolism of a surprisingly large number of structurally diverse xenobiotics and endobiotics [71,72]. The pregnane X receptor (PXR), efficiently activated by natural C21 steroids (pregnanes), has been implicated in regulating *CYP3A* genes in response to xenobiotics [73]. PXR can bind to and activate transcription from specific response elements present in the *CYP3A* gene promoter across multiple species [74,75].

Progesterone (**1**), based on the predictions made (Table 5), should be an effective inhibitor of CYP17. However, the resulting compounds (**2**–**8**) should be slightly weaker inhibitors of this enzyme than progesterone (**1**). The inhibition of the key enzyme catalyzing the biosynthesis of androgens from the pregnane precursors, specifically the 17-hydroxy/17,20-lyase (referred to as CYP17), could effectively prevent androgen production from all sources. Consequently, the complete suppression of androgen production through the potent CYP17 inhibitors may offer an effective treatment strategy for prostate cancer patients [76,77]. However, it is worth noting that an increased risk of advanced breast cancer is associated with a common allele of the cytochrome P450cl7α gene (CYP17), denoted as A2. The CYP17 genotype demonstrates an association with serum hormone levels among 83 young, nulliparous women. Women heterozygous and homozygous for the CYP17 A2 allele exhibit 7% and 28% higher serum estradiol levels, respectively, and 24% and 30% higher progesterone levels around day 22 of the menstrual cycle compared to women with the A1/A1 genotype [78]. It is important to acknowledge that CYP17A1 serves as an essential human steroidogenic enzyme, facilitating two sequential reactions leading to the formation of androstenedione from progesterone and dehydroepiandrosterone from pregnenolone. The second reaction involves C17–C20 bond scission, which is significantly influenced by the presence of cytochrome b5. Moreover, this reaction displays a more pronounced acceleration, particularly when 17α-hydroxyprogesterone is a substrate [79].

The described compounds (**2**–**8**) should very likely be the substrates for UDP-glucuronosyltransferases (UGTs) (Table 5). The UGT1A enzymes are involved in the metabolism and detoxification of numerous therapeutic drugs and other xenobiotics [80,81]. The UGT1A and UGT2B subfamilies of enzymes are responsible for glucuronidation, which is a major Phase II metabolic pathway that conjugates numerous drugs [82]. They should also, with a high probability, be the substrates for sulfotransferase (Table 6). Sulfotransferases (SULTs) are enzymes that catalyze the transfer of a sulfonate group from the universal sulfate donor to an acceptor group of numerous substrates, which facilitates sulfated compound transport into cells. This process is important for the metabolism and regulation of steroids in various physiological processes [83,84].

Based on our prediction studies using the Way2Drug PASS Online platform (http://www.way2drug.com/PASSOnline/predict.php, accessed on 30 May 2023), information was obtained that the described substrates (**1**, **2**) and products (**3**–**8**) with very high probability should be inhibitors of testosterone 17β-dehydrogenase (NADP^+^), known also as 17β-HSD3: an enzyme playing an important role in the regulation of steroid hormones, such as estrogens and androgens, by catalyzing the reduction of 17-ketosteroids or the oxidation of 17β-hydroxysteroids using NAD(P)H or NAD(P)^+^ as a cofactor [85]. Most of the obtained products should also, with a high probability, find potential uses as androgen antagonists, and prostate as well as menopausal disorders treatments. They should also demonstrate immunosuppressive, erythropoiesis-stimulating, and anti-inflammatory properties (Table 6).

## 3. Materials and Methods

### 3.1. Biotransformation Procedure

Erlenmeyer flasks (300 mL), each containing 100 mL of the sterile cultivation medium (3% glucose, 1% peptone) (POCH, Gliwice, Poland), were aseptically inoculated with a suspension of each entomopathogenic strain and then incubated for 3 days at 24 °C on a rotary shaker. After this incubation period, 10 mg of the substrate (pregn-1,4-diene-3,20-dione (**2**) dissolved in 1 mL of dimethyl sulfoxide (DMSO) (Merck, Darmstadt, Germany)) was introduced into each flask. Sampling was conducted on the 1st, 3rd, 7th, and 10th days of the cultivation process, with the experiment being repeated in triplicate for validation. Subsequently, all reaction products were extracted using ethyl acetate, and the resulting extracts were subjected to drying with anhydrous MgSO_4_ (Merck, Darmstadt, Germany), followed by concentration under vacuum. The concentrated samples were then analyzed using TLC (Merck, Darmstadt, Germany), GC, and HPLC (Thermo Scientific, Waltham, MA, USA) methods, without employing sample derivatization. Quantitative analyses of the reaction mixtures were performed using GC and HPLC (Thermo Scientific, Waltham, MA, USA). This comprehensive approach ensured the accurate assessment of the biotransformation process in the tested entomopathogenic strains.

### 3.2. Procedure for Obtaining the Pregn-1,4-diene-3,20-dione (Δ^1^-Progesterone) (***2***)

The pregn-1,4-diene-3,20-dione (Δ^1^-progesterone) (**2**) was obtained from progesterone (**1**) (purchased from Sigma-Aldrich, St. Louis, MO, USA) by carrying out an enzymatic reaction on an increased scale using cholest-4-en-3-one Δ^1^-dehydrogenase (AcmB) from *Sterolibacterium denitrificans* that was recombinantly produced in *E. coli* BL21(DE3)Magic, as described in [32]. The reaction was conducted on a 100 mL scale in 100 mM K_2_HPO_4_/KH_2_PO_4_ pH 8.0 that contained 1.4 μM of AcmB, 8% (*w*/*v*) of (2-hydroxypropyl)-β-cyclodextrin as a steroid solubilizer, and 15 mM phenazine methosulfate as an electron acceptor. The steroid substrate, dissolved in 2-methoxyethanol, was added to the reaction mixture yielding the final concentrations of 3.5 g/L (11.1 mM) and 4% (*v*/*v*) 2-methoxyethanol. The reaction was conducted in aerobic conditions at 30 °C with stirring (220 rpm) for 5.5 h until a 100% conversion was obtained. The reaction was followed with HPLC according to the procedure described in [32]. The pregn-1,4-diene-3,20-dione (Δ^1^-progesterone) (**2**) was separated using 2000 µm preparative TLC silica gel plates (Anatech, Gehrden, Germany). The mobile phase contained a mixture of hexane and acetone in a 3:1 (*v*/*v*) ratio. Separation products were scraped out and extracted twice using ethyl acetate, the solvent was evaporated off and obtained compound **2** (isolated yield > 95%), then the products were analyzed using TLC, GC, HPLC, and NMR spectroscopy (^1^H NMR, ^13^C NMR, COSY, HMBC and HSQC) analysis.

### 3.3. Product Samples

For the scale-up process, larger Erlenmeyer flasks (2000 mL) were employed, each filled with 500 mL of the identical cultivation medium (3% glucose, 1% peptone; POCH, Gliwice, Poland). Inoculation was conducted following the previously described procedure. Three days post-inoculation, 100 mg of the substrate was dissolved in 2 mL of dimethyl sulfoxide (DMSO) (Merck, Darmstadt, Germany) and introduced into each flask. Sampling was performed exclusively on the 10th day of the cultivation process. The products were extracted thrice using ethyl acetate to ensure comprehensive recovery and analysis.

### 3.4. Analysis

Initial assessments were conducted utilizing thin-layer chromatography (TLC) plates (SiO_2_, DC Alufolien Kieselgel 60 F254 (0.2 mm thick), Merck, Darmstadt, Germany). The mobile phase consisted of a hexane and acetone mixture in a 2:1 (*v*/*v*) ratio. Visualization of the plates was performed under a UV lamp (254 and 365 nm). For the scale-up biotransformation products, 1000 µm preparative TLC silica gel plates (Anatech, Gehrden, Germany) were employed. The mobile phase comprised a hexane and acetone mixture in a 2:1 (*v*/*v*) ratio. Following separation, the products were scraped off and subjected to double extraction using ethyl acetate. The solvent was subsequently evaporated, and the remaining residue was analyzed using a combination of TLC, GC, HPLC, and various NMR spectroscopy techniques (^1^H NMR, ^13^C NMR, COSY, HMBC, and HSQC) (Bruker, Billerica, MA, USA) (Table 1 and Table 7).

### 3.5. Microorganisms

The microorganisms *Beauveria bassiana* KCh J1.5, *B. caledonica* KCh J3.3, *Isaria farinosa* KCh KW 1.1 and *I. fumosorosea* KCh J2, and *Metarhizium robertsii* MU4 and *Isaria tenuipes* MU35 were obtained from the collection of the Department of Food Chemistry and Biocatalysts, at the Wrocław University of Environmental and Life Sciences (Wrocław, Poland) and the collection of the Institute of Plant Genetics, at the Polish Academy of Sciences (Poznań, Poland). Isolation and identification procedures were described in our previous papers [36,52,86].

### 3.6. GC and HPLC

GC analysis was performed using a Hewlett Packard 5890A Series II GC instrument (FID, carrier gas H_2_ at flow rate of 2 mL/min, Hewlett-Packard Company, Wilmington, DE, USA) with a DB-5MS column of cross-linked phenyl-methylsiloxane, at a 30 m × 0.32 mm × 0.25 μm film thickness. The following program was used in the GC analysis: 220 °C/1 min, gradient 4 °C/min to 280 °C and 30 °C/min to 300 °C/3 min; injector and detector temperatures were 300 °C. HPLC analysis was also performed to confirm the compositions of the reaction mixtures. HPLC analyses were performed on an DIONEX UltiMate 3000 chromatograph from Thermo Fisher Scientific (Olten, Switzerland) equipped with a Corona charged aerosol detector (CAD) from ESA Biosciences (Chelmsford, MA, USA), using an Agilent Zorbax Eclipse XDB-C18 (4.6 mm × 250 mm, 5 µm, Agilent, Santa Clara, CA, USA) and the following elution program: gradient elution from 0 to 3 min (85% A → 50% A) at a flow rate of 1.0 mL/min; gradient elution from 3 to 7 min (50% A → 40% A) at a flow rate of 1.5 mL/min; gradient elution from 7 to 10 min (60% A → 0% A) at a flow rate of 1.5 mL/min; isocratic elution from 10 to 13 min (% A) at a flow rate of 2.0 mL/min; gradient elution from 13 to 16 min (0% A → 85% A) at a flow rate of 1.5 mL/min; isocratic elution from 16 to 17 min (85% A). Solvent A consisted of 0.1% HCOOH in water, and solvent B consisted of 0.1% HCOOH in CH_3_CN. The column temperature was 28 °C. The injection volume was 10μL. The percentages of the components from the reaction mixtures obtained by HPLC are in high agreement with those obtained by GC (Retention times [min] of the tested compounds are presented in Table 8).

### 3.7. Pharmacokinetics, Drug Nature, Biological Activity Prediction

The predictions regarding the pharmacokinetic and physicochemical properties, medicinal chemistry suitability, and potential biological activity of the steroid derivatives based on their structural formulae were computed using SwissADME (accessible online: http://www.swissadme.ch, accessed on 30 May 2023) and the Way2Drug PASS Online, along with accompanying services (available online: http://www.way2drug.com/PASSOnline, accessed on 30 May 2023). The structural formulae of the molecules were constructed using ACD Chemsketch 2021.2.0 and saved in mol format. Subsequently, these files were imported into both services for analysis. In PASS Online, biological activity types are presented as the probability to be revealed (Pa) and not to be revealed (Pi), with independent values within the range from 0 to 1.

## 4. Conclusions

The entomopathogenic filamentous fungi strains utilized as biocatalysts, namely *Beauveria bassiana* KCh J1.5, *Beauveria caledonica* KCh J3.3, *Isaria fumosorosea* KCh J2, *Isaria farinosa* KCh KW1.1, *Isaria tenuipes* MU35, and *Metarhizium robertsii* MU4, have demonstrated effective transformation capabilities on pregn-1,4-diene-3,20-dione (Δ^1^-progesterone) (**2**). The substrate (**2**) was derived from progesterone (**1**) through an enzymatic reaction conducted on an increased scale. Cholest-4-en-3-one Δ^1^-dehydrogenase (AcmB) from *Sterolibacterium denitrificans*, recombinantly produced in *E. coli* BL21(DE3)Magic, was employed in the enzymatic reaction.

The obtained products are more polar than substrate (**2**), and the composition of the product mixture in the cultures of most of the tested biocatalysts changes during incubation, which indicates the involvement of at least several enzymes in the transformation of Δ^1^-progesterone (**2**). The introduction of a double bond (compound **2**) resulted in a change in the course of the biotransformation compared to the previously described progesterone (**1**) biotransformations.

The presented results indicate that, in general, the tested entomopathogenic biocatalysts produce a multitude of enzymes capable of transforming Δ^1^-progesterone, among others, into 11α-, 6β-, 6β,11α-, 6oxo,11α-, and 12β,17α-hydroxy derivatives.

The 17α-hydroxylation reaction of progesterone and its derivatives is one of the most desirable transformations of steroid compounds leading to valuable products. Based on the experiments carried out, it has been shown that two strains, *Isaria fumosorosea* KCh J2, *Isaria farinosa* KCh KW1.1, are capable of carrying out this particular reaction.

It is worth emphasizing that among the bioconversion products, we discovered a derivative without an acyl side chain.

We have demonstrated that all the products have significantly lower lipophilicity and significantly higher water solubility than Δ^1^-progesterone (**2**), which is caused by the introduction of polar -OH groups during the transformation. Based on our prediction studies using the Way2Drug PASS Online platform, information was obtained that the described substrates (**1**, **2**) and products (**3**–**8**) with very high probability should be inhibitors of testosterone 17β-dehydrogenase. Most of the obtained products with high probability should also find potential uses as androgen antagonists, and prostate as well as menopausal disorders treatments. They should also demonstrate immunosuppressive, erythropoiesis-stimulating, and anti-inflammatory properties.

Moreover, investigations into the properties of steroids, including their Δ^1^-progesterone derivatives, are ongoing, and there is a continuous search for efficient methods to obtain such compounds. The methodologies outlined in this publication enable the production of substantial quantities of Δ^1^-progesterones in an effective and cost-effective manner, aligning with the principles of “green chemistry”.

## Figures and Tables

**Table 1:** Composition of the reaction mixture [%] after 1, 3, 7, and 10 days, obtained during biotransformation of pregn-1,4-diene-3,20-dione (Δ^1^-progesterone) (**2**) by GC.

Strain	Compounds	Time of Biotransformation [Days]			
		1	3	7	10
*Metarhizium robertsii* MU4	**2**	0	0	0	0
	**3**	100	100	96	80
	**4**	0	0	4	6
*Beauveria bassiana* KCh J1.5	**2**	0	0	0	0
	**3**	95	50	0	0
	**4**	5	39	70	60
	**5**	0	11	28	36
	unidentified compounds	0	0	2	4
*Beauveria caledonica* KCh J3.3	**2**	29	0	0	0
	**3**	53	74	54	12
	**4**	3	6	18	24
	unidentified compounds	15	20	28	64
*Isaria fumosorosea* KCh J2	**2**	0	0	0	0
	**3**	4	0	0	0
	**4**	21	19	22	23
	**6**	17	12	3	0
	**7**	28	32	54	57
	unidentified compounds	20	19	18	20
*Isaria tenuipes* MU35	**2**	0	0	0	0
	unidentified compounds	100	100	100	100
*Isaria farinosa* KCh KW1.1	**2**	0	0	0	0
	**3**	4	4	3	0
	**4**	4	3	3	3
	**8**	33	42	52	56
	unidentified compounds	59	51	42	41

Data are expressed as the mean of three independent experiments. Standard errors were in the range of 0–5.

**Table 2:** Summary of signals visible in the ^13^C NMR spectrum of the substrates and the biotransformation products.

Atom Number	Products						
	2 (CDCl_3_)	3 (CDCl_3_)	4 (DMSO)	5 (CDCl_3_)	6 (CDCl_3_)	7 (CDCl_3_)	8 (DMSO)
1	155.73	159.28	161.04	156.76	157.46	157.46	156.08
2	127.76	125.15	123.34	126.90	126.97	126.88	126.85
3	186.45	187.05	185.92	186.82	186.82	186.81	184.96
4	124.09	124.59	124.92	127.08	126.12	126.05	123.16
5	169.00	168.50	166.88	163.79	165.84	166.09	169.24
6	32.91	33.21	72.10	73.32	74.00	77.40	29.58
7	33.65	33.60	40.04	40.35	40.20	39.76	32.99
8	35.61	34.43	28.37	31.42	30.30	32.11	34.01
9	52.33	60.59	58.94	59.94	51.51	52.21	50.41
10	44.21	44.07	43.84	42.78	43.69	43.88	42.98
11	22.94	67.97	66.37	207.91	22.87	22.17	31.98
12	38.65	49.83	48.67	56.45	30.32	36.49	68.04
13	43.62	44.18	43.51	47.09	48.71	43.29	53.21
14	55.73	54.96	54.15	54.76	49.69	50.29	48.51
15	24.68	24.51	24.05	24.22	24.31	23.68	23.20
16	22.96	23.15	22.38	23.60	33.67	30.60	34.58
17	63.51	62.95	62.14	62.02	89.79	81.74	88.55
18	13.57	14.51	14.12	14.56	15.79	11.38	9.04
19	18.83	18.71	20.11	20.51	20.62	20.58	18.41
20	209.29	208.90	208.34	207.95	211.83	-	213.45
21	31.59	31.50	31.05	31.31	28.16	-	27.51

**Table 3:** Pharmacokinetic and pharmacodynamic data compiled from the online tool SwissADME.

Activity/Parameter	1	2	3	4	5	6	7	8
Lipophilicity (Consensus Log Po/w)	4.02	3.91	2.99	2.16	2.22	2.33	2.48	2.38
Water Solubility [mg/mL]	0.013	0.038	0.260	1.780	0.358	0.633	1.890	0.633
Water Solubility [mol/L]	4.15 × 10^−5^	1.22 × 10^−4^	7.93 × 10^−4^	5.18 × 10^−3^	1.04 × 10^−3^	1.84 × 10^−3^	6.25 × 10^−3^	1.84 × 10^−3^
Gastrointestinal absorption	High	High	High	High	High	High	High	High
BBB permeant	Yes	Yes	Yes	Yes	Yes	Yes	Yes	Yes
P-glycoprotein substrate	No	No	Yes	Yes	Yes	Yes	Yes	Yes
CYP1A2 inhibitor	No	No	No	No	No	No	No	No
CYP2C19 inhibitor	Yes	Yes	No	No	No	No	No	No
CYP2C9 inhibitor	Yes	Yes	No	No	No	No	No	No
CYP2D6 inhibitor	No	No	No	No	No	No	No	No
CYP3A4 inhibitor	No	No	No	No	No	No	No	No
Log Kp (skin permeation) [cm/s]	−5.47	−5.31	−6.48	−7.29	−7.47	−7.17	−6.55	−7.18
TPSA [Å^2^]	34.14	34.14	54.37	74.60	71.44	74.60	57.53	74.60

TPSA—Topological Polar Surface Area, the surface sum over all polar atoms of the molecule (oxygen, nitrogen, sulfur and phosphorus), including their attached hydrogen atoms.

**Table 4:** Summary of the most probable metabolization of progesterone (**1**), Δ^1^-progesterone (**2**), and its metabolites (**3**–**8**) by monooxygenases predicted using the PASS Online tool.

Compound	Pa	Pi	CYP	Pa	Pi	CYP	Pa	Pi	CYP	Pa	Pi	CYP	Pa	Pi	CYP
**1**	0.950	0.004	2C12	0.945	0.001	2B5	0.946	0.002	2J	0.940	0.002	2J2	0.919	0.004	2C
**2**	0.943	0.001	2A1	0.940	0.004	2C	0.932	0.002	3A1	0.917	0.002	2B5	0.907	0.004	2C9
**3**	0.971	0.003	2C	0.952	0.002	3A1	0.944	0.003	2C9	0.922	0.002	3A2	0.923	0.004	3A4
**4**	0.952	0.004	2C	0.937	0.004	3A4	0.929	0.004	2C9	0.921	0.004	3A	0.919	0.003	3A1
**5**	0.960	0.003	2B	0.940	0.004	3A4	0.924	0.004	3A	0.880	0.004	2B6	0.834	0.008	2C
**6**	0.967	0.003	2C	0.956	0.003	2C9	0.942	0.003	3A4	0.926	0.004	3A	0.890	0.004	2B
**7**	0.965	0.003	2C12	0.951	0.002	2J	0.949	0.002	2J2	0.937	0.004	2B	0.930	0.003	2B6
**8**	0.969	0.003	2C	0.954	0.003	2C9	0.932	0.006	2C12	0.913	0.005	3A4	0.911	0.003	3A1

Pa—probable activity; Pi—probable inactivity. Values range from 0 to 1, where 1 represents a 100% probability of Pa or Pi, and 0 represents a 0% probability of Pa or Pi.

**Table 5:** Interaction with key enzymes predictions for progesterone (**1**), Δ^1^-progesterone (**2**), and its metabolites (**3**–**8**) using the PASS Online tool.

Activity		Number of Compound							
		1	2	3	4	5	6	7	8
UGT1A substrate	Pa	0.850	0.807	0.927	0.968	0.972	0.831	0.918	0.791
	Pi	0.004	0.004	0.003	0.002	0.002	0.004	0.003	0.005
UGT2B substrate	Pa	0.794	0.751	0.911	0.935	0.935	0.922	0.823	0.854
	Pi	0.003	0.003	0.002	0.001	0.001	0.002	0.002	0.002
UGT1A4 substrate	Pa	0.853	0.771	0.848	0.806	0.810	0.712	0.760	0.753
	Pi	0.002	0.003	0.002	0.003	0.002	0.005	0.004	0.004
UGT1A6 substrate	Pa	0.431	0.460	0.613	0.834	0.593	0.727	0.959	0.572
	Pi	0.020	0.017	0.008	0.004	0.009	0.005	0.002	0.010
UGT1A9 substrate	Pa	0.633	0.667	0.712	0.765	0.696	0.827	0.947	0.805
	Pi	0.011	0.009	0.007	0.005	0.008	0.004	0.002	0.004
CYP3A4 inducer	Pa	0.870	0.901	0.934	0.912	0.912	0.909	0.864	0.923
	Pi	0.004	0.003	0.002	0.002	0.002	0.002	0.004	0.002
CYP3A inducer	Pa	0.850	0.896	0.937	0.912	0.911	0.908	0.844	0.925
	Pi	0.004	0.003	0.002	0.002	0.002	0.002	0.004	0.002
CYP17 inhibitor	Pa	0.911	0.857	0.811	0.759	0.759	0.631	0.748	0.707
	Pi	0.001	0.002	0.002	0.003	0.003	0.007	0.003	0.004

Pa—probable activity; Pi—probable inactivity. Values range from 0 to 1, where 1 represents a 100% probability of Pa or Pi, and 0 represents a 0% probability of Pa or Pi.

**Table 6:** Biological activity predictions for progesterone (**1**), Δ^1^-progesterone (**2**), and its metabolites (**3**–**8**) using the PASS Online tool.

Activity		Number of Compound							
		1	2	3	4	5	6	7	8
Testosterone 17β-dehydrogenase (NADP^+^) inhibitor	Pa	0.972	0.935	0.931	0.879	0.879	0.914	0.976	0.946
	Pi	0.002	0.004	0.004	0.009	0.009	0.005	0.001	0.003
Indanol dehydrogenase inhibitor	Pa	0.780	0.963	0.976	0.959	0.915	0.955	0.831	0.953
	Pi	0.003	0.001	0.000	0.001	0.002	0.001	0.003	0.001
Antiinflammatory	Pa	0.671	0.859	0.918	0.903	0.840	0.903	0.809	0.959
	Pi	0.020	0.005	0.004	0.004	0.005	0.004	0.006	0.003
Antiinflammatory, ophthalmic	Pa	0.795	0.839	0.912	0.823	0.605	0.626	0.497	0.638
	Pi	0.001	0.001	0.001	0.001	0.003	0.002	0.004	0.002
Prostaglandin-E2 9-reductase inhibitor	Pa	0.915	0.942	0.971	0.946	0.873	0.915	0.893	0.934
	Pi	0.004	0.003	0.002	0.003	0.006	0.004	0.005	0.003
UDP-glucuronosyltransferase substrate	Pa	0.858	0.810	0.924	0.957	0.957	0.943	0.884	0.883
	Pi	0.004	0.006	0.003	0.002	0.002	0.002	0.003	0.003
Anesthetic general	Pa	0.925	0.825	0.771	0.636	0.895	0.374	0.466	0.540
	Pi	0.003	0.004	0.005	0.010	0.004	0.047	0.026	0.016
Membrane permeability inhibitor	Pa	0.911	0.839	0.854	0.850	0.804	0.674	0.725	0.669
	Pi	0.003	0.005	0.005	0.005	0.009	0.050	0.029	0.053
Erythropoiesis stimulant	Pa	0.800	0.781	0.754	0.773	0.871	0.642	0.721	0.599
	Pi	0.002	0.003	0.003	0.003	0.001	0.008	0.004	0.013
Androgen antagonist	Pa	0.799	0.741	0.877	0.619	0.377	0.631	0.441	0.752
	Pi	0.003	0.003	0.003	0.004	0.007	0.004	0.006	0.003
Immunosuppressant	Pa	0.682	0.727	0.790	0.787	0.771	0.799	0.740	0.772
	Pi	0.019	0.014	0.006	0.006	0.009	0.005	0.012	0.008
Prostate disorders treatment	Pa	0.845	0.848	0.770	0.741	0.757	0.689	0.716	0.677
	Pi	0.003	0.003	0.004	0.005	0.004	0.007	0.005	0.008
Sulfotransferase substrate	Pa	0.844	0.686	0.876	0.860	0.561	0.857	0.855	0.852
	Pi	0.003	0.005	0.003	0.003	0.007	0.003	0.003	0.003
Menopausal disorders treatment	Pa	0.691	0.760	0.712	0.639	0.639	0.764	0.692	0.818
	Pi	0.003	0.003	0.003	0.005	0.005	0.003	0.003	0.002

Pa—probable activity; Pi—probable inactivity. Values range from 0 to 1, where 1 represents a 100% probability of Pa or Pi, and 0 represents a 0% probability of Pa or Pi.

**Table 7:** ^1^H NMR (600 MHz) chemical shifts δ (ppm) and coupling constants *J* (Hz) of substrate (**2**) and products of its biotransformation (**3**–**8**).

Compound	Solvent	Chemical Shift of Characteristic Signals δ:
**2**	CDCl_3_	0.69 (s, 3H, 18-H); 1.23 (s, 3H, 19-H); 2.12 (s, 3H, 21-H); 6.07 (t, 1H, *J* = 1.7 Hz, 4-H); 6.24 (dd, 1H, *J* = 10.1, 1.7 Hz, 2-H); 7.05 (d, 1H, *J* = 10.1 Hz, 1-H).
**3**	CDCl_3_	0.71 (s, 3H, 18-H); 1.31 (s, 3H, 19-H); 2.13 (s, 3H, 21-H); 4.06 (td, 1H, *J* = 9.7, 4.2 Hz, H-11β); 6.10 (t, 1H, *J* = 1.6 Hz, 4-H); 6.16 (dd, 1H, *J* = 10.1, 1.6 Hz, 2-H); 7.77 (d, 1H, *J* = 10.1 Hz, 1-H).
**4**	DMSO	0.57 (s, 3H, 18-H); 1.37 (s, 3H, 19-H); 2.03 (s, 3H, 21-H); 3.89 (tdd, *J* = 10.8, 6.2, 5.0 Hz, H-11β); 4.31 (q, *J* = 2.5 Hz, H-6α); 5.93 (dd, 1H, *J* = 10.3, 2.0 Hz, 2-H); 6.00 (d, 1H, *J* = 2.0 Hz, 4-H); 7.80 (d, 1H, *J* = 10.3 Hz, 1-H).
**5**	CDCl_3_	0.69 (s, 3H, 18-H); 1.64 (s, 3H, 19-H); 2.10 (s, 3H, 21-H); 4.55 (br s, 1H, H-6α); 6.16 (s, 1H, 4-H); 6.19 (d, 1H, *J* = 10.2 Hz, 2-H); 7.71 (d, 1H, *J* = 10.2 Hz, 1-H).
**6**	CDCl_3_	0.82 (s, 3H, 18-H); 1.43 (s, 3H, 19-H); 2.28 (s, 3H, 21-H); 4.55 (d, 1H, *J* = 2.9 Hz, H-6α); 6.16 (d, 1H, *J* = 2.0 Hz, 4-H); 6.22 (dd, 1H, *J* = 10.1, 2.0 Hz, 2-H); 7.05 (d, 1H, *J* = 10.1 Hz, 1-H).
**7**	CDCl_3_	0.84 (s, 3H, 18-H); 1.44 (s, 3H, 19-H); 3.65 (t, 1H, *J* = 8.5 Hz, H-17α); 4.54 (d, 1H, *J* = 2.7 Hz, H-6α); 6.16 (d, 1H, *J* = 2.1 Hz, 4-H); 6.22 (dd, 1H, *J* = 10.1, 2.1 Hz, 2-H); 7.06 (d, 1H, *J* = 10.1 Hz, 1-H).
**8**	DMSO	0.58 (s, 3H, 18-H); 1.18 (s, 3H, 19-H); 2.26 (s, 3H, 21-H); 3.82–3.90 (m, 1H, H-12α); 4.55 (br s, 1H, H-6α); 5.98 (s, 1H, 4-H); 6.13 (d, 1H, *J* = 10.1, 1.9 Hz, 2-H); 7.13 (d, 1H, *J* = 10.1 Hz, 1-H).

**Table 8:** Retention times [min] of the tested compounds.

Number of Compound	2	3	4	5	6	7	8
GC	7.267	10.202	12.458	10.599	11.683	10.009	11.536
HPLC	10.123	6.977	5.485	6.157	5.977	5.567	6.097

## Data Availability

Samples of compounds **1**–**8** are available from the authors.

## Supplementary Materials

The supporting information can be downloaded at: https://www.mdpi.com/article/10.3390/ijms25010508/s1.
