# Supplementary material for: Biotransformation of Δ1-Progesterone Using Selected Entomopathogenic Filamentous Fungi and Prediction of Its Products’ Bioactivity
Source: Int J Mol Sci. 2023 Dec 29;25(1):508. doi: 10.3390/ijms25010508 (PMC10779271; doi:10.3390/ijms25010508)
Supplement: Supplementary file 1 [file ijms-25-00508-s001.zip › ijms-2773596-supplementary.pdf]

Supplementary Data

Biotransformation of Δ^1-progesterone by selected entomopathogenic filamentous fungi and prediction of its products' bioactivity

Anna Panek[1]*, Patrycja Wójcik[2], Alina Świzdor[1], Maciej Szaleniec[2] and Tomasz Janeczko[1]*

[1]Department of Food Chemistry and Biocatalysis, Wrocław University of Environmental and Life Sciences, Norwida 25, 50-375 Wrocław, Poland; alina.swizdor@upwr.edu.pl (A.S.)
[2]Jerzy Haber Institute of Catalysis and Surface Chemistry, Polish Academy of Sciences, Niezapominajek 8, 30-239 Krakow, Poland; patrycwoj@gmail.com (P.W.); maciej.szaleniec@ikifp.edu.pl (M.S.)
***Correspondence: anna.panek@upwr.edu.pl (A.P.); janeczko13@interia.pl (T.J.); Tel.: +48-713-205-195

Contents:

Figure S1. Predicted Boiled-Egg plot from swissADME online web tool for progesterone (**1**)

Figure S2. progesterone (**1**) physicochemical and ADME parameters prediction using the SwissADME modelling

Figure S3. ^{1}H NMR spectra of pregn-1,4-diene-3,20-dione (Δ^1-progesterone) (**2**) ($CDCl_3$, 600 MHz)

Figure S4. ^{13}C NMR spectra of pregn-1,4-diene-3,20-dione (Δ^1-progesterone) (**2**) ($CDCl_3$, 151 MHz)

Figure S5. COSY spectrum of pregn-1,4-diene-3,20-dione (Δ^1-progesterone) (**2**) ($CDCl_3$, 600 MHz)

Figure S6. HSQC spectrum of pregn-1,4-diene-3,20-dione (Δ^1-progesterone) (**2**) ($CDCl_3$, 600/151 MHz)

Figure S7. HMBC spectrum of pregn-1,4-diene-3,20-dione (Δ^1-progesterone) (**2**) ($CDCl_3$, 600/151 MHz)

Figure S8. Predicted Boiled-Egg plot from swissADME online web tool for pregn-1,4-diene-3,20-dione (Δ^1-progesterone) (**2**)

Figure S9. pregn-1,4-diene-3,20-dione (Δ^1-progesterone) (**2**) physicochemical and ADME parameters prediction using the SwissADME modelling

Figure S10. ^{1}H NMR spectra of 11α-hydroxypregn-1,4-diene-3,20-dione (**3**) ($CDCl_3$, 600 MHz)

Figure S11. ^{13}C NMR spectra of 11α-hydroxypregn-1,4-diene-3,20-dione (**3**) ($CDCl_3$, 151 MHz)

Figure S12. COSY spectrum of 11α-hydroxypregn-1,4-diene-3,20-dione (**3**) ($CDCl_3$, 600 MHz)

Figure S13. HSQC spectrum of 11α-hydroxypregn-1,4-diene-3,20-dione (**3**) ($CDCl_3$, 600/151 MHz)

Figure S14. HMBC spectrum of 11α-hydroxypregn-1,4-diene-3,20-dione (**3**) ($CDCl_3$, 600/151 MHz)

Figure S15. Predicted Boiled-Egg plot from swissADME online web tool for 11α-hydroxypregn-1,4-diene-3,20-dione (**3**)

Figure S16. 11α-hydroxypregn-1,4-diene-3,20-dione (**3**) physicochemical and ADME parameters prediction using the SwissADME modelling

Figure S17. ^{1}H NMR spectra of 6β,11α-dihydroxypregn-1,4-diene-3,20-dione (**4**) (DMSO-d_6, 600 MHz)

Figure S18. ^{13}C NMR spectra of 6β,11α-dihydroxypregn-1,4-diene-3,20-dione (**4**) (DMSO-d_6, 151 MHz)

Figure S19. COSY spectrum of 6β,11α-dihydroxypregn-1,4-diene-3,20-dione (**4**) (DMSO-d_6, 600 MHz)

Figure S20. HSQC spectrum of 6β,11α-dihydroxypregn-1,4-diene-3,20-dione (**4**) (DMSO-d_6, 600/151 MHz)

Figure S21. HMBC spectrum of 6β,11α-dihydroxypregn-1,4-diene-3,20-dione (**4**) (DMSO-d_6, 600/151 MHz)

Figure S22. Predicted Boiled-Egg plot from swissADME online web tool for 6β,11α-dihydroxypregn-1,4-diene-3,20-dione (**4**)

Figure S23. 6β,11α-dihydroxypregn-1,4-diene-3,20-dione (**4**) physicochemical and ADME parameters prediction using the SwissADME modelling

Figure S24. ^{1}H NMR spectra of 6β-hydroxypregn-1,4-diene-3,11,20-trione (**5**) ($CDCl_3$, 600 MHz)

Figure S25. ^{13}C NMR spectra of 6β-hydroxypregn-1,4-diene-3,11,20-trione (**5**) ($CDCl_3$, 151 MHz)

Figure S26. COSY spectrum of 6β-hydroxypregn-1,4-diene-3,11,20-trione (**5**) ($CDCl_3$, 600 MHz)

Figure S27. HSQC spectrum of 6β-hydroxypregn-1,4-diene-3,11,20-trione (**5**) ($CDCl_3$, 600/151 MHz)

Figure S28. HMBC spectrum of 6β-hydroxypregn-1,4-diene-3,11,20-trione (**5**) ($CDCl_3$, 600/151 MHz)

Figure S29. Predicted Boiled-Egg plot from swissADME online web tool for 6β-hydroxypregn-1,4-diene-3,11,20-trione (**5**)

Figure S30. 6β-hydroxypregn-1,4-diene-3,11,20-trione (**5**) physicochemical and ADME parameters prediction using the SwissADME modelling

Figure S31. ^{1}H NMR spectra of 6β,17α-dihydroxypregn-1,4-diene-3,20-dione (**6**) and 6β,17β-dihydroxyandrost-1,4-diene-3-one (**7**) ($CDCl_3$, 600 MHz)

Figure S32. ^{13}C NMR spectra of 6β,17α-dihydroxypregn-1,4-diene-3,20-dione (**6**) and 6β,17β-dihydroxyandrost-1,4-diene-3-one (**7**) ($CDCl_3$, 151 MHz)

Figure S33. COSY spectrum of 6β,17α-dihydroxypregn-1,4-diene-3,20-dione (**6**) and 6β,17β-dihydroxyandrost-1,4-diene-3-one (**7**) ($CDCl_3$, 600 MHz)

Figure S34. HSQC spectrum of 6β,17α-dihydroxypregn-1,4-diene-3,20-dione (**6**) and 6β,17β-dihydroxyandrost-1,4-diene-3-one (**7**) ($CDCl_3$, 600/151 MHz)

Figure S35. HMBC spectrum of 6β,17α-dihydroxypregn-1,4-diene-3,20-dione (**6**) and 6β,17β-dihydroxyandrost-1,4-diene-3-one (**7**) ($CDCl_3$, 600/151 MHz)

Figure S36. Predicted Boiled-Egg plot from swissADME online web tool for 6β,17α-dihydroxypregn-1,4-diene-3,20-dione (**6**)

Figure S37. 6β,17α-dihydroxypregn-1,4-diene-3,20-dione (**6**) physicochemical and ADME parameters prediction using the SwissADME modelling

Figure S38. Predicted Boiled-Egg plot from swissADME online web tool for 6β,17β-dihydroxyandrost-1,4-diene-3-one (**7**)

Figure S39. 6β,17β-dihydroxyandrost-1,4-diene-3-one (**7**) physicochemical and ADME parameters prediction using the SwissADME modelling

Figure S40. ^{1}H NMR spectra of 12β,17α-dihydroxypregn-1,4-diene-3-one (**8**) (DMSO-d_6, 600 MHz)

Figure S41. ^{13}C NMR spectra of 12β,17α-dihydroxypregn-1,4-diene-3-one (**8**) (DMSO-d_6, 151 MHz)

Figure S42. COSY spectrum of 12β,17α-dihydroxypregn-1,4-diene-3-one (**8**) (DMSO-d_6, 600 MHz)

Figure S43. HSQC spectrum of 12β,17α-dihydroxypregn-1,4-diene-3-one (**8**) (DMSO-d_6, 600/151 MHz)

Figure S44. HMBC spectrum of 12β,17α-dihydroxypregn-1,4-diene-3-one (**8**) (DMSO-d_6, 600/151 MHz)

Figure S45. Predicted Boiled-Egg plot from swissADME online web tool for 12β,17α-dihydroxypregn-1,4-diene-3-one (**8**)

Figure S46. 12β,17α-dihydroxypregn-1,4-diene-3-one (**8**) physicochemical and ADME parameters prediction using the SwissADME modelling

Figure S1. Predicted Boiled-Egg plot from swissADME online web tool for progesterone (**1**)

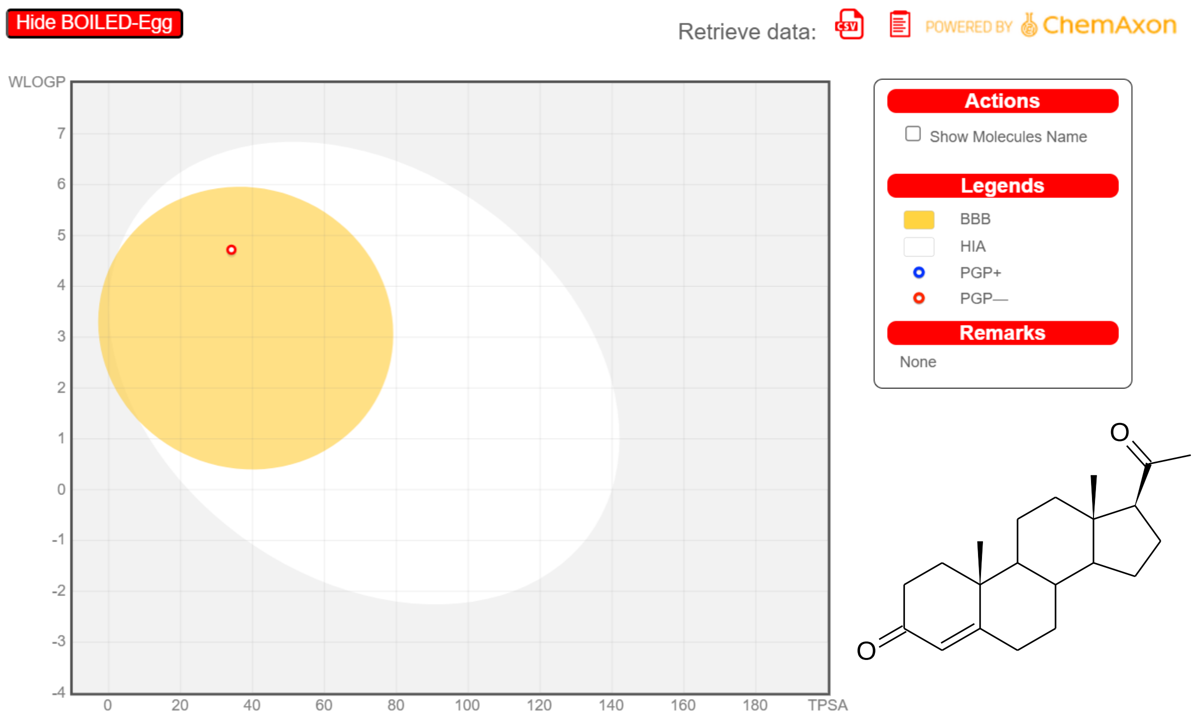

Figure S2. progesterone (**1**) physicochemical and ADME parameters prediction using the SwissADME modelling

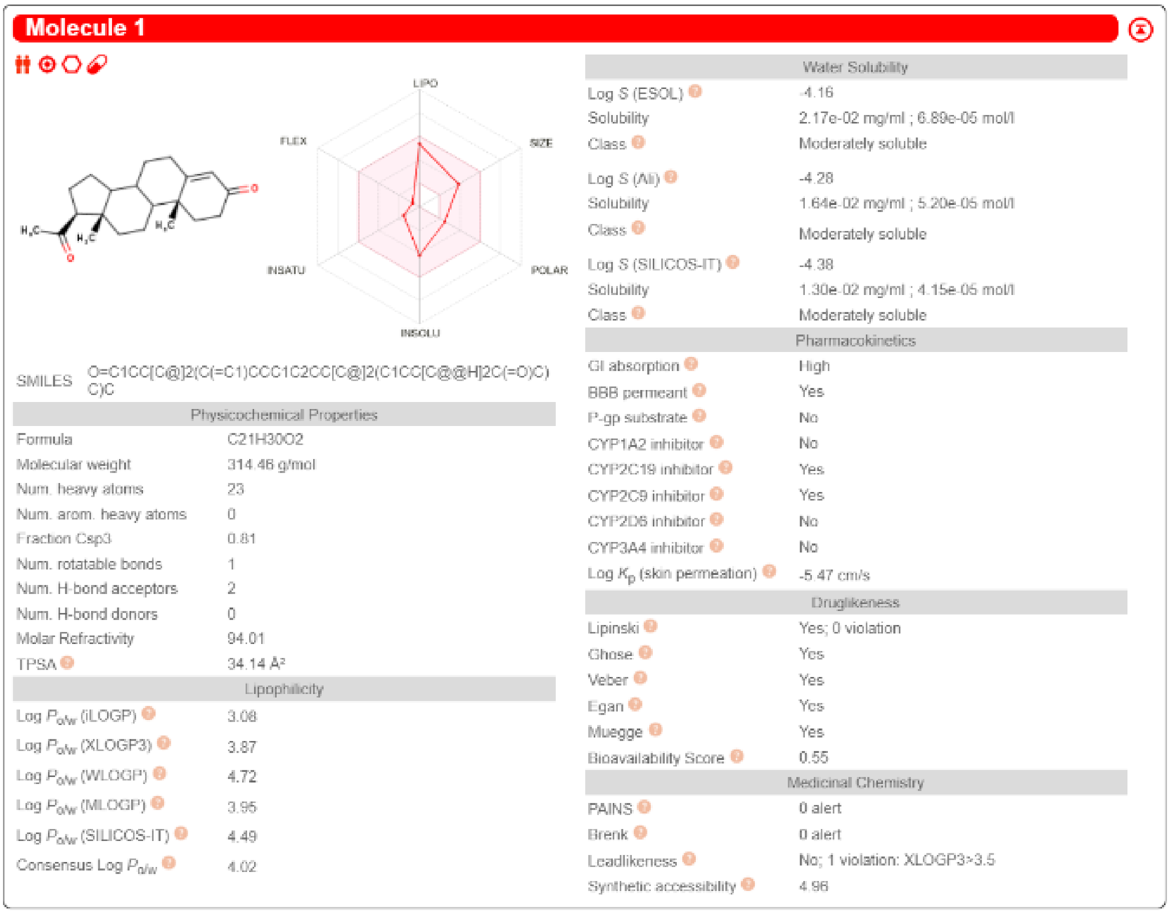

SMILES	O=C1CC[C@]2(C(=C1)CCC1C2CC[C@]2(C1CC[C@@H]2C(=O)C)C)C

Physicochemical Properties	
Formula	C21H30O2
Molecular weight	314.46 g/mol
Num. heavy atoms	23
Num. arom. heavy atoms	0
Fraction Csp3	0.81
Num. rotatable bonds	1
Num. H-bond acceptors	2
Num. H-bond donors	0
Molar Refractivity	94.01
TPSA	34.14 Å²
**Lipophilicity**	
Log $P_{o/w}$ (iLOGP)	3.08
Log $P_{o/w}$ (XLOGP3)	3.87
Log $P_{o/w}$ (WLOGP)	4.72
Log $P_{o/w}$ (MLOGP)	3.95
Log $P_{o/w}$ (SILICOS-IT)	4.49
Consensus Log $P_{o/w}$	4.02

Water Solubility	
Log S (ESOL)	-4.16
Solubility	2.17e-02 mg/ml ; 6.89e-05 mol/l
Class	Moderately soluble
Log S (Ali)	-4.28
Solubility	1.64e-02 mg/ml ; 5.20e-05 mol/l
Class	Moderately soluble
Log S (SILICOS-IT)	-4.38
Solubility	1.30e-02 mg/ml ; 4.15e-05 mol/l
Class	Moderately soluble
**Pharmacokinetics**	
GI absorption	High
BBB permeant	Yes
P-gp substrate	No
CYP1A2 inhibitor	No
CYP2C19 inhibitor	Yes
CYP2C9 inhibitor	Yes
CYP2D6 inhibitor	No
CYP3A4 inhibitor	No
Log K_p (skin permeation)	-5.47 cm/s
**Druglikeness**	
Lipinski	Yes; 0 violation
Ghose	Yes
Veber	Yes
Egan	Yes
Muegge	Yes
Bioavailability Score	0.55
**Medicinal Chemistry**	
PAINS	0 alert
Brenk	0 alert
Leadlikeness	No; 1 violation: XLOGP3>3.5
Synthetic accessibility	4.96

Figure S3. ^{1}H NMR spectra of pregn-1,4-diene-3,20-dione (Δ^{1}-progesterone) (**2**) ($CDCl_3$, 600 MHz)

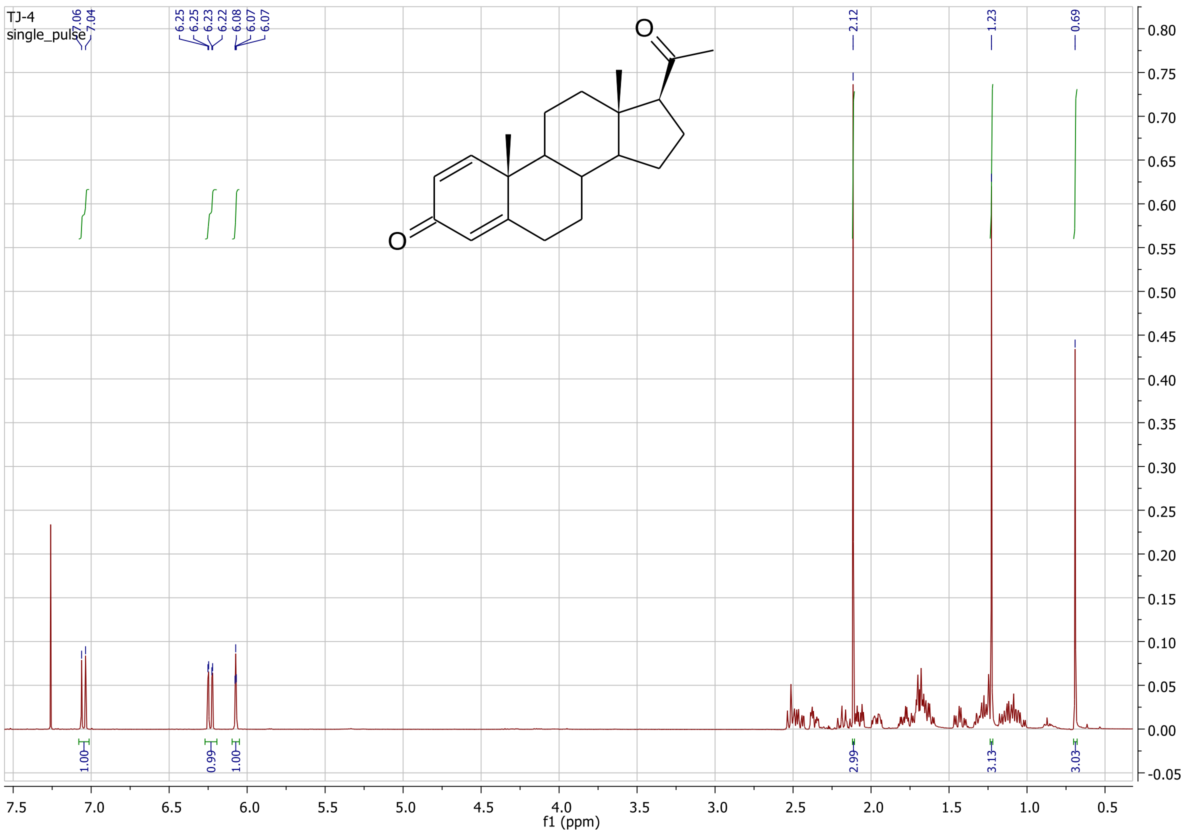

Figure S4. ^{13}C NMR spectra of pregn-1,4-diene-3,20-dione (Δ^{1}-progesterone) (**2**) ($CDCl_3$, 151 MHz)

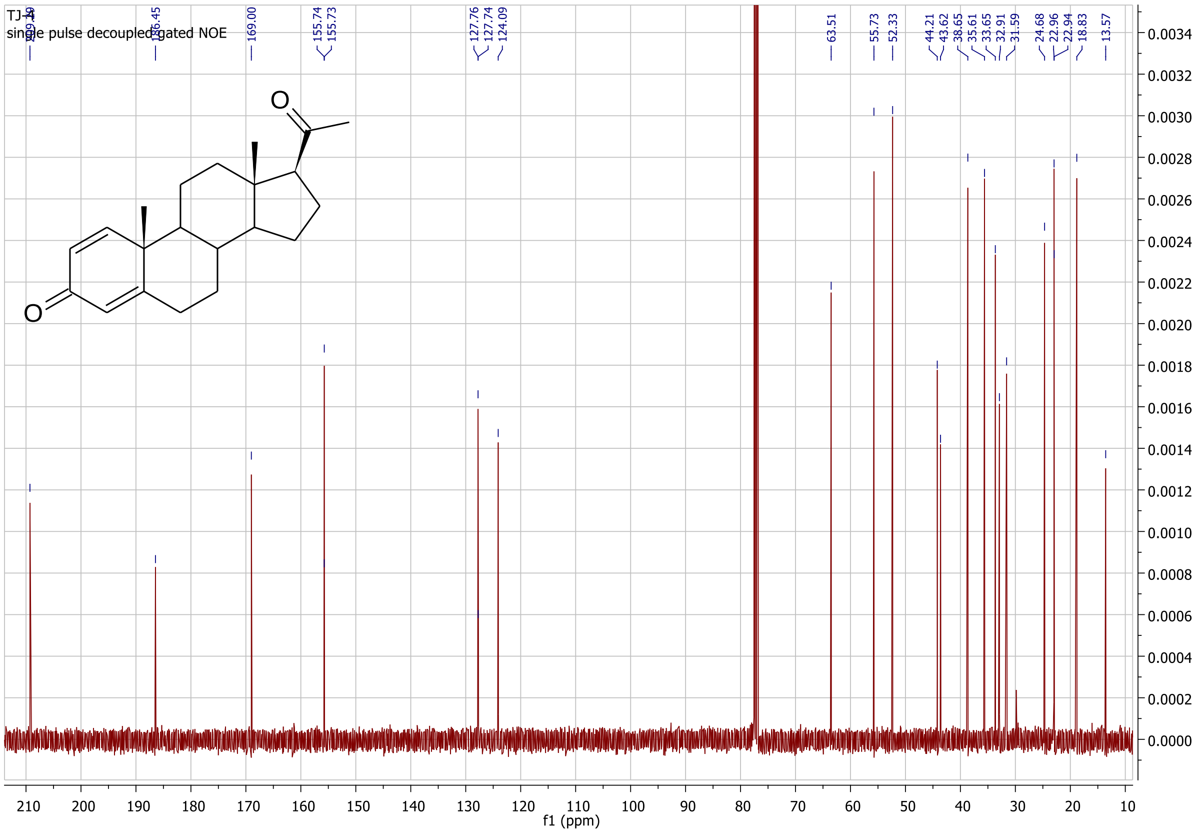

Figure S5. COSY spectrum of pregn-1,4-diene-3,20-dione (Δ^1-progesterone) (**2**) ($CDCl_3$, 600 MHz)

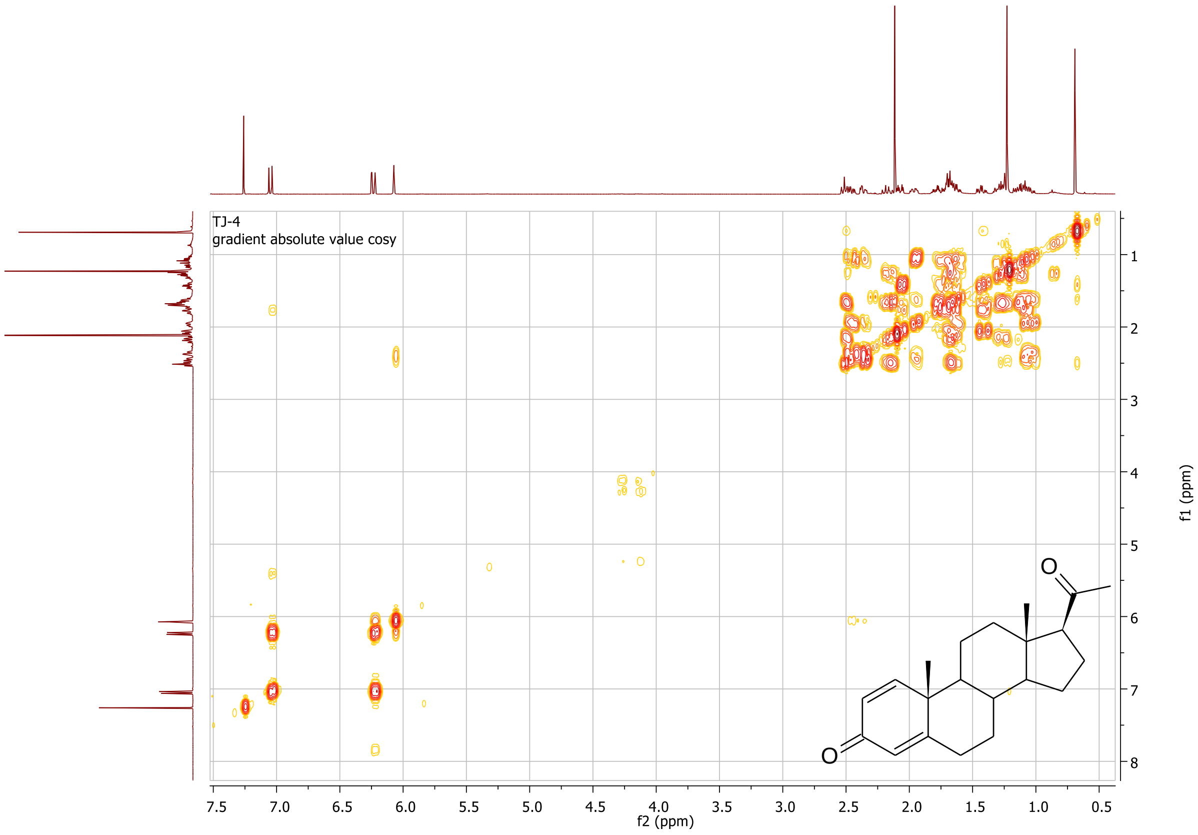

Figure S6. HSQC spectrum of pregn-1,4-diene-3,20-dione (Δ^1-progesterone) (**2**) ($CDCl_3$, 600/151 MHz)

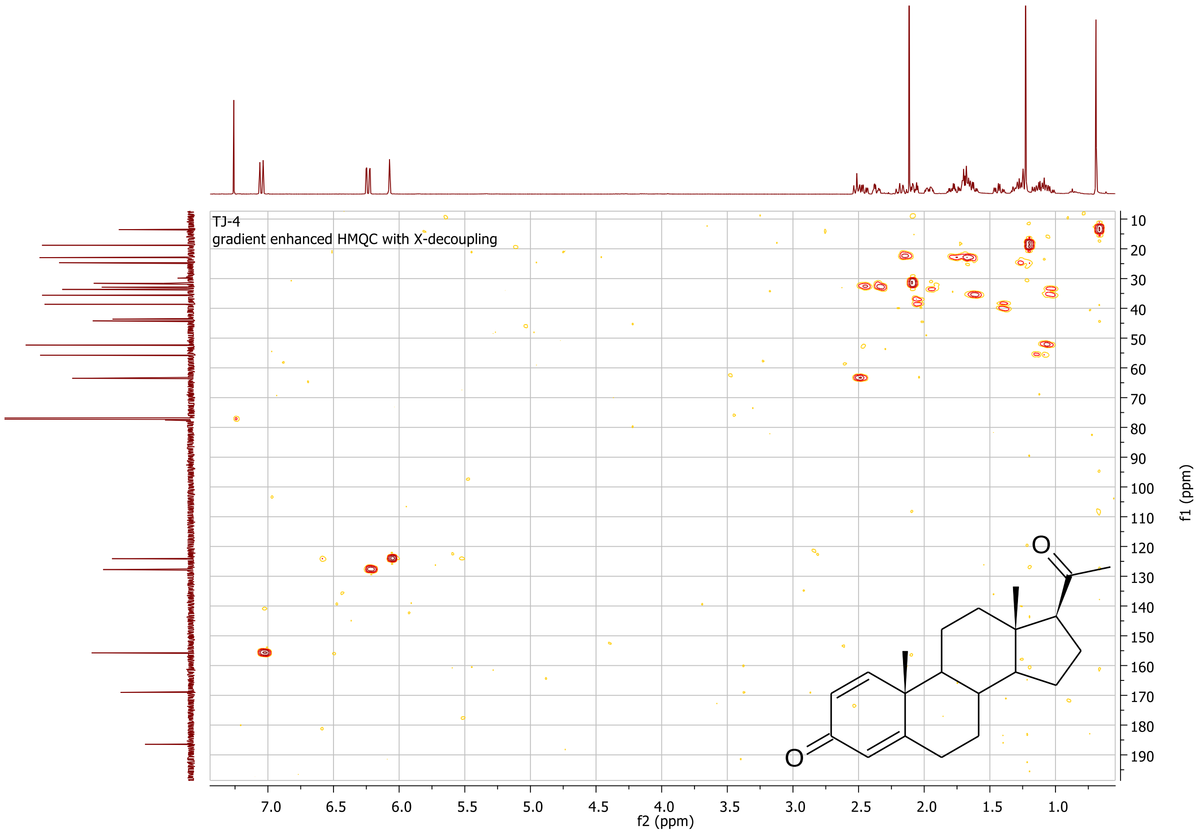

Figure S7. HMBC spectrum of pregn-1,4-diene-3,20-dione (Δ^1-progesterone) (**2**) ($CDCl_3$, 600/151 MHz)

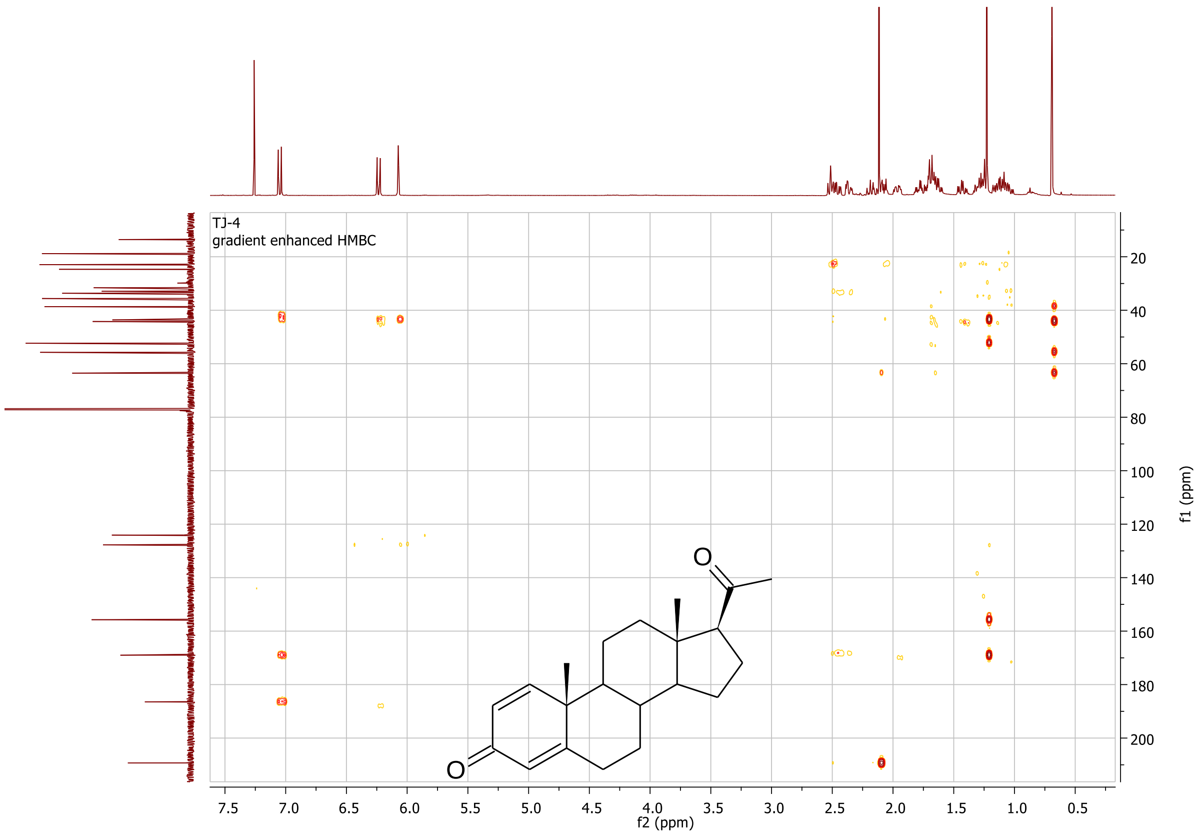

Figure S8. Predicted Boiled-Egg plot from swissADME online web tool for pregn-1,4-diene-3,20-dione (Δ^1-progesterone) (**2**)

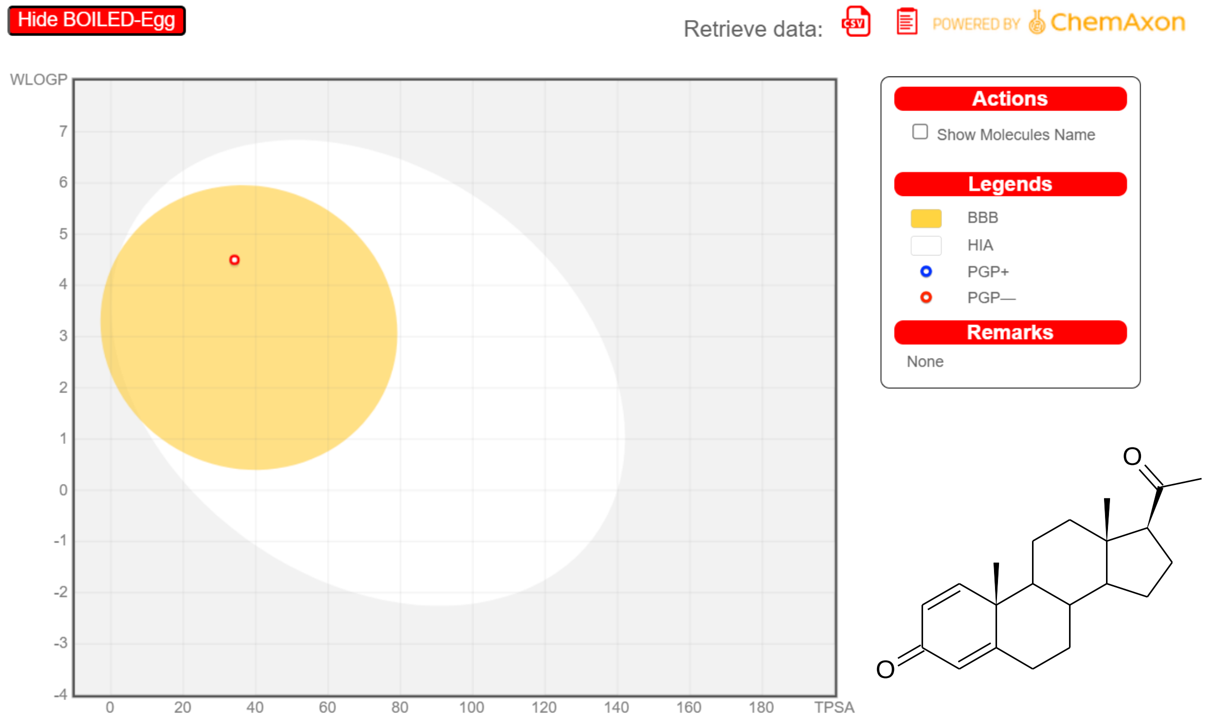

Figure S9. pregn-1,4-diene-3,20-dione (Δ^1-progesterone) (**2**) physicochemical and ADME parameters prediction using the SwissADME modelling

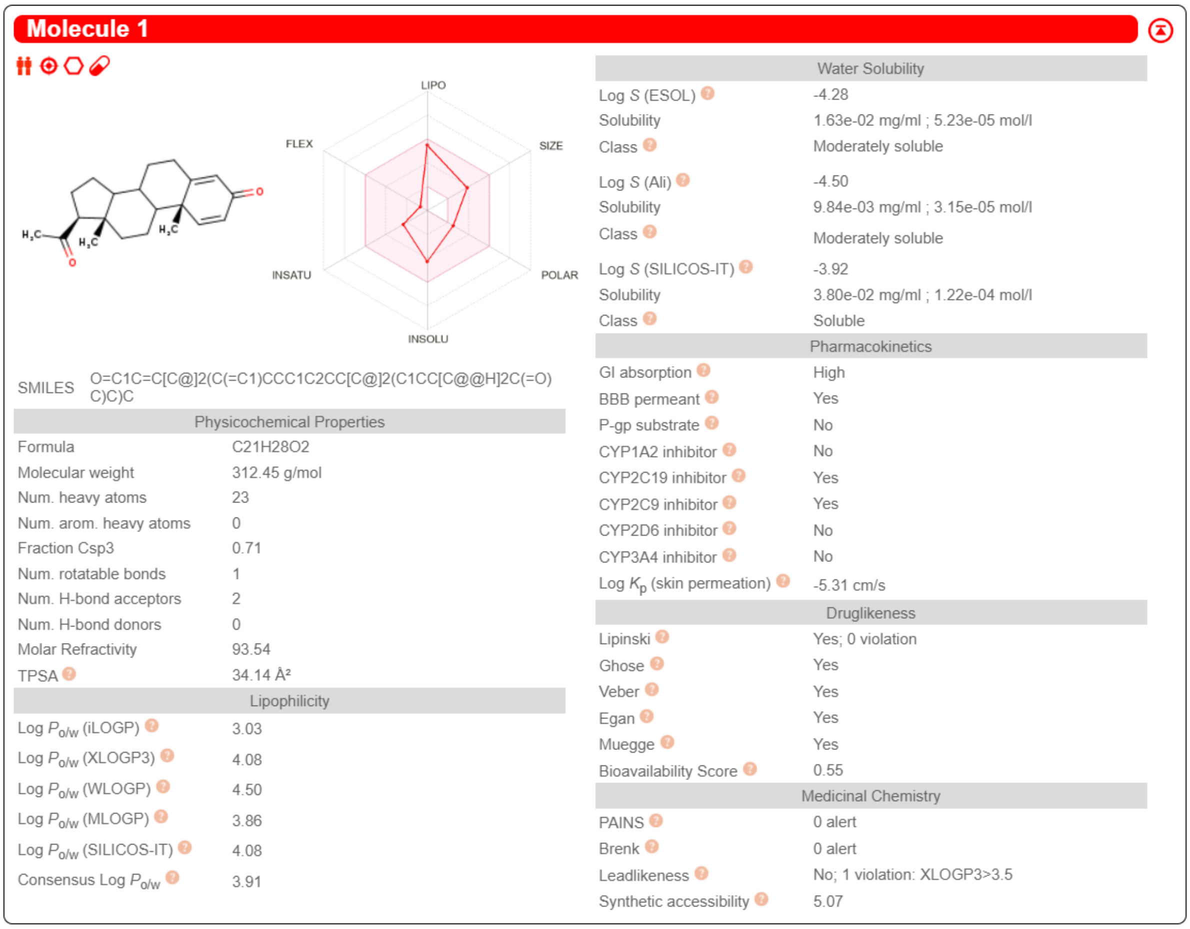

Molecule 1

SMILES O=C1C=C[C@]2(C(=C1)CCC1C2CC[C@]2(C1CC[C@@H]2C(=O)C)C)C

Physicochemical Properties	
Formula	C21H28O2
Molecular weight	312.45 g/mol
Num. heavy atoms	23
Num. arom. heavy atoms	0
Fraction Csp3	0.71
Num. rotatable bonds	1
Num. H-bond acceptors	2
Num. H-bond donors	0
Molar Refractivity	93.54
TPSA	34.14 Å²

Lipophilicity	
Log $P_{o/w}$ (iLOGP)	3.03
Log $P_{o/w}$ (XLOGP3)	4.08
Log $P_{o/w}$ (WLOGP)	4.50
Log $P_{o/w}$ (MLOGP)	3.86
Log $P_{o/w}$ (SILICOS-IT)	4.08
Consensus Log $P_{o/w}$	3.91

Water Solubility	
Log *S* (ESOL)	-4.28
Solubility	1.63e-02 mg/ml ; 5.23e-05 mol/l
Class	Moderately soluble
Log *S* (Ali)	-4.50
Solubility	9.84e-03 mg/ml ; 3.15e-05 mol/l
Class	Moderately soluble
Log *S* (SILICOS-IT)	-3.92
Solubility	3.80e-02 mg/ml ; 1.22e-04 mol/l
Class	Soluble

Pharmacokinetics	
GI absorption	High
BBB permeant	Yes
P-gp substrate	No
CYP1A2 inhibitor	No
CYP2C19 inhibitor	Yes
CYP2C9 inhibitor	Yes
CYP2D6 inhibitor	No
CYP3A4 inhibitor	No
Log K_p (skin permeation)	-5.31 cm/s

Druglikeness	
Lipinski	Yes; 0 violation
Ghose	Yes
Veber	Yes
Egan	Yes
Muegge	Yes
Bioavailability Score	0.55

Medicinal Chemistry	
PAINS	0 alert
Brenk	0 alert
Leadlikeness	No; 1 violation: XLOGP3>3.5
Synthetic accessibility	5.07

Figure S10. ^{1}H NMR spectra of 11α-hydroxypregn-1,4-diene-3,20-dione (**3**) ($CDCl_3$, 600 MHz)

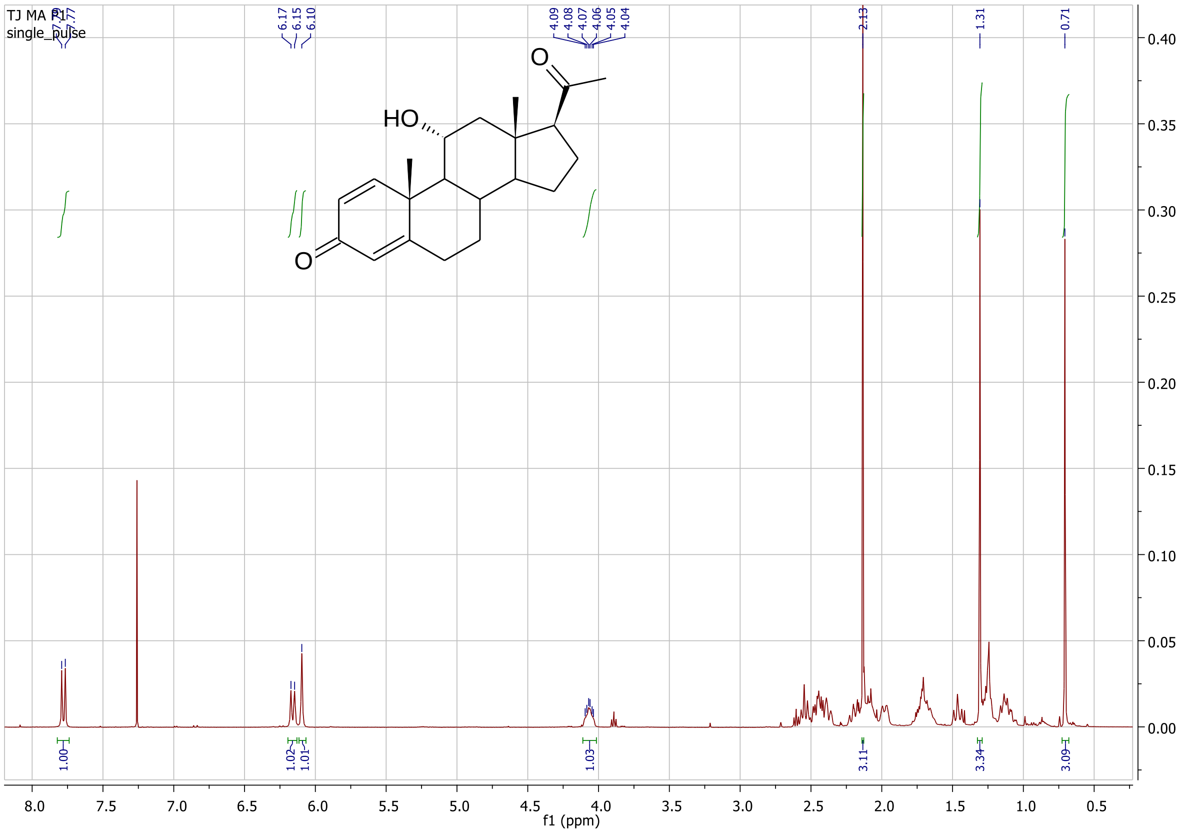

Figure S11. ^{13}C NMR spectra of 11α-hydroxypregn-1,4-diene-3,20-dione (**3**) ($CDCl_3$, 151 MHz)

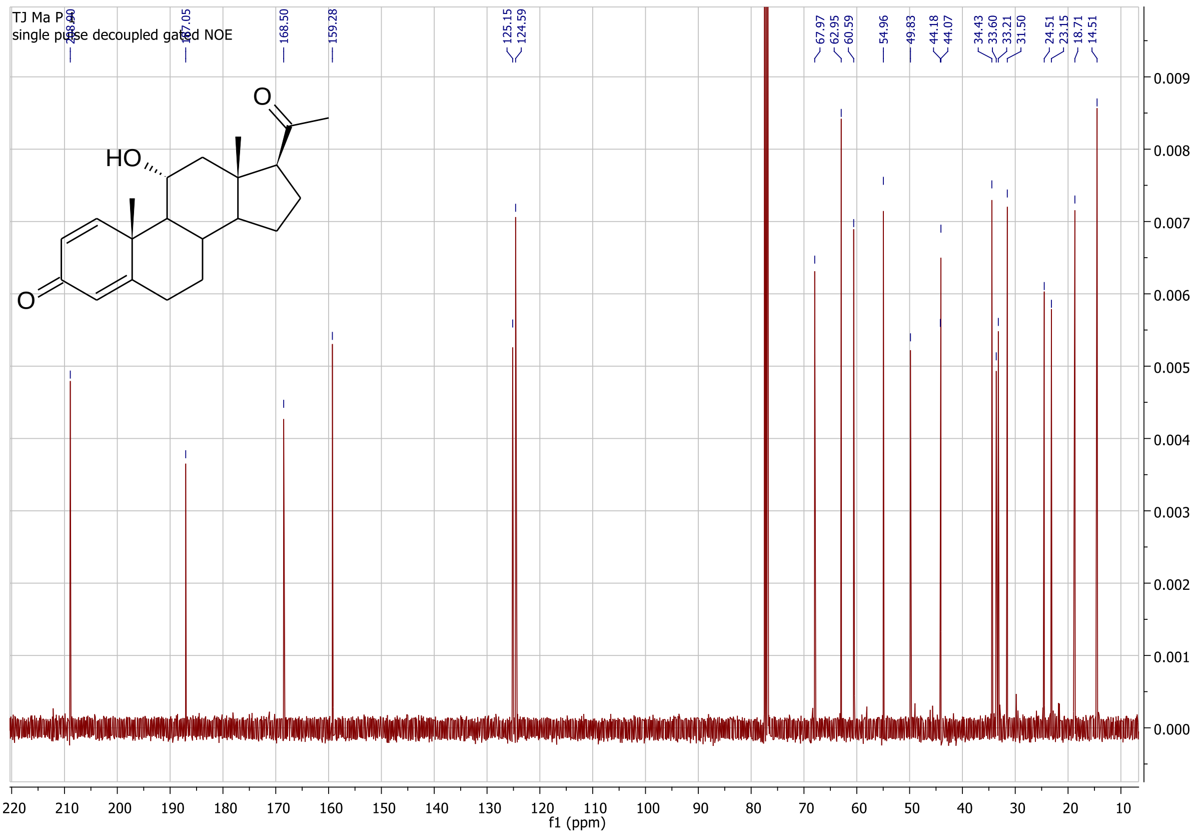

Figure S12. COSY spectrum of 11α-hydroxypregn-1,4-diene-3,20-dione (**3**) ($CDCl_3$, 600 MHz)

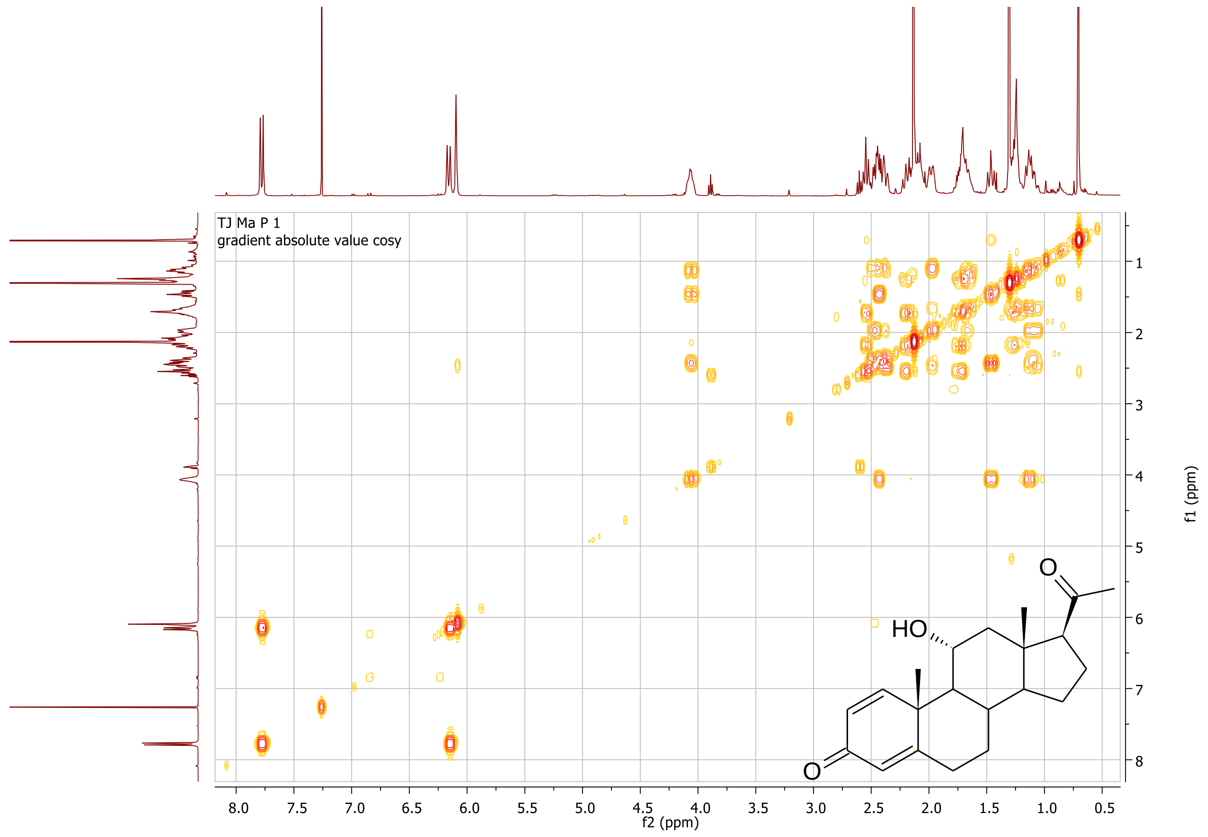

Figure S13. HSQC spectrum of 11α-hydroxypregn-1,4-diene-3,20-dione (**3**) ($CDCl_3$, 600/151 MHz)

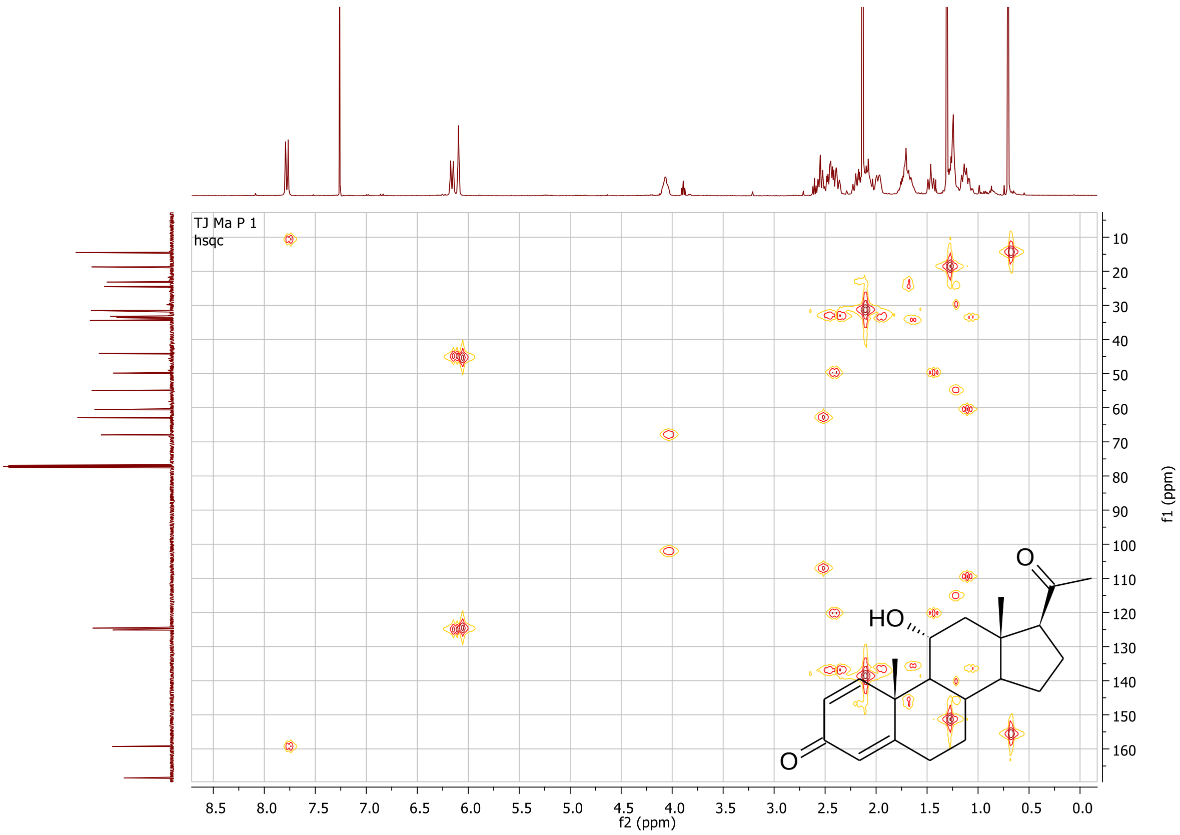

Figure S14. HMBC spectrum of 11α-hydroxypregn-1,4-diene-3,20-dione (**3**) ($CDCl_3$, 600/151 MHz)

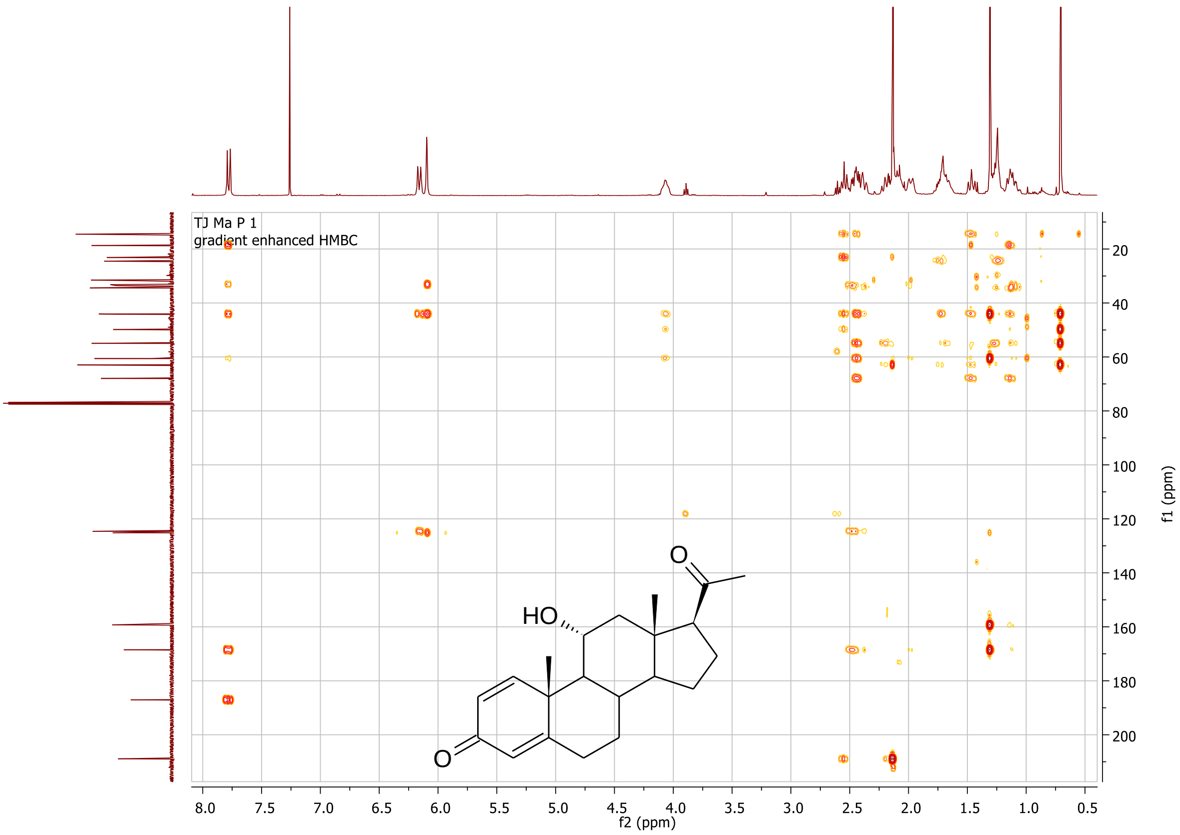

Figure S15. Predicted Boiled-Egg plot from swissADME online web tool for 11α-hydroxypregn-1,4-diene-3,20-dione (**3**)

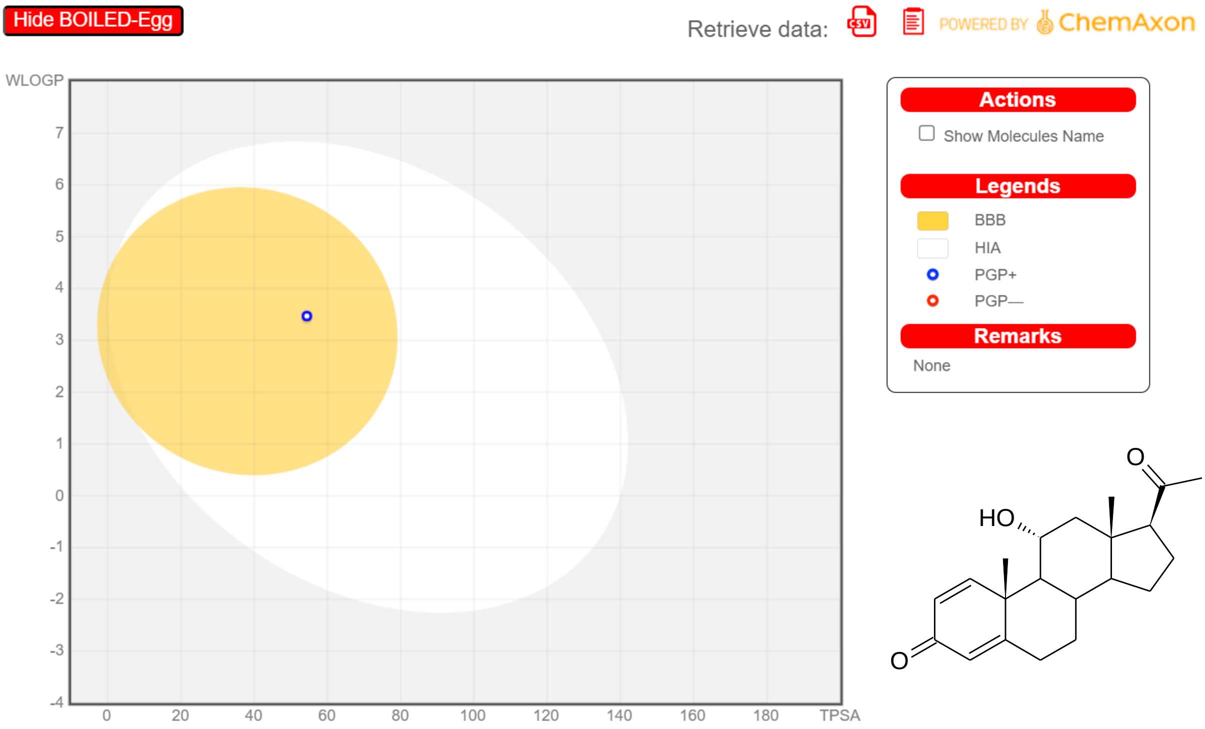

Figure S16. 11α-hydroxypregn-1,4-diene-3,20-dione (**3**) physicochemical and ADME parameters prediction using the SwissADME modelling

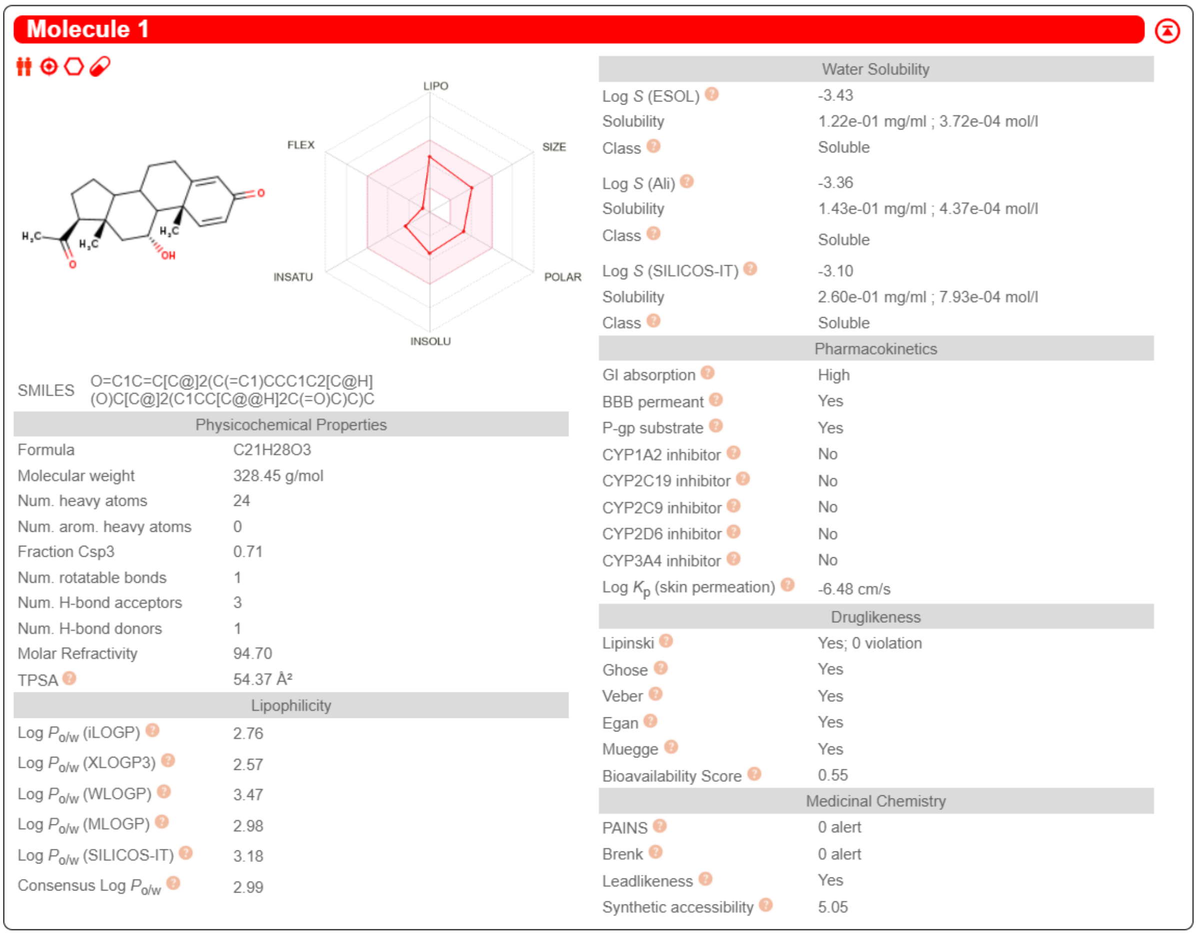
Molecule 1

LIPO, SIZE, POLAR, INSOLU, INSATU, FLEX

SMILES O=C1C=C[C@]2(C(=C1)CCC1C2[C@H](O)C[C@]2(C1CC[C@@H]2C(=O)C)C)C

Physicochemical Properties	
Formula	C21H28O3
Molecular weight	328.45 g/mol
Num. heavy atoms	24
Num. arom. heavy atoms	0
Fraction Csp3	0.71
Num. rotatable bonds	1
Num. H-bond acceptors	3
Num. H-bond donors	1
Molar Refractivity	94.70
TPSA	54.37 Å²

Lipophilicity	
Log $P_{o/w}$ (iLOGP)	2.76
Log $P_{o/w}$ (XLOGP3)	2.57
Log $P_{o/w}$ (WLOGP)	3.47
Log $P_{o/w}$ (MLOGP)	2.98
Log $P_{o/w}$ (SILICOS-IT)	3.18
Consensus Log $P_{o/w}$	2.99

Water Solubility	
Log *S* (ESOL)	-3.43
Solubility	1.22e-01 mg/ml ; 3.72e-04 mol/l
Class	Soluble
Log *S* (Ali)	-3.36
Solubility	1.43e-01 mg/ml ; 4.37e-04 mol/l
Class	Soluble
Log *S* (SILICOS-IT)	-3.10
Solubility	2.60e-01 mg/ml ; 7.93e-04 mol/l
Class	Soluble

Pharmacokinetics	
GI absorption	High
BBB permeant	Yes
P-gp substrate	Yes
CYP1A2 inhibitor	No
CYP2C19 inhibitor	No
CYP2C9 inhibitor	No
CYP2D6 inhibitor	No
CYP3A4 inhibitor	No
Log K_p (skin permeation)	-6.48 cm/s

Druglikeness	
Lipinski	Yes; 0 violation
Ghose	Yes
Veber	Yes
Egan	Yes
Muegge	Yes
Bioavailability Score	0.55

Medicinal Chemistry	
PAINS	0 alert
Brenk	0 alert
Leadlikeness	Yes
Synthetic accessibility	5.05

Figure S17. ^{1}H NMR spectra of 6β,11α-dihydroxypregn-1,4-diene-3,20-dione (**4**) (DMSO-d_6, 600 MHz)

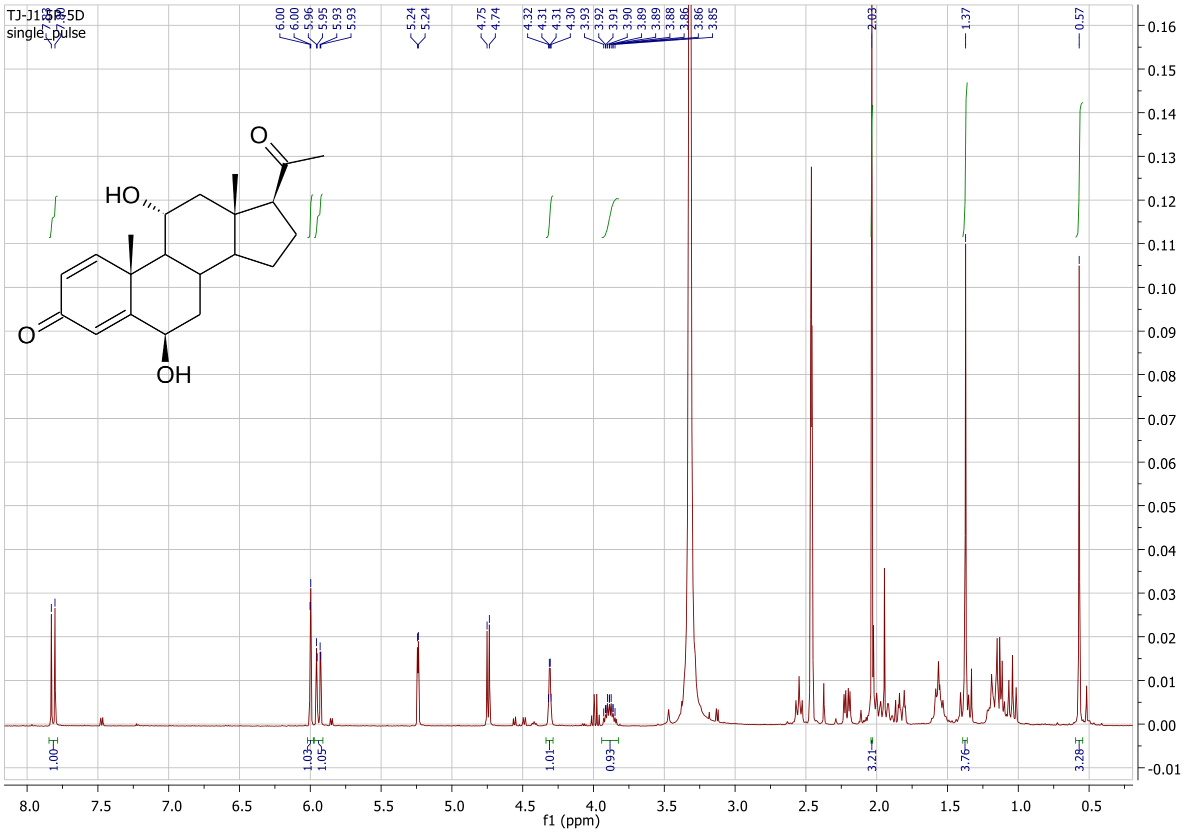

Figure S18. ^{13}C NMR spectra of 6β,11α-dihydroxypregn-1,4-diene-3,20-dione (**4**) (DMSO-d_6, 151 MHz)

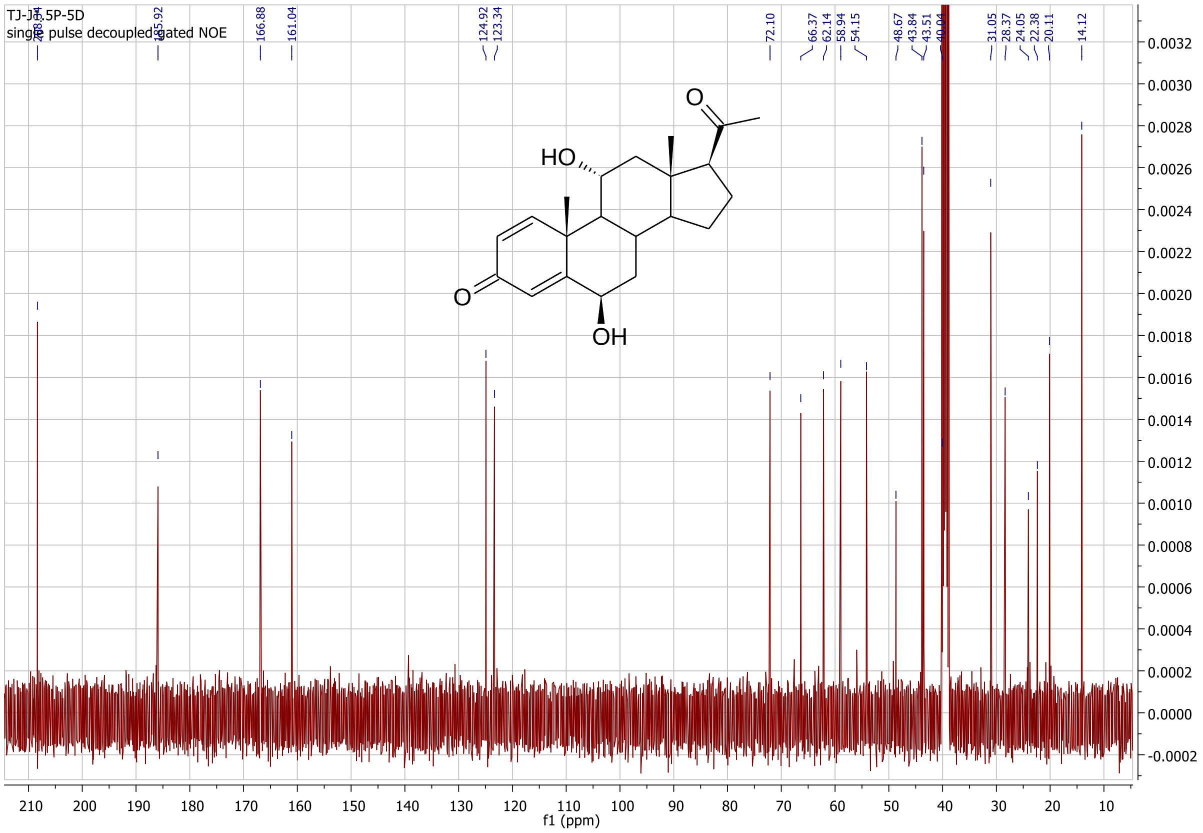

Figure S19. COSY spectrum of 6β,11α-dihydroxypregn-1,4-diene-3,20-dione (**4**) (DMSO-d_6, 600 MHz)

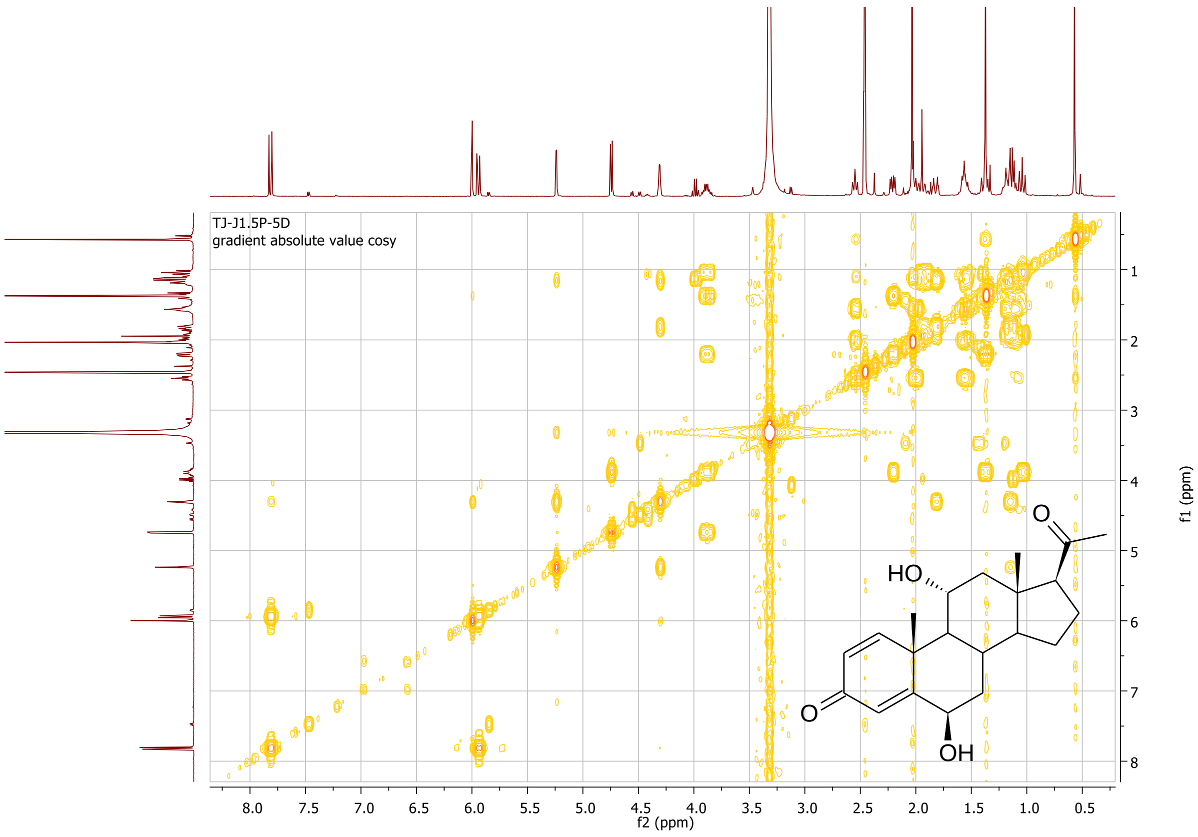

Figure S20. HSQC spectrum of 6β,11α-dihydroxypregn-1,4-diene-3,20-dione (**4**) (DMSO-d_6, 600/151 MHz)

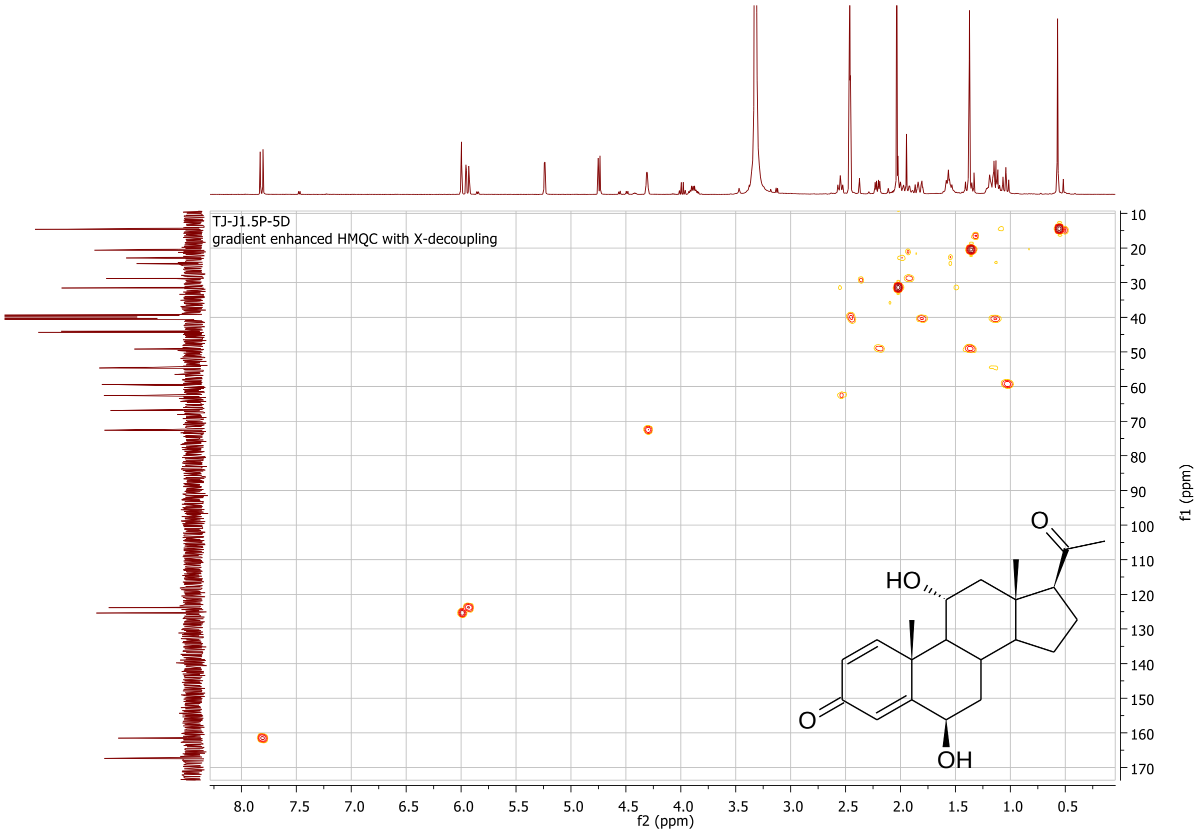

Figure S21. HMBC spectrum of 6β,11α-dihydroxypregn-1,4-diene-3,20-dione (**4**) (DMSO-d_6, 600/151 MHz)

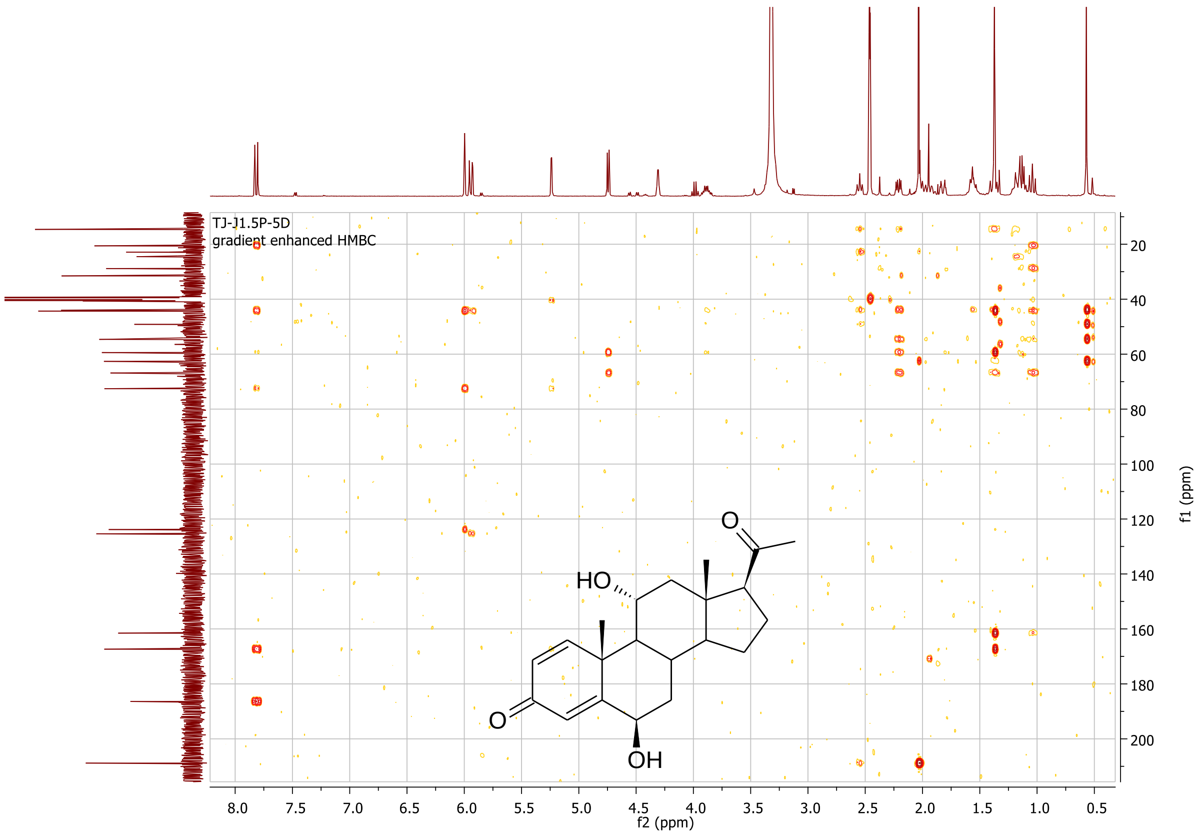

Figure S22. Predicted Boiled-Egg plot from swissADME online web tool for 6β,11α-dihydroxypregn-1,4-diene-3,20-dione (**4**)

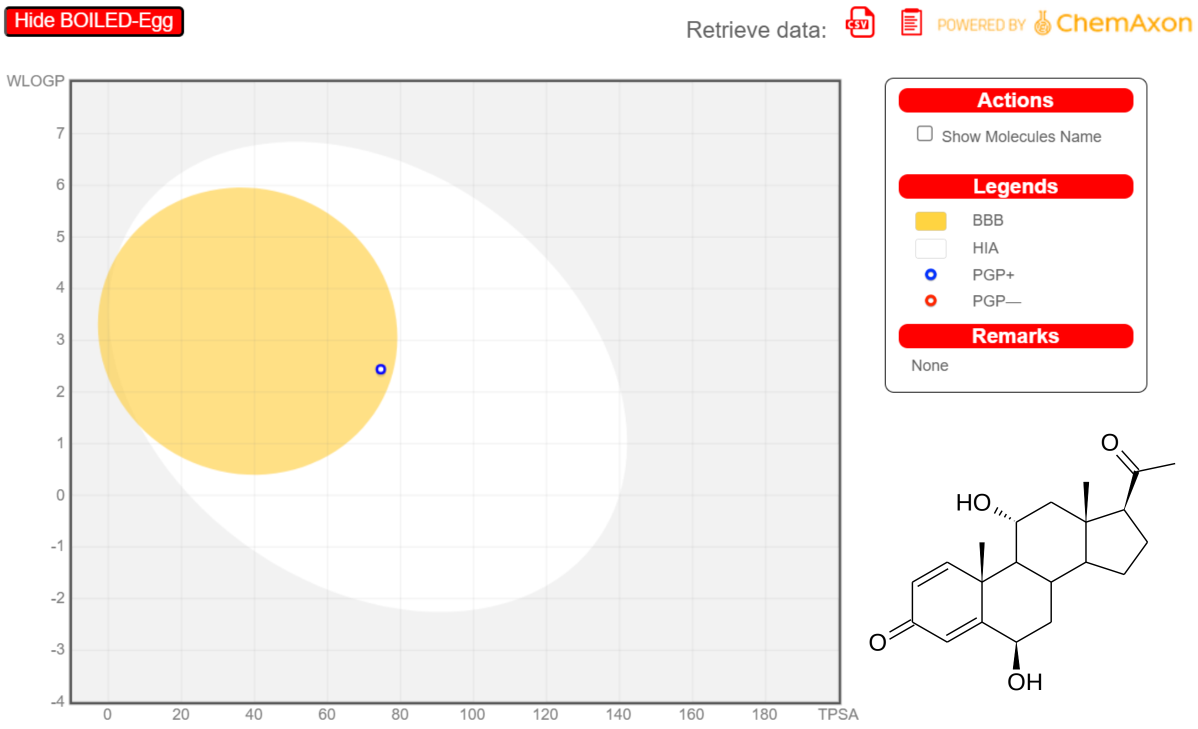

Figure S23. 6β,11α-dihydroxypregn-1,4-diene-3,20-dione (**4**) physicochemical and ADME parameters prediction using the SwissADME modelling

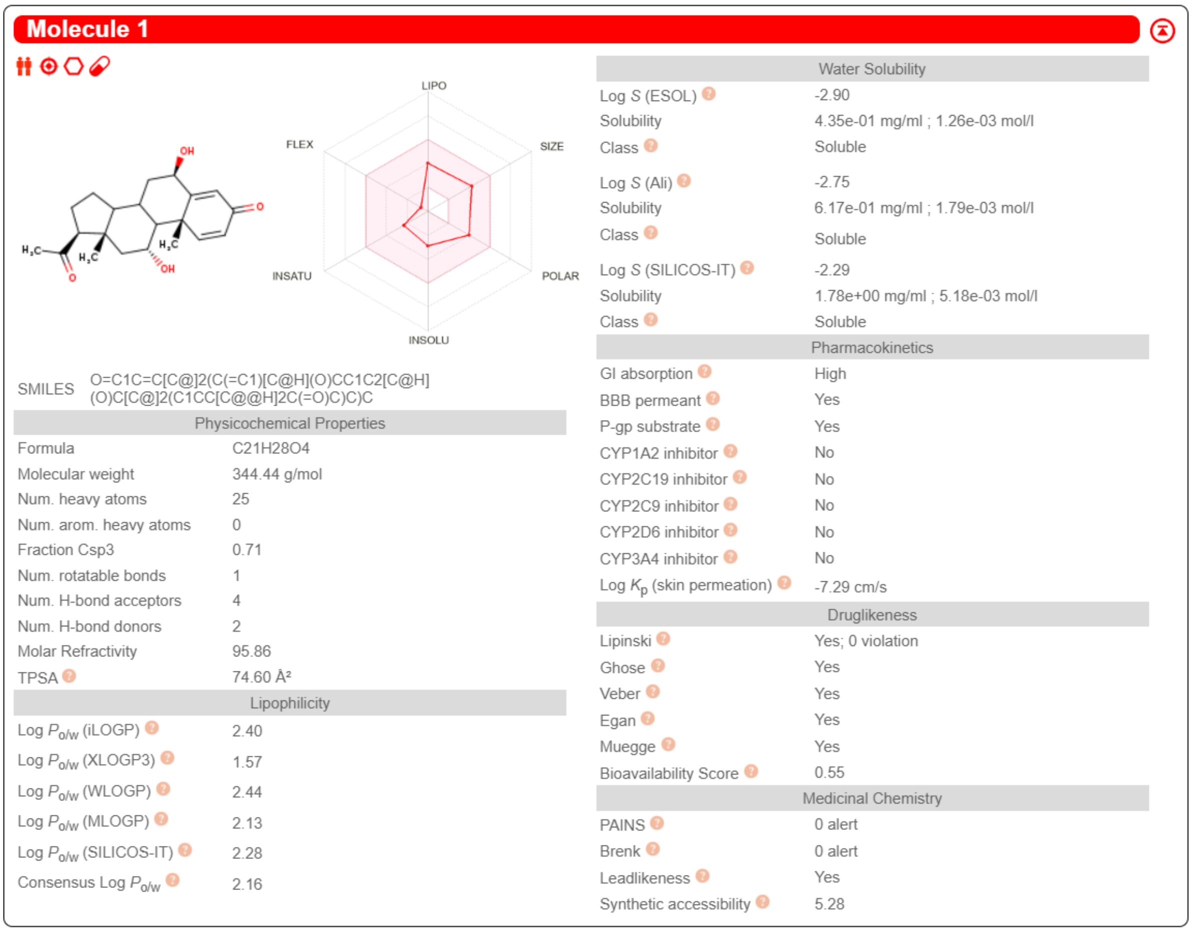

SMILES O=C1C=C[C@]2(C(=C1)[C@H](O)CC1C2[C@H](O)C[C@]2(C1CC[C@@H]2C(=O)C)C)C

Physicochemical Properties	
Formula	C21H28O4
Molecular weight	344.44 g/mol
Num. heavy atoms	25
Num. arom. heavy atoms	0
Fraction Csp3	0.71
Num. rotatable bonds	1
Num. H-bond acceptors	4
Num. H-bond donors	2
Molar Refractivity	95.86
TPSA	74.60 Å²

Lipophilicity	
Log $P_{o/w}$ (iLOGP)	2.40
Log $P_{o/w}$ (XLOGP3)	1.57
Log $P_{o/w}$ (WLOGP)	2.44
Log $P_{o/w}$ (MLOGP)	2.13
Log $P_{o/w}$ (SILICOS-IT)	2.28
Consensus Log $P_{o/w}$	2.16

Water Solubility	
Log *S* (ESOL)	-2.90
Solubility	4.35e-01 mg/ml ; 1.26e-03 mol/l
Class	Soluble
Log *S* (Ali)	-2.75
Solubility	6.17e-01 mg/ml ; 1.79e-03 mol/l
Class	Soluble
Log *S* (SILICOS-IT)	-2.29
Solubility	1.78e+00 mg/ml ; 5.18e-03 mol/l
Class	Soluble

Pharmacokinetics	
GI absorption	High
BBB permeant	Yes
P-gp substrate	Yes
CYP1A2 inhibitor	No
CYP2C19 inhibitor	No
CYP2C9 inhibitor	No
CYP2D6 inhibitor	No
CYP3A4 inhibitor	No
Log K_p (skin permeation)	-7.29 cm/s

Druglikeness	
Lipinski	Yes; 0 violation
Ghose	Yes
Veber	Yes
Egan	Yes
Muegge	Yes
Bioavailability Score	0.55

Medicinal Chemistry	
PAINS	0 alert
Brenk	0 alert
Leadlikeness	Yes
Synthetic accessibility	5.28

Figure S24. ^{1}H NMR spectra of 6β-hydroxypregn-1,4-diene-3,11,20-trione (**5**) ($CDCl_3$, 600 MHz)

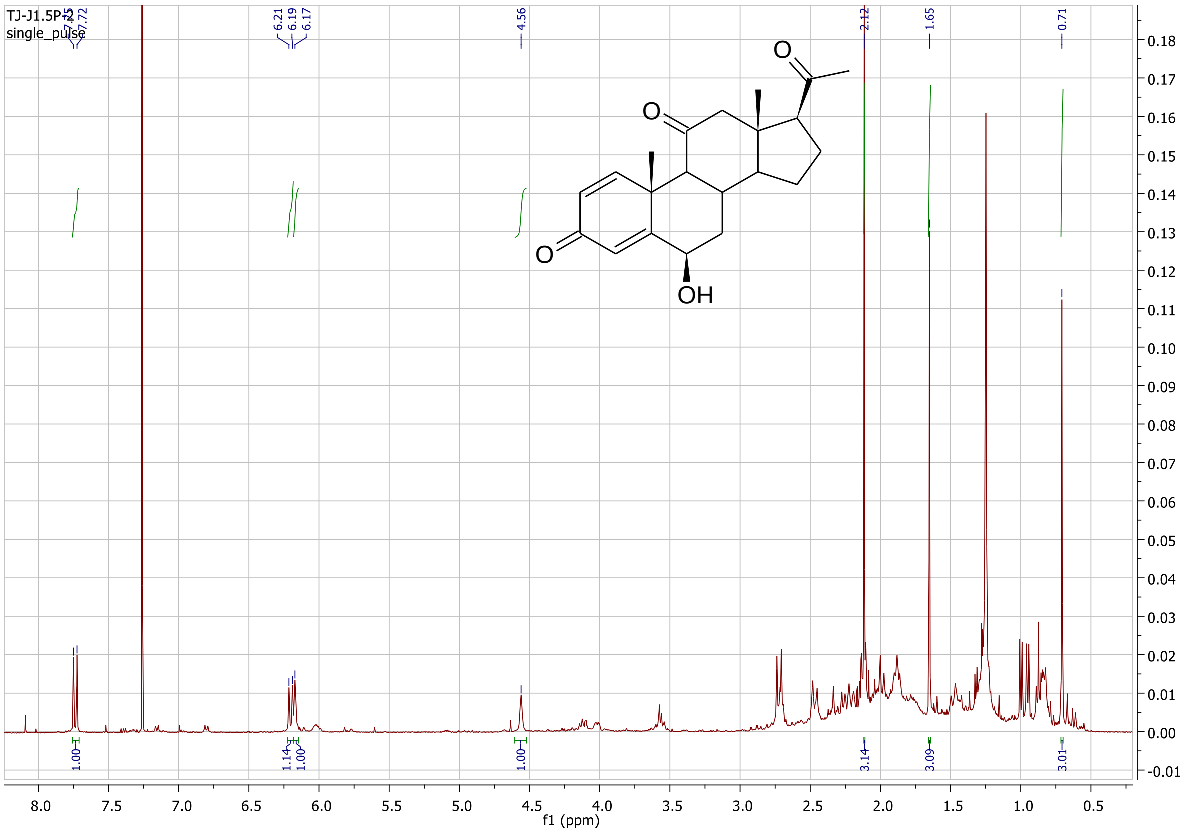

Figure S25. ^{13}C NMR spectra of 6β-hydroxypregn-1,4-diene-3,11,20-trione (**5**) ($CDCl_3$, 151 MHz)

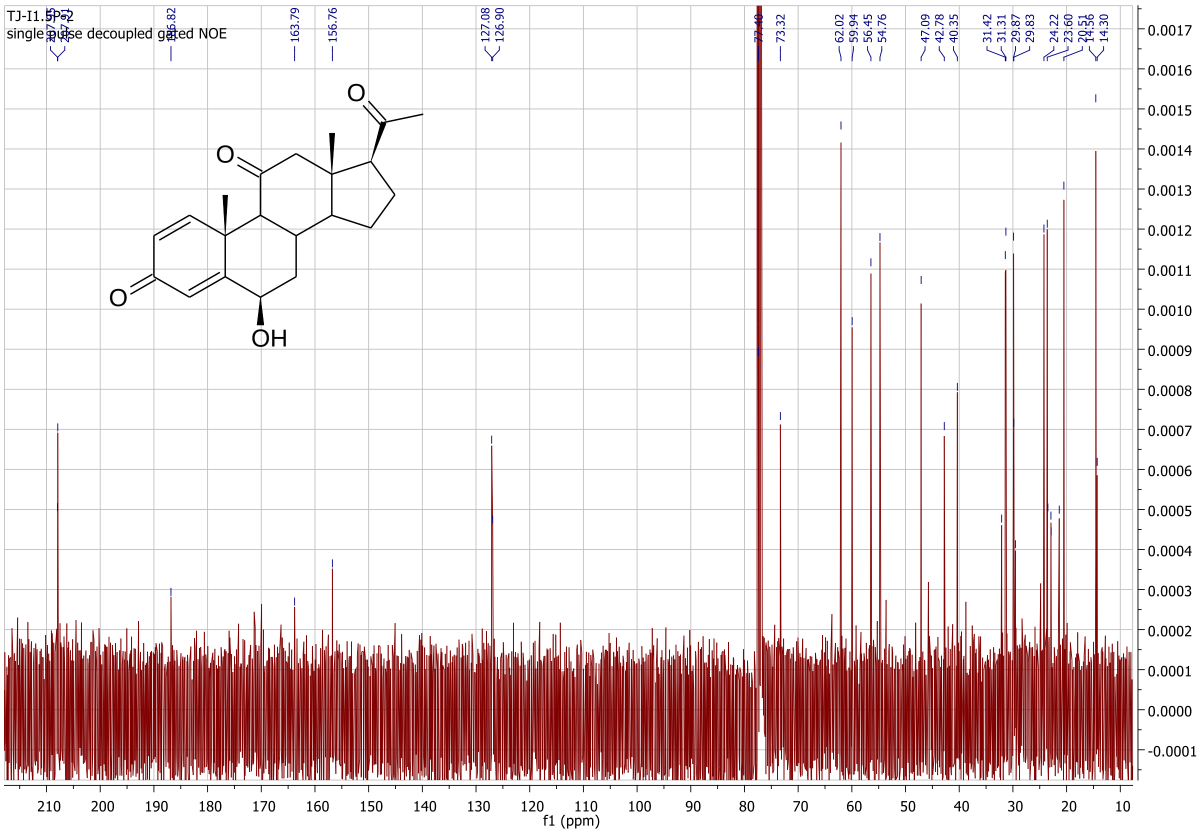

Figure S26. COSY spectrum of 6β-hydroxypregn-1,4-diene-3,11,20-trione (**5**) ($CDCl_3$, 600 MHz)

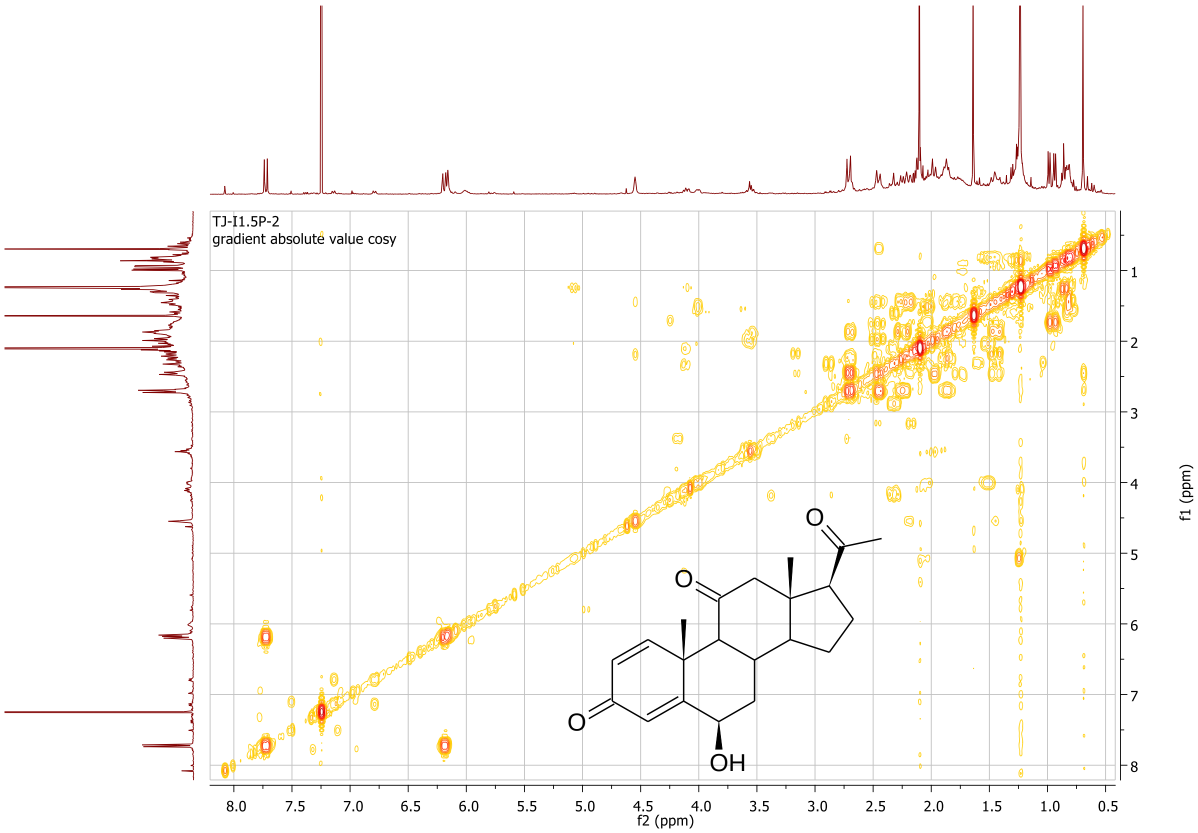

Figure S27. HSQC spectrum of 6β-hydroxypregn-1,4-diene-3,11,20-trione (**5**) ($CDCl_3$, 600/151 MHz)

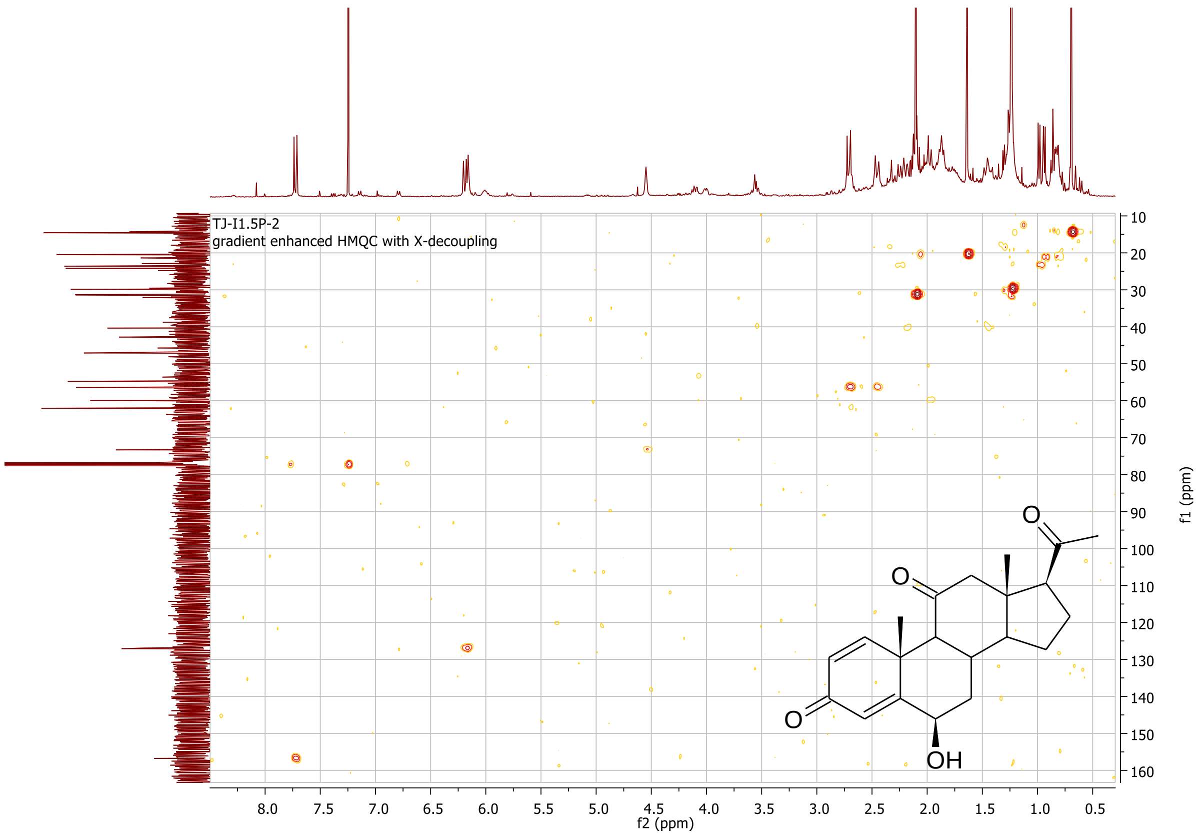

Figure S28. HMBC spectrum of 6β-hydroxypregn-1,4-diene-3,11,20-trione (**5**) ($CDCl_3$, 600/151 MHz)

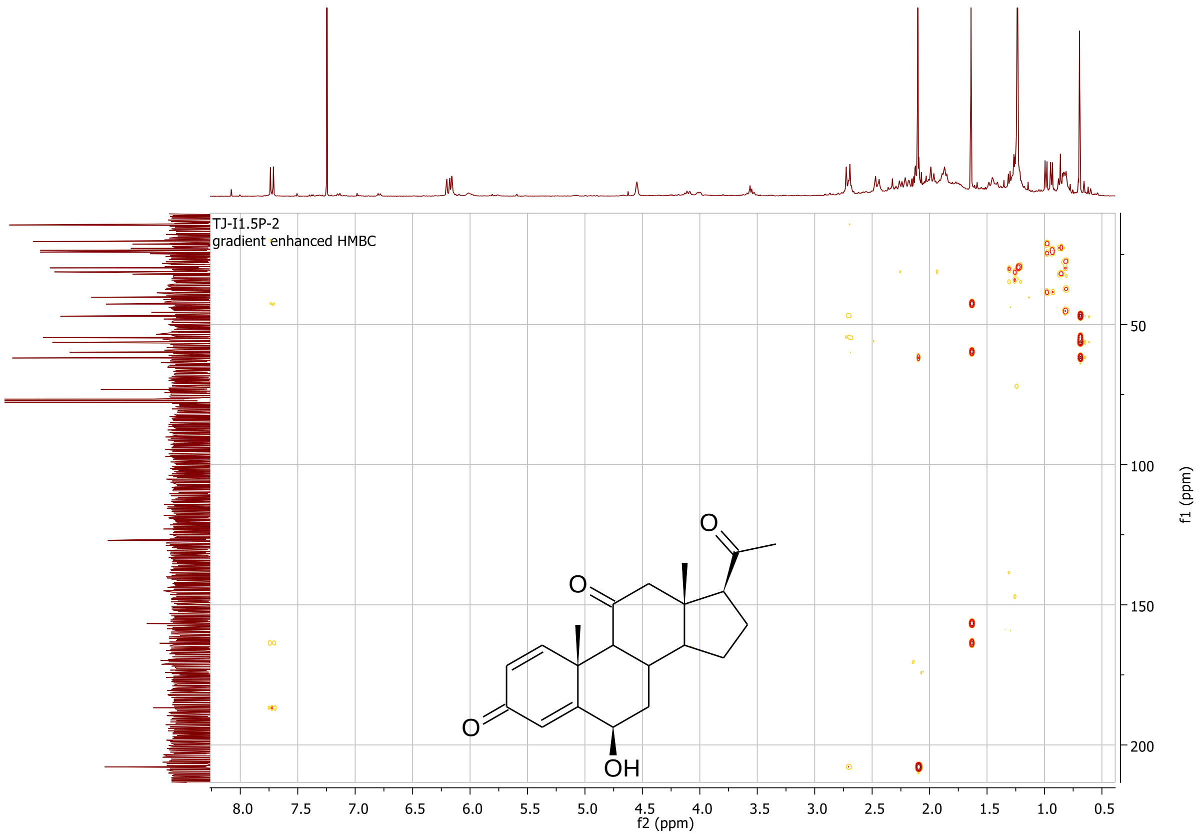

Figure S29. Predicted Boiled-Egg plot from swissADME online web tool for 6β-hydroxypregn-1,4-diene-3,11,20-trione (**5**)

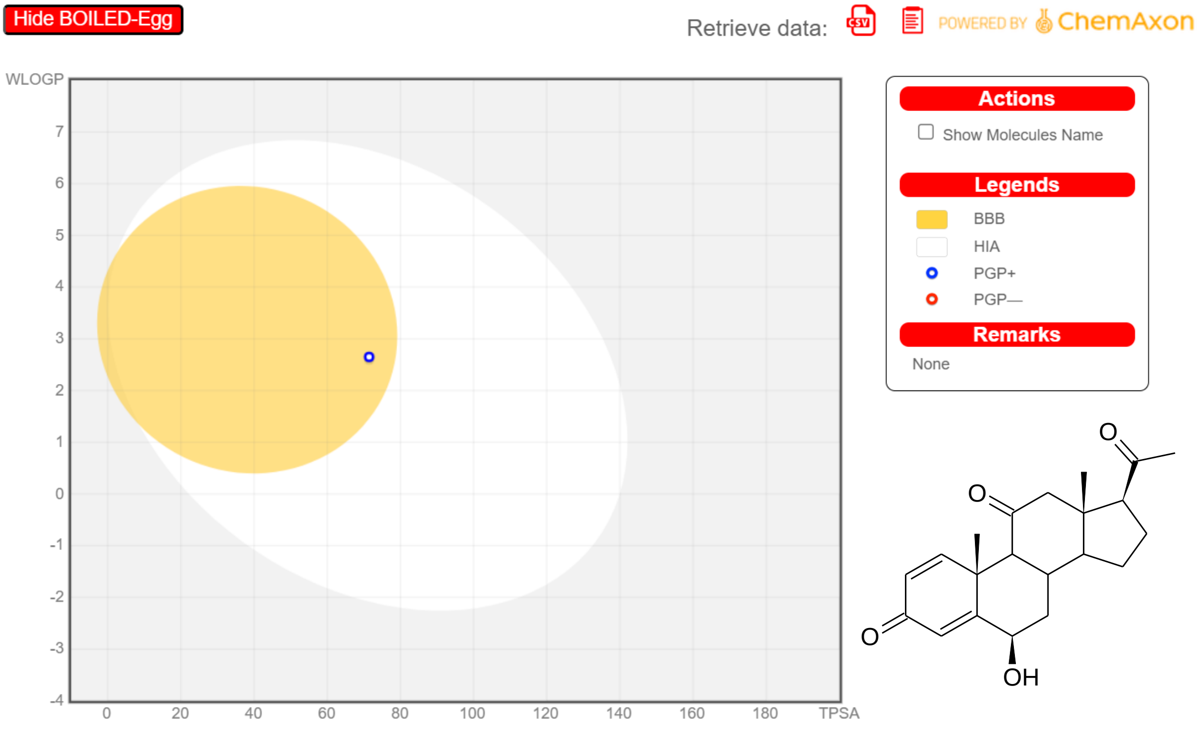

Figure S30. 6β-hydroxypregn-1,4-diene-3,11,20-trione (**5**) physicochemical and ADME parameters prediction using the SwissADME modelling

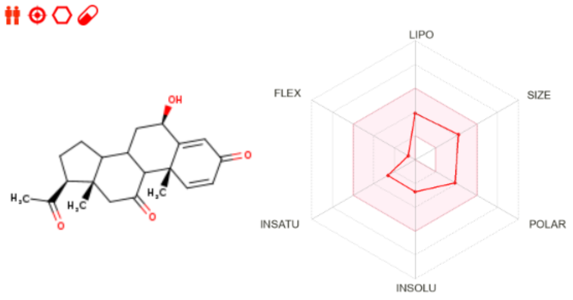

Molecule 1

SMILES O=C1C=C[C@]2(C(=C1)[C@H](O)CC1C2C(=O)C[C@]2(C1CC[C@@H]2C(=O)C)C)C

Physicochemical Properties	
Formula	C21H26O4
Molecular weight	342.43 g/mol
Num. heavy atoms	25
Num. arom. heavy atoms	0
Fraction Csp3	0.67
Num. rotatable bonds	1
Num. H-bond acceptors	4
Num. H-bond donors	1
Molar Refractivity	94.90
TPSA	71.44 Å²

Lipophilicity	
Log $P_{o/w}$ (iLOGP)	2.22
Log $P_{o/w}$ (XLOGP3)	1.29
Log $P_{o/w}$ (WLOGP)	2.65
Log $P_{o/w}$ (MLOGP)	2.04
Log $P_{o/w}$ (SILICOS-IT)	2.87
Consensus Log $P_{o/w}$	2.22

Water Solubility	
Log *S* (ESOL)	-2.71
Solubility	6.68e-01 mg/ml ; 1.95e-03 mol/l
Class	Soluble
Log *S* (Ali)	-2.39
Solubility	1.39e+00 mg/ml ; 4.07e-03 mol/l
Class	Soluble
Log *S* (SILICOS-IT)	-2.98
Solubility	3.58e-01 mg/ml ; 1.04e-03 mol/l
Class	Soluble

Pharmacokinetics	
GI absorption	High
BBB permeant	Yes
P-gp substrate	Yes
CYP1A2 inhibitor	No
CYP2C19 inhibitor	No
CYP2C9 inhibitor	No
CYP2D6 inhibitor	No
CYP3A4 inhibitor	No
Log K_p (skin permeation)	-7.47 cm/s

Druglikeness	
Lipinski	Yes; 0 violation
Ghose	Yes
Veber	Yes
Egan	Yes
Muegge	Yes
Bioavailability Score	0.55

Medicinal Chemistry	
PAINS	0 alert
Brenk	0 alert
Leadlikeness	Yes
Synthetic accessibility	5.19

Figure S31. ^{1}H NMR spectra of 6β,17α-dihydroxypregn-1,4-diene-3,20-dione (**6**) and 6β,17β-dihydroxyandrost-1,4-diene-3-one (**7**) ($CDCl_3$, 600 MHz)

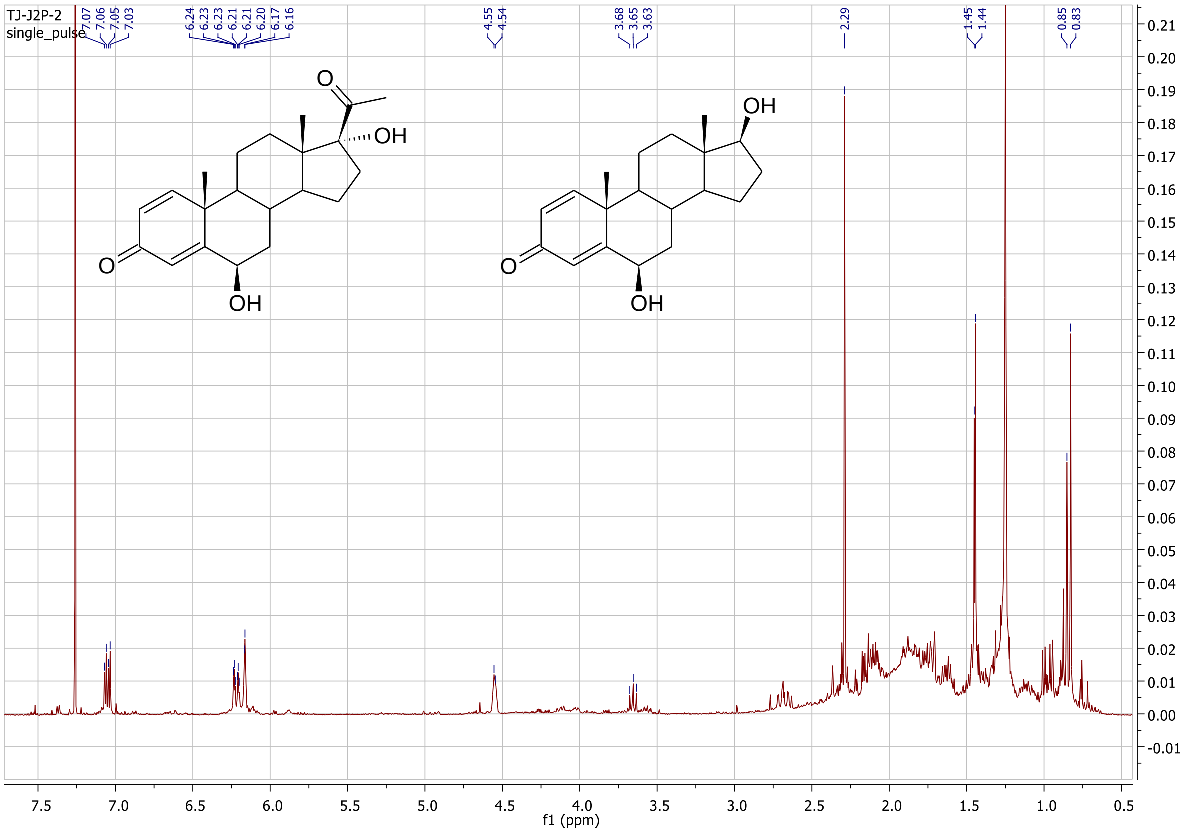

Figure S32. ^{13}C NMR spectra of 6β,17α-dihydroxypregn-1,4-diene-3,20-dione (**6**) and 6β,17β-dihydroxyandrost-1,4-diene-3-one (**7**) ($CDCl_3$, 151 MHz)

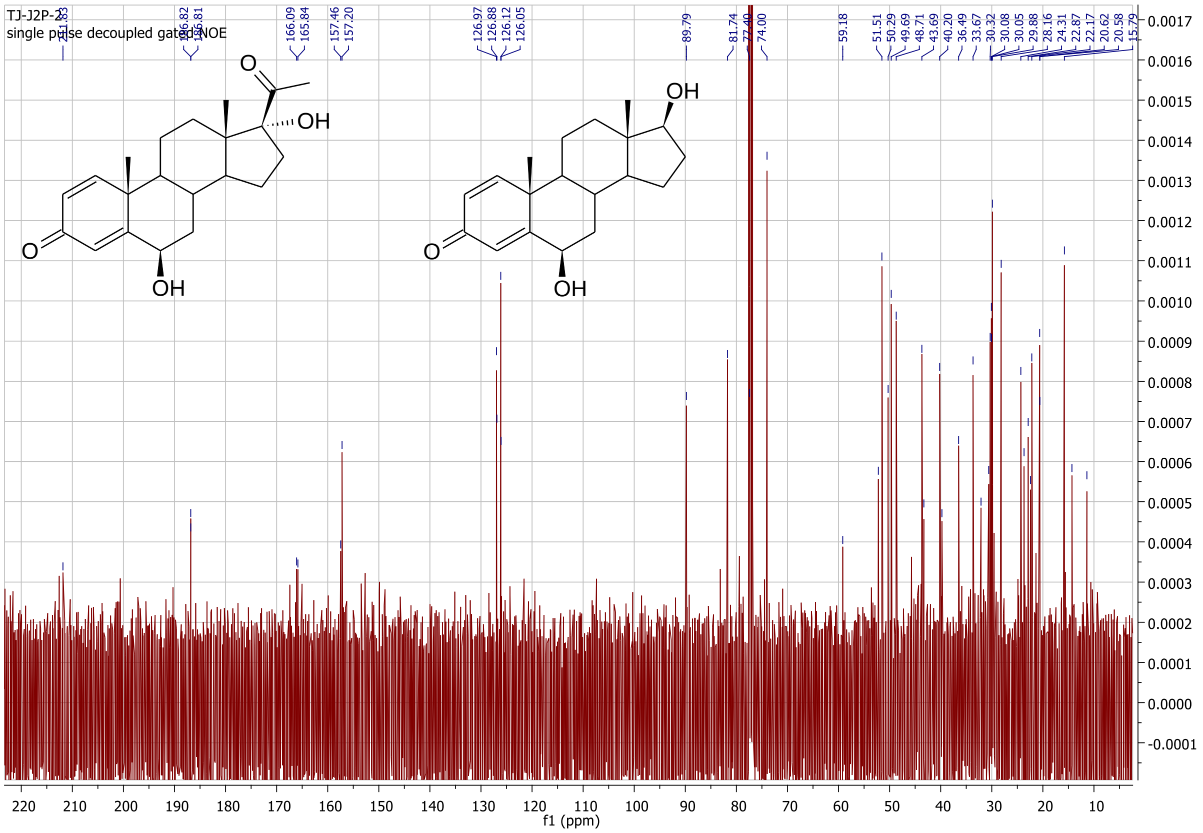

Figure S33. COSY spectrum of 6β,17α-dihydroxypregn-1,4-diene-3,20-dione (**6**) and 6β,17β-dihydroxyandrost-1,4-diene-3-one (**7**) ($CDCl_3$, 600 MHz)

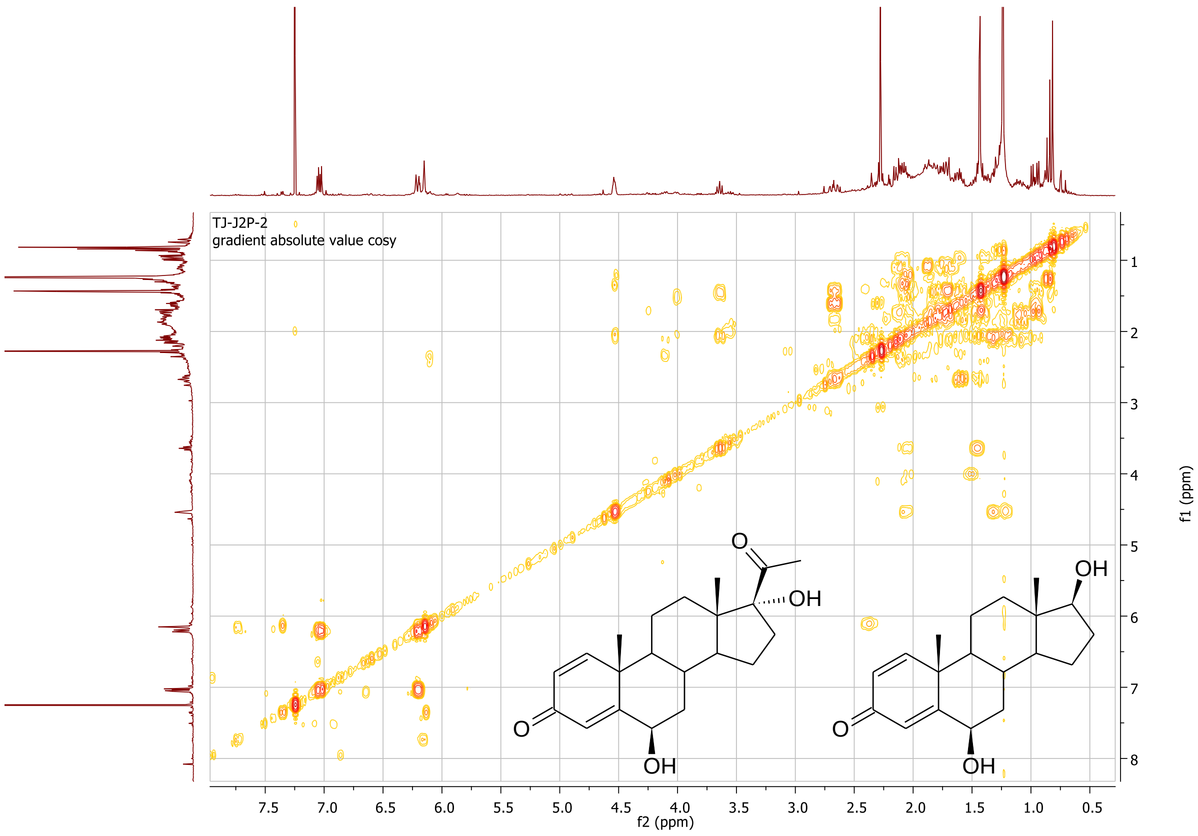

Figure S34. HSQC spectrum of 6β,17α-dihydroxypregn-1,4-diene-3,20-dione (**6**) and 6β,17β-dihydroxyandrost-1,4-diene-3-one (**7**) ($CDCl_3$, 600/151 MHz)

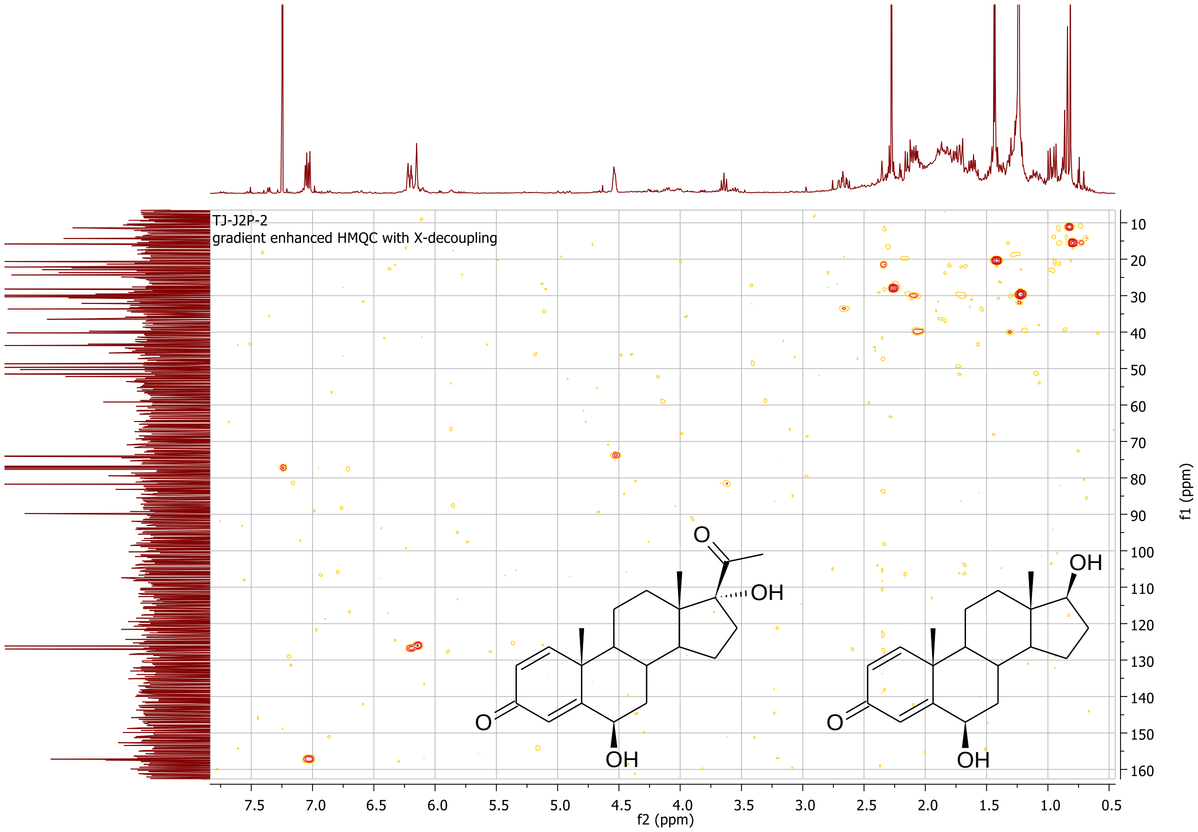

Figure S35. HMBC spectrum of 6β,17α-dihydroxypregn-1,4-diene-3,20-dione (**6**) and 6β,17β-dihydroxyandrost-1,4-diene-3-one (**7**) ($CDCl_3$, 600/151 MHz)

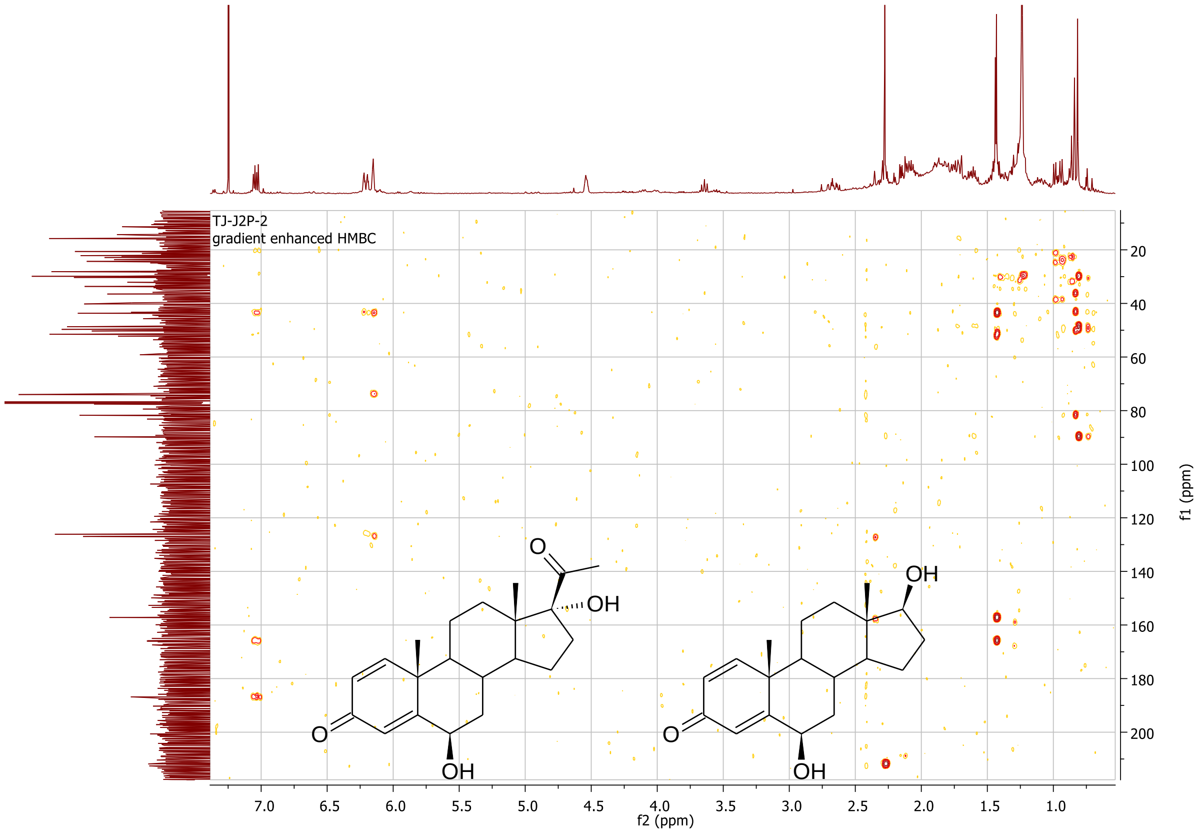

Figure S36. Predicted Boiled-Egg plot from swissADME online web tool for 6β,17α-dihydroxypregn-1,4-diene-3,20-dione (**6**)

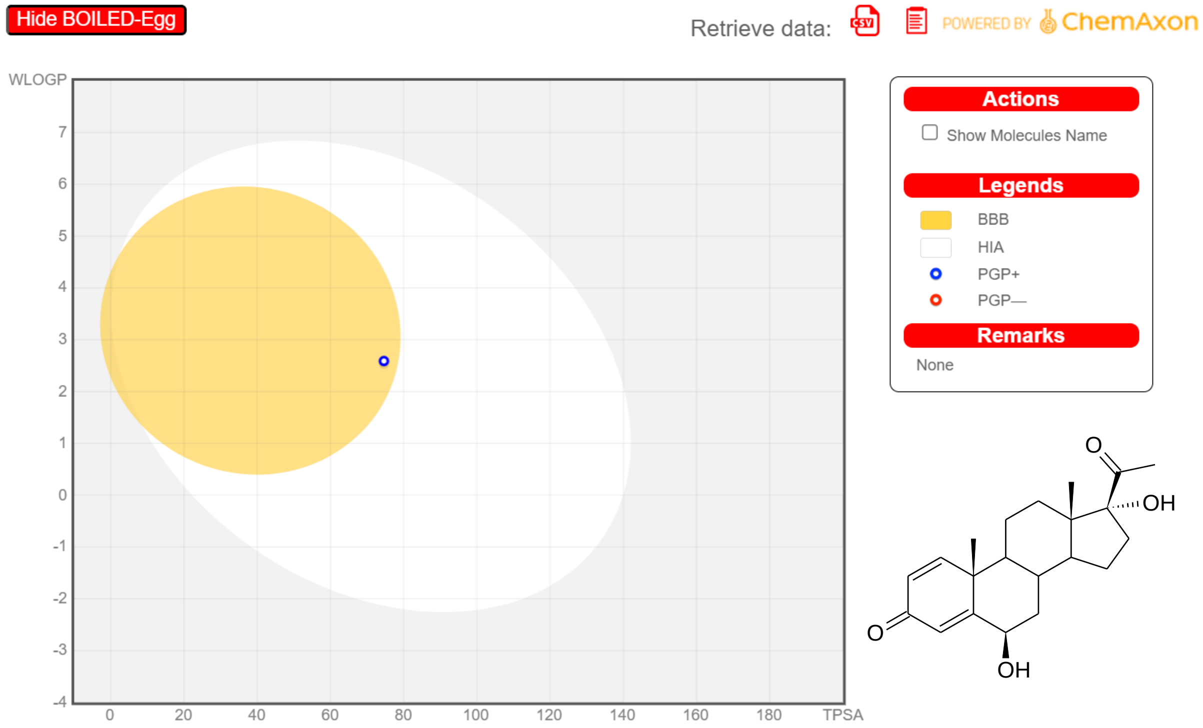

Figure S37. 6β,17α-dihydroxypregn-1,4-diene-3,20-dione (**6**) physicochemical and ADME parameters prediction using the SwissADME modelling

Molecule 1

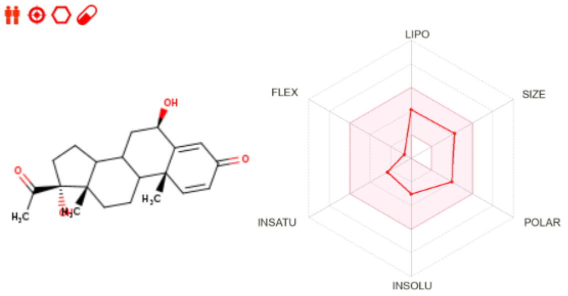

SMILES O=C1C=C[C@]2(C(=C1)[C@H](O)CC1C2CC[C@]2(C1CC[C@]2(O)C(=O)C)C)C

Physicochemical Properties	
Formula	C21H28O4
Molecular weight	344.44 g/mol
Num. heavy atoms	25
Num. arom. heavy atoms	0
Fraction Csp3	0.71
Num. rotatable bonds	1
Num. H-bond acceptors	4
Num. H-bond donors	2
Molar Refractivity	95.90
TPSA	74.60 Å²
**Lipophilicity**	
Log $P_{o/w}$ (iLOGP)	2.52
Log $P_{o/w}$ (XLOGP3)	1.74
Log $P_{o/w}$ (WLOGP)	2.59
Log $P_{o/w}$ (MLOGP)	2.13
Log $P_{o/w}$ (SILICOS-IT)	2.68
Consensus Log $P_{o/w}$	2.33

Water Solubility	
Log *S* (ESOL)	-3.01
Solubility	3.40e-01 mg/ml ; 9.87e-04 mol/l
Class	Soluble
Log *S* (Ali)	-2.92
Solubility	4.11e-01 mg/ml ; 1.19e-03 mol/l
Class	Soluble
Log *S* (SILICOS-IT)	-2.74
Solubility	6.33e-01 mg/ml ; 1.84e-03 mol/l
Class	Soluble
**Pharmacokinetics**	
GI absorption	High
BBB permeant	Yes
P-gp substrate	Yes
CYP1A2 inhibitor	No
CYP2C19 inhibitor	No
CYP2C9 inhibitor	No
CYP2D6 inhibitor	No
CYP3A4 inhibitor	No
Log K_p (skin permeation)	-7.17 cm/s
**Druglikeness**	
Lipinski	Yes; 0 violation
Ghose	Yes
Veber	Yes
Egan	Yes
Muegge	Yes
Bioavailability Score	0.55
**Medicinal Chemistry**	
PAINS	0 alert
Brenk	0 alert
Leadlikeness	Yes
Synthetic accessibility	5.15

Figure S38. Predicted Boiled-Egg plot from swissADME online web tool for 6β,17β-dihydroxyandrost-1,4-diene-3-one (**7**)

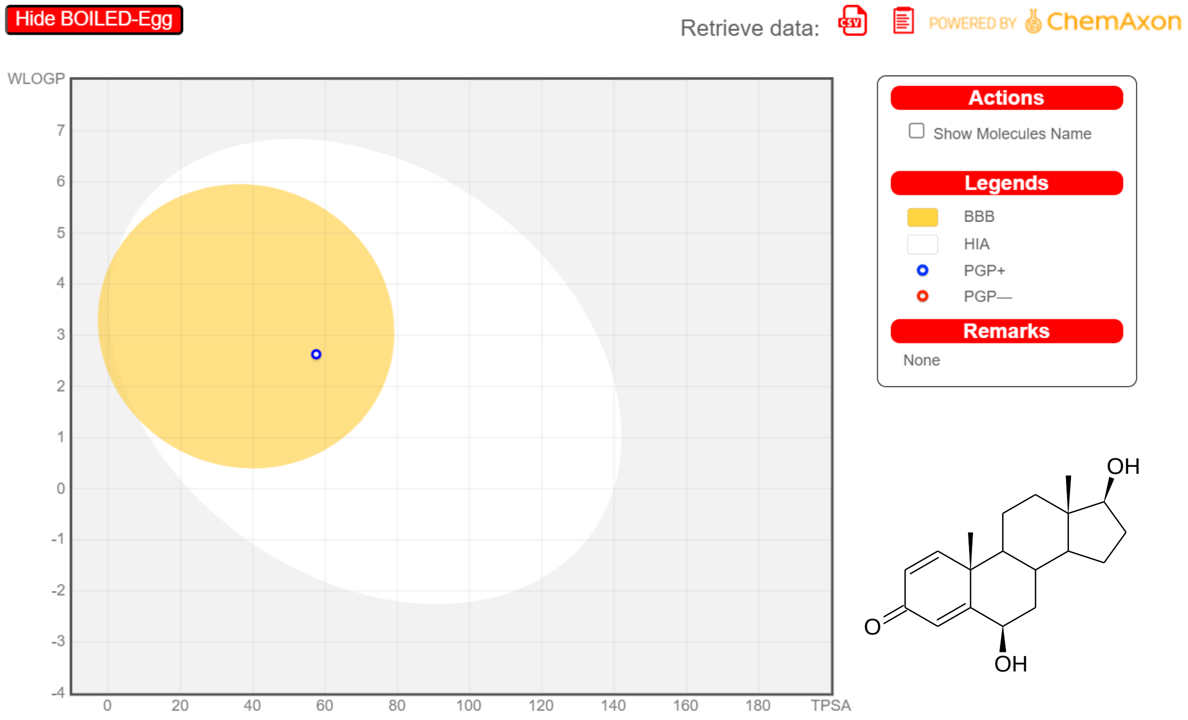

Figure S39. 6β,17β-dihydroxyandrost-1,4-diene-3-one (**7**) physicochemical and ADME parameters prediction using the SwissADME modelling

Molecule 1

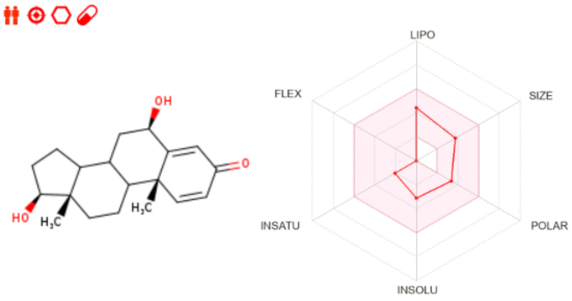

SMILES O=C1C=C[C@]2(C(=C1)[C@H](O)CC1C2CC[C@]2(C1CC[C@@H]2O)C)C

Physicochemical Properties	
Formula	C19H26O3
Molecular weight	302.41 g/mol
Num. heavy atoms	22
Num. arom. heavy atoms	0
Fraction Csp3	0.74
Num. rotatable bonds	0
Num. H-bond acceptors	3
Num. H-bond donors	2
Molar Refractivity	86.05
TPSA	57.53 Å²

Lipophilicity	
Log $P_{o/w}$ (iLOGP)	2.65
Log $P_{o/w}$ (XLOGP3)	2.24
Log $P_{o/w}$ (WLOGP)	2.63
Log $P_{o/w}$ (MLOGP)	2.62
Log $P_{o/w}$ (SILICOS-IT)	2.26
Consensus Log $P_{o/w}$	2.48

Water Solubility	
Log *S* (ESOL)	-3.13
Solubility	2.26e-01 mg/ml ; 7.48e-04 mol/l
Class	Soluble
Log *S* (Ali)	-3.08
Solubility	2.49e-01 mg/ml ; 8.25e-04 mol/l
Class	Soluble
Log *S* (SILICOS-IT)	-2.20
Solubility	1.89e+00 mg/ml ; 6.25e-03 mol/l
Class	Soluble

Pharmacokinetics	
GI absorption	High
BBB permeant	Yes
P-gp substrate	Yes
CYP1A2 inhibitor	No
CYP2C19 inhibitor	No
CYP2C9 inhibitor	No
CYP2D6 inhibitor	No
CYP3A4 inhibitor	No
Log K_p (skin permeation)	-6.55 cm/s

Druglikeness	
Lipinski	Yes; 0 violation
Ghose	Yes
Veber	Yes
Egan	Yes
Muegge	Yes
Bioavailability Score	0.55

Medicinal Chemistry	
PAINS	0 alert
Brenk	0 alert
Leadlikeness	Yes
Synthetic accessibility	5.03

Figure S40. ^{1}H NMR spectra of 12β,17α-dihydroxypregn-1,4-diene-3-one (**8**) (DMSO-d_6, 600 MHz)

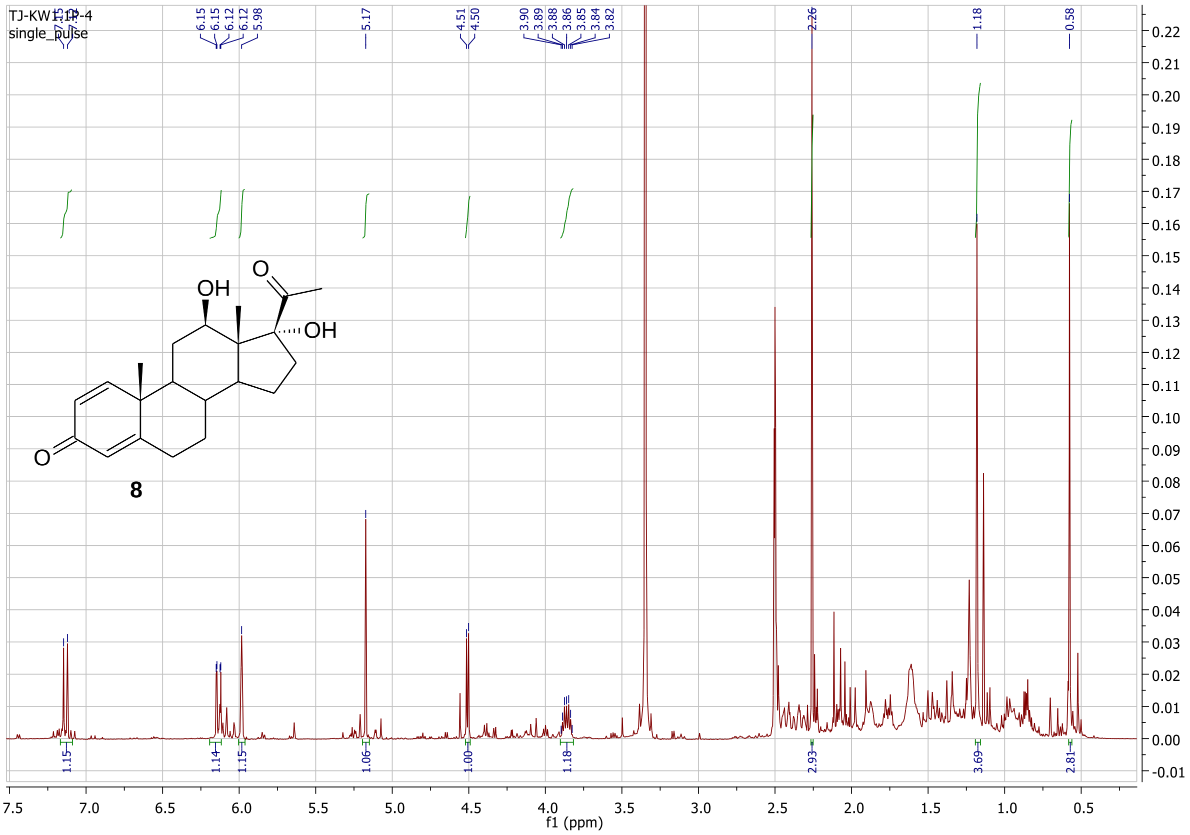

Figure S41. ^{13}C NMR spectra of 12β,17α-dihydroxypregn-1,4-diene-3-one (**8**) (DMSO-d_6, 151 MHz)

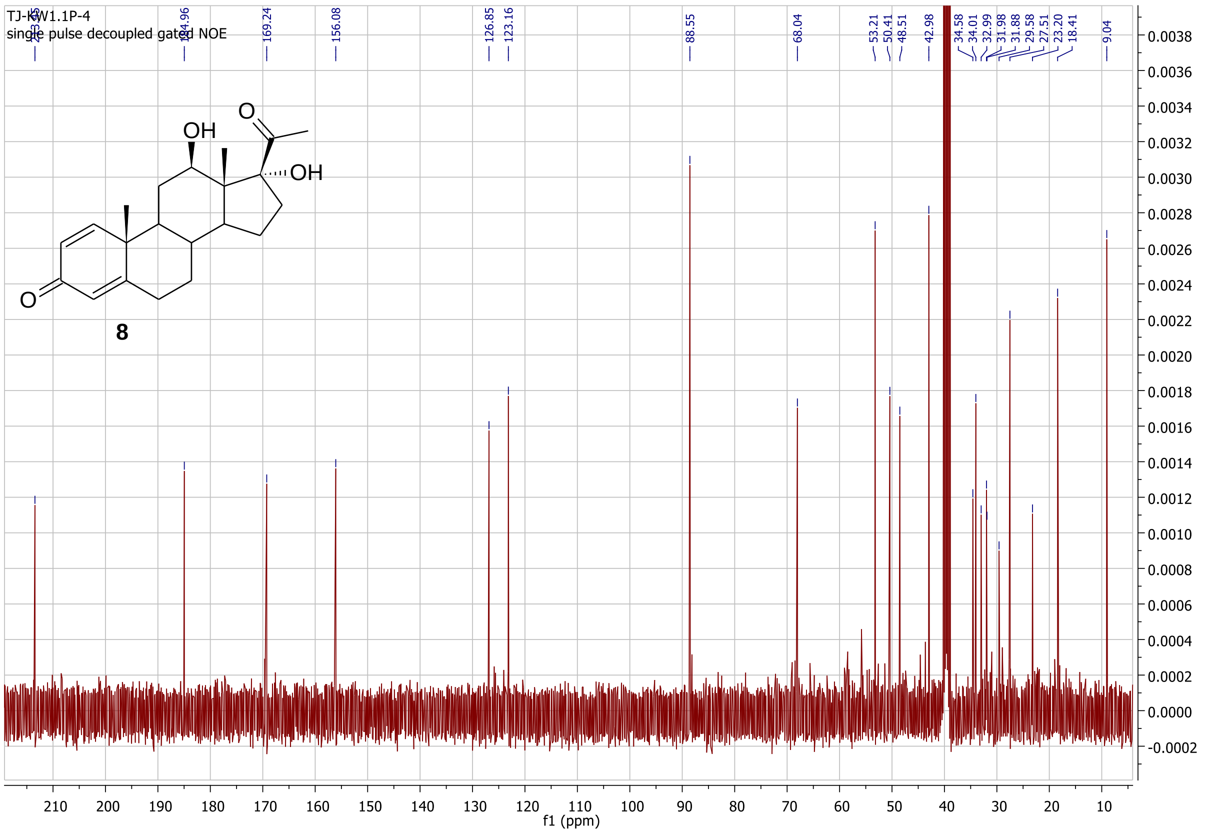

Figure S42. COSY spectrum of 12β,17α-dihydroxypregn-1,4-diene-3-one (**8**) (DMSO-d_6, 600 MHz)

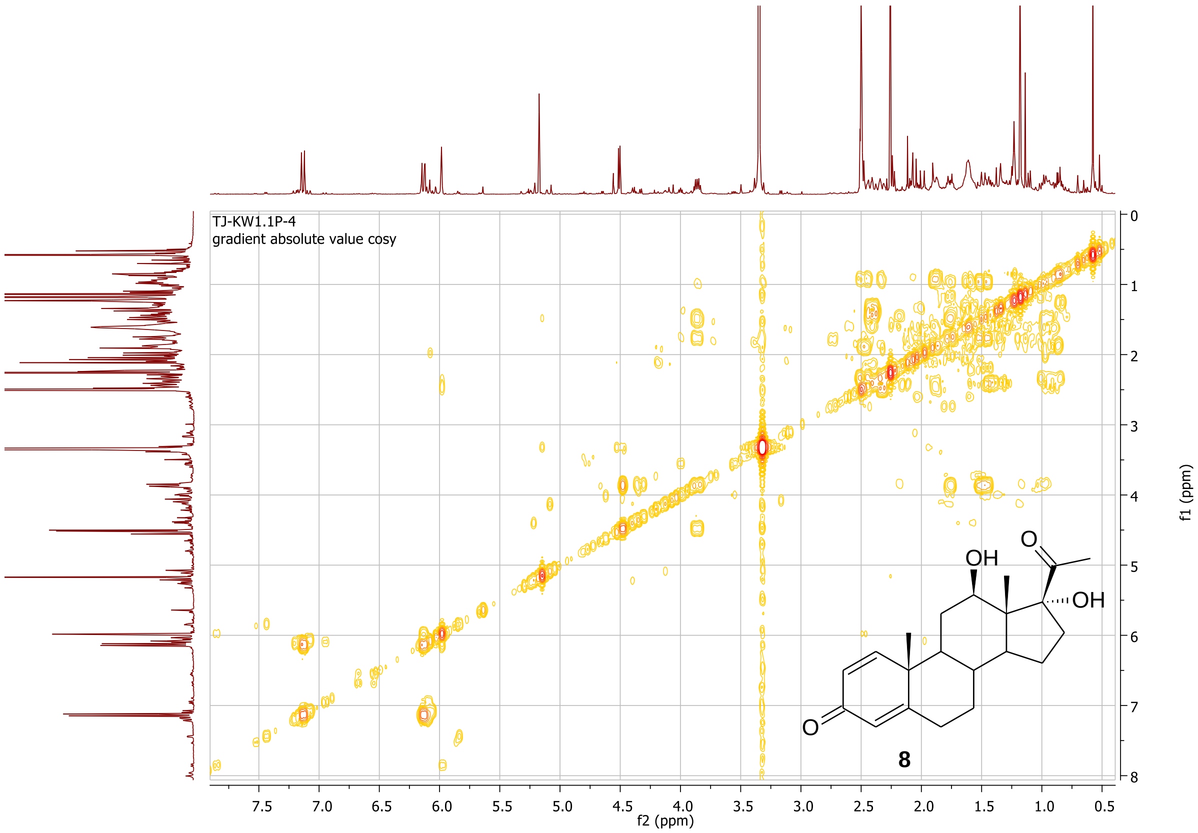

Figure S43. HSQC spectrum of 12β,17α-dihydroxypregn-1,4-diene-3-one (**8**) (DMSO-d_6, 600/151 MHz)

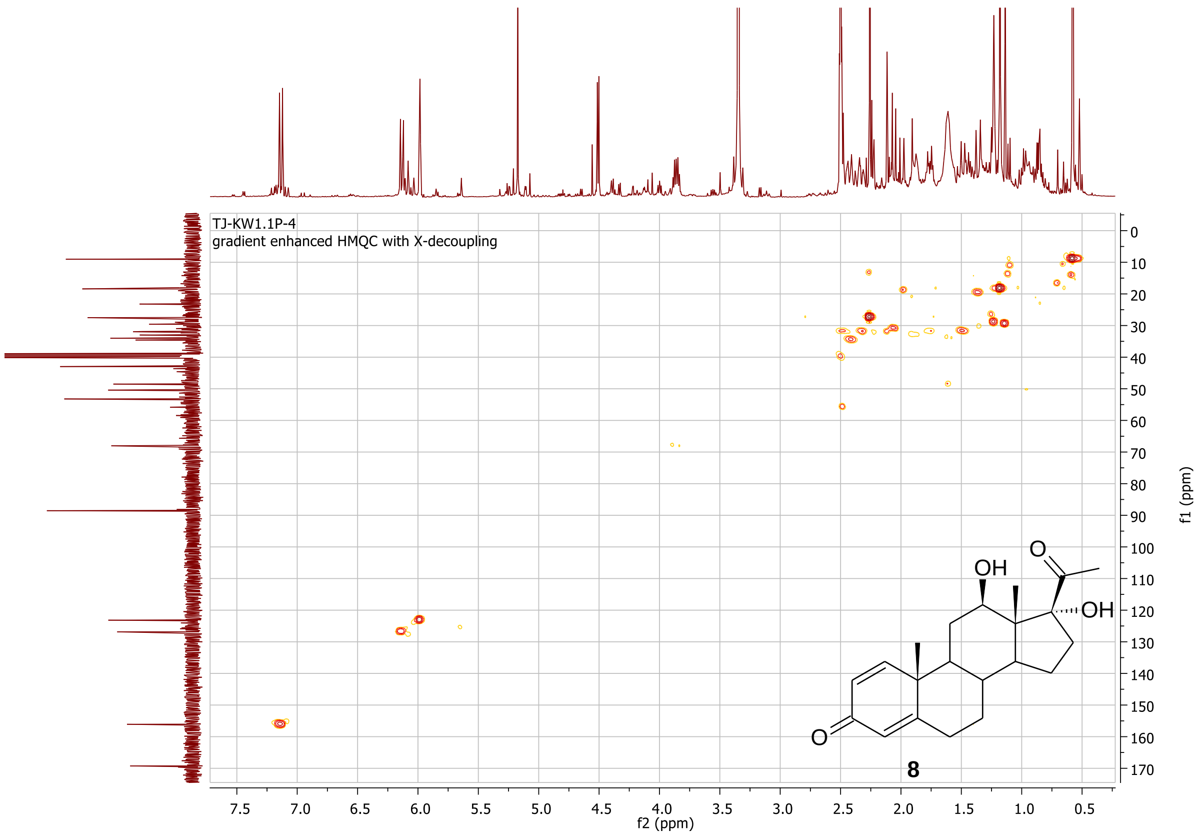

Figure S44. HMBC spectrum of 12β,17α-dihydroxypregn-1,4-diene-3-one (**8**) (DMSO-d_6, 600/151 MHz)

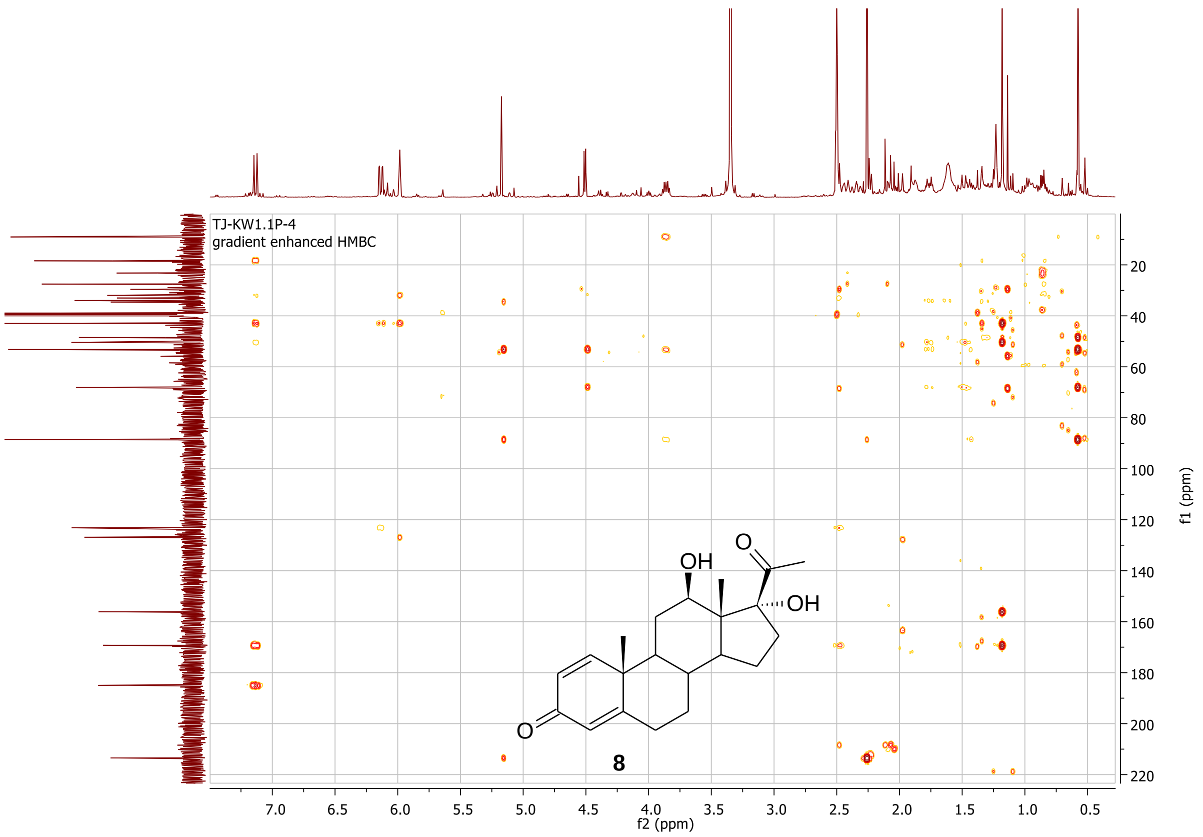

Figure S45. Predicted Boiled-Egg plot from swissADME online web tool for 12β,17α-dihydroxypregn-1,4-diene-3-one (**8**)

Molecule 1

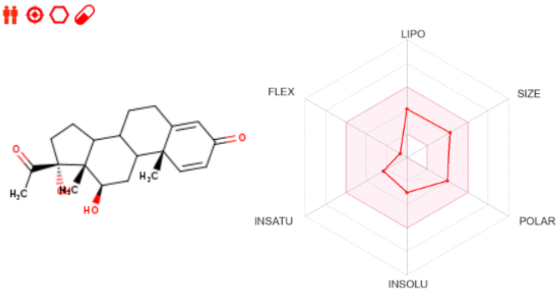

SMILES O=C1C=C[C@]2(C(=C1)CCC1C2C[C@@H](O)[C@]2(C1CC[C@]2(O)C(=O)C)C)C

Physicochemical Properties	
Formula	C21H28O4
Molecular weight	344.44 g/mol
Num. heavy atoms	25
Num. arom. heavy atoms	0
Fraction Csp3	0.71
Num. rotatable bonds	1
Num. H-bond acceptors	4
Num. H-bond donors	2
Molar Refractivity	95.90
TPSA	74.60 Å²

Lipophilicity	
Log $P_{o/w}$ (iLOGP)	2.79
Log $P_{o/w}$ (XLOGP3)	1.72
Log $P_{o/w}$ (WLOGP)	2.59
Log $P_{o/w}$ (MLOGP)	2.13
Log $P_{o/w}$ (SILICOS-IT)	2.68
Consensus Log $P_{o/w}$	2.38

Water Solubility	
Log *S* (ESOL)	-2.99
Solubility	3.50e-01 mg/ml ; 1.02e-03 mol/l
Class	Soluble
Log *S* (Ali)	-2.90
Solubility	4.31e-01 mg/ml ; 1.25e-03 mol/l
Class	Soluble
Log *S* (SILICOS-IT)	-2.74
Solubility	6.33e-01 mg/ml ; 1.84e-03 mol/l
Class	Soluble

Pharmacokinetics	
GI absorption	High
BBB permeant	Yes
P-gp substrate	Yes
CYP1A2 inhibitor	No
CYP2C19 inhibitor	No
CYP2C9 inhibitor	No
CYP2D6 inhibitor	No
CYP3A4 inhibitor	No
Log K_p (skin permeation)	-7.18 cm/s

Druglikeness	
Lipinski	Yes; 0 violation
Ghose	Yes
Veber	Yes
Egan	Yes
Muegge	Yes
Bioavailability Score	0.55

Medicinal Chemistry	
PAINS	0 alert
Brenk	0 alert
Leadlikeness	Yes
Synthetic accessibility	5.18

Figure S46. 12β,17α-dihydroxypregn-1,4-diene-3-one (**8**) physicochemical and ADME parameters prediction using the SwissADME modelling

Hide BOILED-Egg

Retrieve data: POWERED BY ChemAxon

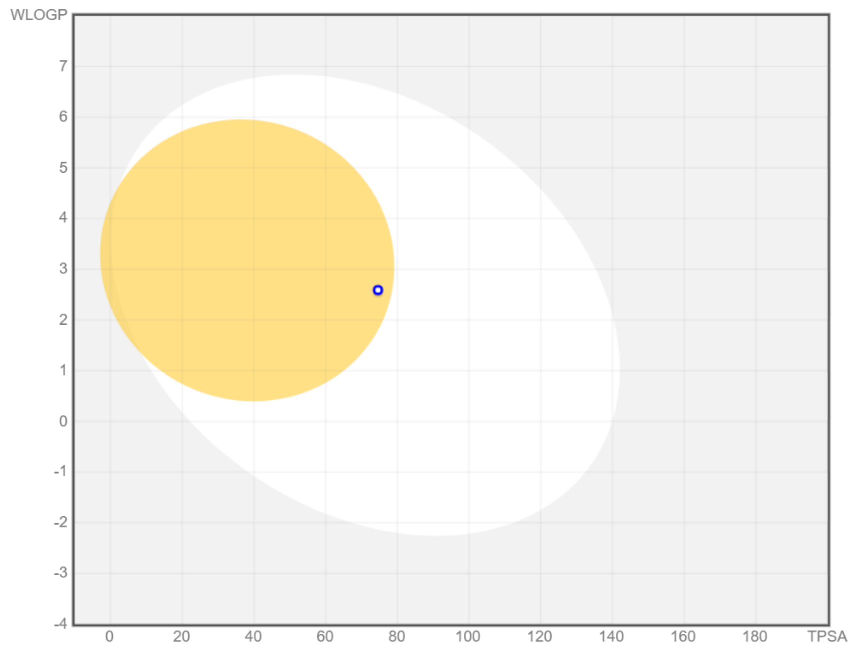

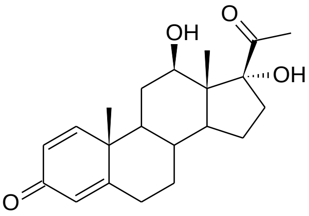

8
